# Drug Repurposing against KRAS Mutant G12C: A Machine Learning, Molecular Docking, and Molecular Dynamics Study

**DOI:** 10.3390/ijms24010669

**Published:** 2022-12-30

**Authors:** Tarapong Srisongkram, Natthida Weerapreeyakul

**Affiliations:** Division of Pharmaceutical Chemistry, Faculty of Pharmaceutical Sciences, Khon Kaen University, Khon Kaen 40002, Thailand

**Keywords:** NSCLC, drug repurposing, extreme gradient boosting, XGBoost, QSAR, drug repositioning, covalent drugs, sotorasib, adagrasib, afatinib, artificial intelligence

## Abstract

The Kirsten rat sarcoma viral G12C (KRAS^G12C^) protein is one of the most common mutations in non-small-cell lung cancer (NSCLC). KRAS^G12C^ inhibitors are promising for NSCLC treatment, but their weaker activity in resistant tumors is their drawback. This study aims to identify new KRAS^G12C^ inhibitors from among the FDA-approved covalent drugs by taking advantage of artificial intelligence. The machine learning models were constructed using an extreme gradient boosting (XGBoost) algorithm. The models can predict KRAS^G12C^ inhibitors well, with an accuracy score of validation = 0.85 and Q^2^_Ext_ = 0.76. From 67 FDA-covalent drugs, afatinib, dacomitinib, acalabrutinib, neratinib, zanubrutinib, dutasteride, and finasteride were predicted to be active inhibitors. Afatinib obtained the highest predictive log-inhibitory concentration at 50% (pIC_50_) value against KRAS^G12C^ protein close to the KRAS^G12C^ inhibitors. Only afatinib, neratinib, and zanubrutinib covalently bond at the active site like the KRAS^G12C^ inhibitors in the KRAS^G12C^ protein (PDB ID: 6OIM). Moreover, afatinib, neratinib, and zanubrutinib exhibited a distance deviation between the KRAS^G2C^ protein-ligand complex similar to the KRAS^G12C^ inhibitors. Therefore, afatinib, neratinib, and zanubrutinib could be used as drug candidates against the KRAS^G12C^ protein. This finding unfolds the benefit of artificial intelligence in drug repurposing against KRAS^G12C^ protein.

## 1. Introduction

Kirsten rat sarcoma viral oncogene (KRAS) is an intracellular membrane-bound GTPase enzyme that is highly mutated in non-small cell lung cancer (NSCLC) [1], colorectal cancer [2], and most solid tumors (e.g., pancreatic adenocarcinoma) [3]. Major KRAS mutations occur at codon 12 by changing guanine to aspartic acid (G12D), cysteine (G12C), or valine (G12V) [4,5]. The G12C is the highest KRAS mutation in NSCLC [6], while the G12D is the highest KRAS mutation in colorectal cancer [4]. The KRAS protein can initiate cell proliferation and the cell cycle by activating tyrosine kinase signaling pathways, including the RAF–MEK–ERK and PI3K–AKT–mTOR pathways [7]. The KRAS protein functions as a molecular switch by alternating between guanosine diphosphate (GDP)-bound (inactive) and guanosine triphosphate (GTP)-bound (active) forms to initiate the tyrosine kinase signal transduction [8]. Previously, the KRAS protein was considered a non-druggable target because: (1) the KRAS protein strongly binds to GTP and GDP in a picomolar affinity, which makes it very difficult to design a competitive inhibitor. (2) The KRAS protein lacks a deep surface hydrophobic pocket in an active site, which hinders the development of competitive nucleotide inhibitors [9]. Recently, a new allosteric site of KRAS protein was revealed [10]. Subsequently, the sotorasib and adagrasib–U.S. FDA-approved drugs against KRAS^G12C^ protein–were discovered due to their ability to covalently bind to cysteine 12 of KRAS^G12C^ [8,11]. Sotorasib is now currently used as a new targeted therapy for NSCLC patients with a KRAS^G12C^ mutation [12].

Despite the benefit of KRAS^G12C^ inhibitors, a recent report concluded that a vast majority of KRAS^G12C^ mutation lung adenocarcinomas, the most common group of NSCLC patients, tend to resist both sotorasib and adagrasib after the treatment. This resistance by the newly synthesized KRAS protein can occur from the activation of the epidermal growth factor receptor (EGFR) and PI3K–AKT–mTOR pathway in cancer cells [13]. Therefore, identifying new drugs for KRAS^G12C^ is still needed to extend the drug choice and, importantly, to improve the clinical outcome.

Drug repurposing is a strategy for repositioning already approved drugs in a new pharmacological indication [14]. This strategy is not new; in fact, it has been successfully utilized in the case of: (1) remdesivir for SAR-CoV-2 infection [15], (2) sildenafil for erectile dysfunction [16], (3) metformin for adjuvant cancer therapy [17], (4) doxorubicin for breast cancer [14], and (5) clarithromycin for colon cancer [18]. A drug repurposing approach has benefit over newly designed compounds as it can be more rapidly developed since a lot of preclinical and clinical data have already been determined [19]. Drug repurposing is well-suited for the quickly emerging disease for which current therapeutics are unavailable or resistant.

Computational approaches like machine learning [20], molecular docking [21], and virtual screening [22] can be used to identify drug candidates against the KRAS^G12C^ protein. Strategies to inhibit the KRAS^G12C^ protein usually involve covalent bonding of the drug and cysteine residue 12 of chain A of the KRAS^G12C^ protein. One study has predicted lead-like analogs for KRAS^G12C^ inhibitors from the PubChem database by using a virtual screening technique, but those compounds are still not eligible in the clinic [21]. Thus, a drug repurposing study for the KRAS^G12C^ protein should be conducted to facilitate the emergency need for drugs against the KRAS^G12C^ protein.

In this paper, we opted to identify a new drug of choice for NSCLC patients with a KRAS^G12C^ mutation using machine learning approaches. New drugs were screened from reported FDA-approved covalent drugs [23]. The extreme gradient boosting (XGBoost) algorithm was selected because it involves faster run time, lower computational power, and higher average accuracy than random forest and single-task neural network algorithms for quantitative structure–activity relationship (QSAR) analyses [24]. Our other ongoing research is benchmarking the performance of XGBoost and other ML algorithms for the accurate prediction of QSAR models. Furthermore, molecular docking and molecular dynamics have been applied to accurately predict drug–protein interactions and provide insights into binding modes of compounds of interest [25]. The KRAS^G12C^ inhibitor requires covalent bonding with cysteine 12; hence, we determined the possible covalent bonding of the predicted drugs using covalent docking. In the last step, we studied the dynamic movement between the predicted drugs and KRAS^G12C^ protein using molecular dynamics simulation.

## 2. Results and Discussion

### 2.1. Chemical Distribution of the Datasets

**Figure 1:**
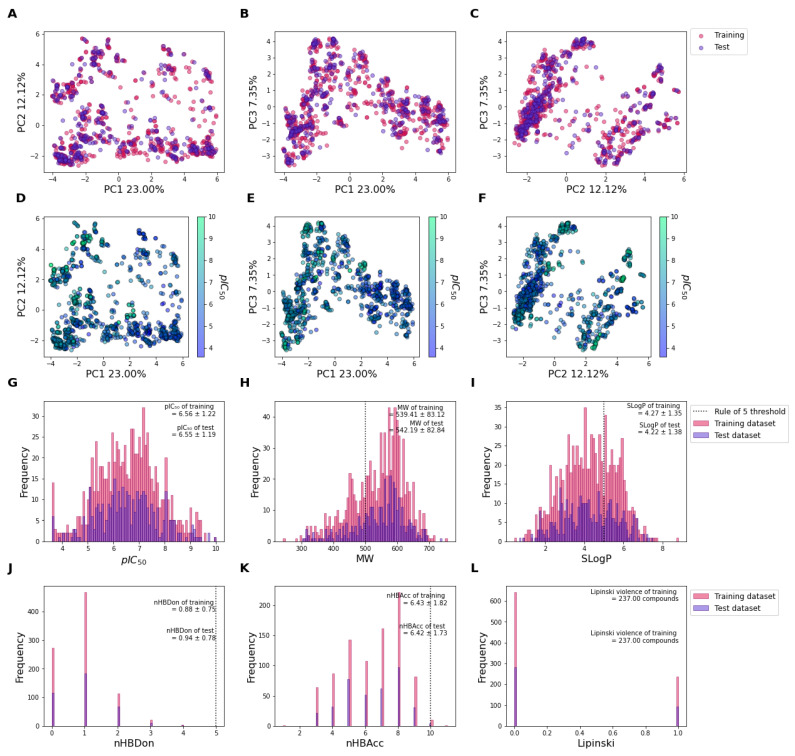
Principal component analysis (PCA) and chemical distribution of KRAS^G12C^ datasets. Score plots of (**A**) PC1 and PC2; (**B**) PC1 and PC3; and (**C**) PC2 and PC3 were divided by training (pink circles) and test (purple circles) datasets. Numbers on the x and y axis indicate the percentage of explained variance for each PC. Score plots of (**D**) PC1 and PC2; (**E**) PC1 and PC3; and (**F**) PC2 and PC3 were labeled based on the pIC_50_ values, where the light green and light purple indicate the high and low pIC_50_, respectively. Histograms of (**G**) pIC_50_; (**H**) molecular weight (MW); (**I**) LogP (SLogP); (**J**) the number of H-bond donors (nHBDon); (**K**) the number of H-bond acceptors (nHAcc); and (**L**) the number of Lipinski’s rule violations were divided by training (pink bars) and test (purple bars) datasets. The dashed line indicates the rule of five (Lipinski’s rule) threshold.

The predictive ligand affinity against KRAS^G12C^ protein was built based on the previously published experimental data collected from ChEMBL and BindingDB databases [26,27]. A total of 1255 compounds were divided into 878 training compounds and 377 test compounds (Figure 1). Figure 1A–C represents the score plot of the principal component (PC) of training (pink circle) and test (purple circles) between PC1 and PC2, PC1 and PC3, and PC2 and PC3, respectively. The principal components were calculated based on the chemical fingerprints (PubChem fingerprint and Substructure fingerprint count) of each compound. Note that the training and test datasets show similar chemical distributions for which no compound exhibits extreme delineate outside the centric distribution. Moreover, the distribution of pIC_50_ values was virtualized based on using the same score plots between PC1 and PC2, PC1 and PC3, and PC2 and PC3 in Figure 1D–F, respectively. The principal component analysis (PCA) shows that compounds can be divided into two clusters based on the distribution of low pIC_50_ values (pIC_50_ < 6; light purple) and high pIC_50_ (pIC_50_ > 6; light green), especially in Figure 1F. This indicates that the fingerprints could correlate with the pIC_50_ of the KRAS^G12C^ inhibitory activity. 

The drug-likeness properties of training and test datasets were evaluated by using the Lipinski rule of five. Figure 1G represents the overall pIC_50_ value of the training (pink), and test (purple) datasets, respectively. The average pIC_50_ of the training and test dataset was 6.56 ± 1.22 and 6.55 ± 1.19, respectively. The molecular weight of the training and test datasets was 539.41 ± 83.12 and 542.19 ± 82.84, respectively (Figure 1H). The average molecular weight of both datasets is higher than 500 Da, which violates the Lipinski rule of five. The LogP (SLogP) of the training and test datasets was 4.27 ± 1.35 and 4.22 ± 1.38, respectively (Figure 1I). The average LogP of both datasets conformed with the Lipinski rule of five (LogP < 5). The average number of hydrogen-bond donors in both datasets is 0.88 ± 0.75 and 0.94 ± 0.78, respectively (Figure 1J). No compounds had hydrogen-bond donors higher than five, which means the number of hydrogen-bond donors passed the criteria of the Lipinski rule of five. The average number of hydrogen-bond acceptors of both datasets is 6.43 ± 1.82 and 6.42 ± 1.73, respectively (Figure 1K). The average number of hydrogen-bond acceptors of both datasets agreed with the criteria of the Lipinski rule of five. Overall, 237 compounds of the training dataset (27%) and 94 compounds of the test dataset (25%) failed to meet the Lipinski rule of five criteria (Figure 1L). Thus, over 73% and 75% of the compounds in both datasets conform with the drug-likeness criteria and can be presented as orally active compounds.

### 2.2. Predictive KRAS pIC_50_ ML Model Based on XGBoost Algorithm of All Molecular Fingerprints

**Figure 2:**
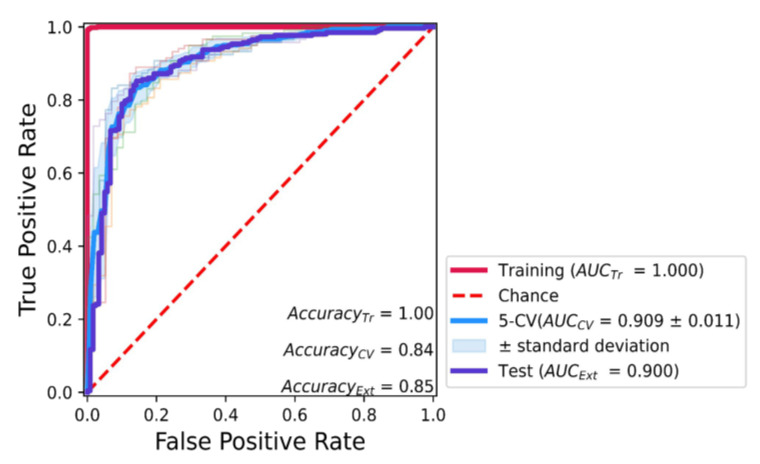
Classification model for predicting against KRAS^G2C^ ligand affinity. The red, blue, and purple solid lines indicate the training, 5-fold cross-validation (5-CV), and test datasets, respectively. The red dashed line indicates the random decision by chance of the model. The light blue area indicates the standard deviation of 5-fold cross-validation.

**Figure 3:**
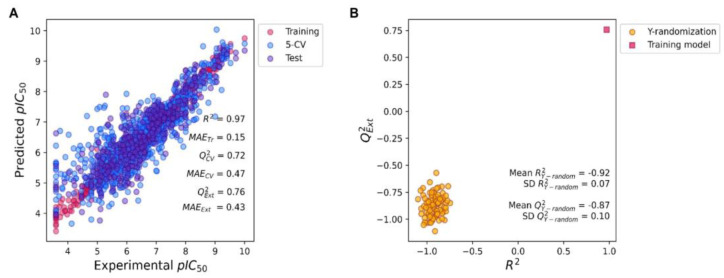
Regression model for predicting pIC_50_ values against KRAS^G2C^. (**A**) Regression XGBoost model. The red, blue, and purple circles indicate the training dataset, 5-fold cross-validation (5-CV), and test datasets, respectively. (**B**) The Y-randomization analysis of the regression model. The yellow circles and red squares represent the Y-randomization models (*n* = 100) and the original model, respectively.

The classification and regression machine learning models were built by using the XGBoost algorithm with the PubChem fingerprint and Substructure fingerprint count (Figure 2 and Figure 3). The machine learning models were optimized by using 5-cross validation (CV) hyperparameter searching. For the classification model, a pIC_50_ value higher than or equal to six was considered active, while a pIC_50_ value less than six was considered inactive. Results showed that the classification model had the accuracy score of the training dataset (Accuracy_Tr_) at 1.00, 5-fold cross-validation (5-CV; Accuracy_CV_) at 0.84, and the test set (Accuracy_Ext_) at 0.85 (Figure 2). Moreover, the area under the curve of the receiver operating characteristic curve of the training dataset (AUC_Tr_) was at 1.000, 5-CV (AUC_CV_) at 0.909 ± 0.011; and at 0.900 for the test dataset (AUC_Ext_) (Figure 2). This result indicates that the classification provided an accurate prediction for classifying active and inactive molecules (All Accuracy ≥ 0.84, and AUC ≥ 0.900).

Figure 3 presents a regression model for predicting the pIC_50_ value against the KRAS^G12C^ protein. The result shows that the model had a coefficient of determination of the training dataset (R^2^) of 0.97 or 97% variance explanation with absolute mean error (MAE_Tr_) at 0.15 (Figure 3A, red circle). This result indicates that the mean error between the experimental value and prediction value was 0.17, which means our model had excellent goodness-of-fit (R^2^ > 0.8, and MAE_Tr_ < 0.5) to learn and explain the training dataset. Additionally, the model was internally validated by 5-CV of the training dataset (Figure 3A, blue circle). The result shows that the 5-CV also yields a coefficient of determination of the cross-validation (Q^2^_CV_) of 0.72 with a mean absolute error of cross-validation (MAE_CV_) of 0.47. This indicates that the correlation found in the model is strong and accurate enough that even the changing of the training dataset did not affect the accuracy of the model (acceptable Q^2^_CV_ is higher than 0.5). Moreover, the prediction ability of the model was externally validated by using an external test dataset (Figure 3B, purple circle). The result shows that the model can accurately predict the unlearned dataset with the coefficient of determination of the external test dataset ((Q^2^_Ext_) of 0.76 with the mean absolute error of the external test dataset (MAE_Ext_) of 0.43). The acceptable value for Q^2^_Ext_ is higher or equal to 0.5, while the MAE_Ext_ is less than 0.5. Thus, our model conforms with all the criteria, indicating that the model contains excellent goodness-of-fit and high predictive power.

We also tested the robustness of the regression model by using the Y-randomization approach where the pIC_50_ values (y values) had been randomly changed from the original y values (Figure 3B). If the Y-randomization model possesses a similar value of R^2^ or Q^2^_Ext_ to the original value, this means that our model has a false correlation between the fingerprints (x) and y pair (unreliable or false relationship) and the model will not be acceptable. However, if the Y-randomization model possesses a very different value of R^2^ or Q^2^_Ext_ to the original value, this means that our model explains the true correlation between the x and y pair, resulting in high model robustness, in which case the model will be accepted. The result shows that the average of R^2^ (R^2^_Y-random_) and Q^2^_Ext_ (Q^2^_Y-random_) were −0.92 ± 0.07 and −0.87 ± 0.10 (*n* = 100), respectively (Figure 3B, yellow circle), while the original R^2^ and Q^2^_Ext_ values were 0.97 and 0.76, respectively (Figure 3B, red square). This result indicates the vast difference between the original model and the Y-randomization models, which denotes that our regression model is vigorous in its correlation between fingerprints and the pIC_50_ values against the KRAS^G12C^ protein.

### 2.3. KRAS Inhibitor Structural Importance Identified by SHAP Algorithm

**Figure 4:**
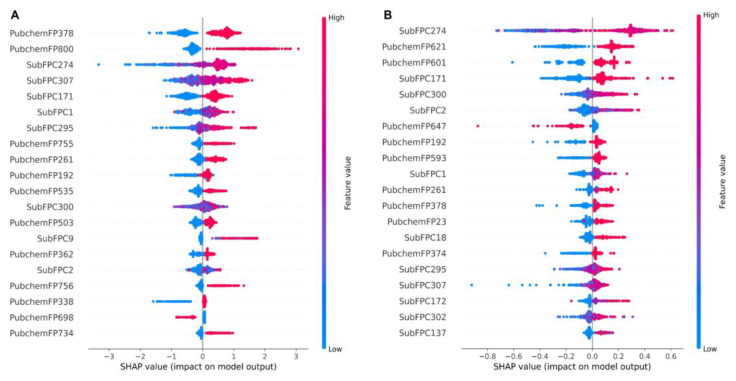
Importance features extracted from machine learning models by using the SHAP algorithm. (**A**) SHAP importance features of the classification XGBoost model and (**B**) SHAP importance features of the regression XGBoost model. The red and blue colors indicate the high and low fingerprint values, respectively.

The important structures that are relevant to the prediction outcome were identified by computing Shapley Additive explanations’ (SHAP) feature importance. The SHAP values examine the effect of each molecular fingerprint on the model’s output. These values are separately calculated for each molecule (local explanation) and then built up to explain a global effect of each molecular fingerprint on the model’s output [28]. Figure 4 demonstrates the top 20 important structures of both the classification and the regression models (Figure 4A,B, respectively). The result shows that within the top 20 molecular fingerprints, 10 molecular fingerprints are similar between both models. The fingerprints of similar importance ranged by the SHAP score of the classification model included: PubChemFP378 (C(~N)(:C)(:N)), SubFPC274 (aromaticity), SubFPC307 (chiral center), SubFPC171 (aryl chloride), SubFPC1 (primary carbon), SubFPC295 (C-O, N, or S bond), PubChemFP261 (≥4 aromatic rings), PubChemFP192 (≥3 any ring size 6), SubFPC300 (1,3-tautomerizable), and SubFPC2 (secondary carbon) (Table 1). Moreover, the top five molecular fingerprints of the classification model were PubChemFP378, PubChemFP800 (CC1CC(N)CCC1), SubFPC274, SubFPC307, and SubFPC171, while the top five of the molecular fingerprints regression model were SubFPC274, PubChemFP621 (N-C:C:C:N), PubChemFP601 (N-C:C:C-N), SubFPC171, and SubFPC300. This result suggests that, if a molecule contains the PubChemFP378, PubChemFP800, SubFPC274, SubFPC307, and SubFPC171, it will probably be classified as active against the KRAS^G12C^ protein. Additionally, if a molecule contains SubFPC274, PubChemFP621, Pubchem601, SubFPC171, and SubFPC300, it could have a high pIC_50_ value against the KRAS^G12C^ protein. Nevertheless, the total number of fingerprints is 1188, which means the models can still use other fingerprints in the calculation of the model output. Conversely, the unmentioned fingerprints are also required for the model prediction to perform fully. However, further study should carefully reduce the dimensions of the fingerprint to reduce the model complexity without reducing the model’s performance.

### 2.4. Applicability Domain

**Figure 5:**
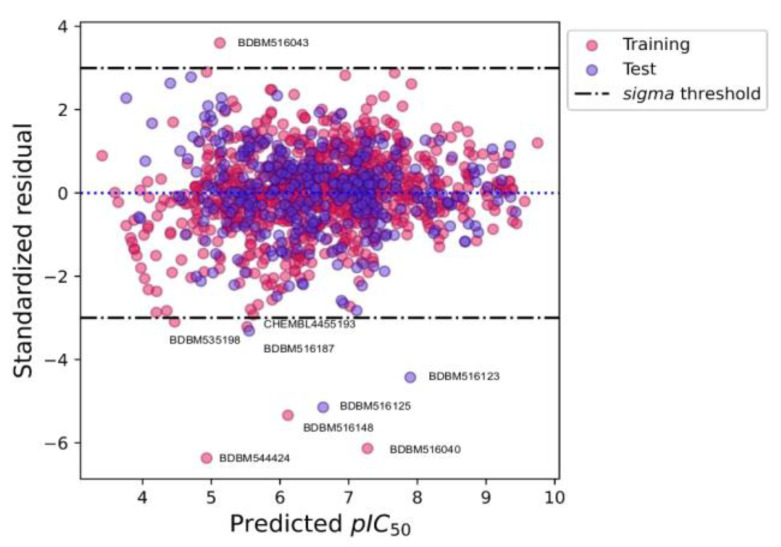
Standardized residual and predicted pIC_50_ plot. The pink and purple circles were the training and test datasets, respectively. The black dashed-dotted lines were the standardized residual outlier threshold (±3 standardized residual). Only outlier compounds were annotated in this plot.

The machine learning model cannot predict all the chemical compounds. The model will have a highly reliable prediction with the chemicals used in the training process. The evaluation of chemical structures with reliable or unreliable prediction is called the applicability domain of the model [29]. In this work, the applicability domain of the regression model was used to delineate the compounds that could not give a reliable prediction. The applicability domain was evaluated by using standardized residual plot analysis. The standardized residual for each compound was calculated based on the difference between the prediction value and experimental value compared to the mean difference between the prediction value and experimental value of all compounds, which was then divided by the standard deviation of the difference between the prediction value and experimental value of all compounds. The compound that has standardized residual higher than ±3 is considered an unreliable prediction. Figure 5 depicts the residual plot of the model output. The result found that the model has six unreliable predictions from the training and three unreliable predictions from the test dataset (Figure 5, black dashed-dotted lines). The 6 compounds from the training dataset included BDBM516043, CHEMBL445193, BDBM535198, BDBM516148, BDBM544424, and BDBM516040, while the 3 compounds from the test dataset were BDBM516187, BDBM616123, and BDBM516125. The unreliable prediction compounds can be indicated as outliers. In addition, the standardized residuals of low predicted pIC_50_ values (pIC_50_ < 6) had a high variance, but when the predicted pIC_50_ values increased, the residuals were also reduced to around zero (Figure 5, dashed blue line). This result indicates that the model more accurately predicts the high pIC_50_ values than the low pIC_50_ values. 

**Figure 6:**
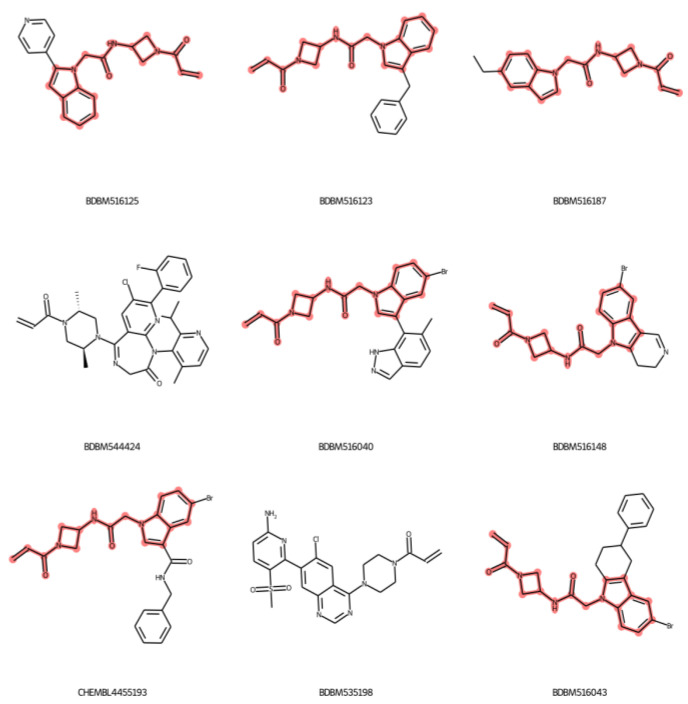
Chemical structures of outliers determined by the compounds whose standardized residual value exceeded ±3. The red color highlights the similar structures of the outliers.

The chemical structure of the nine outliers are shown in Figure 6. A similar substructure found in seven out of the nine outlier compounds is represented in the red color highlight. The core of the substructure includes an indole ring, acetamide, azetidine, and acrolein warhead functional groups. However, the acrolein warhead is the functional group that is required for covalent bonding. Thus, the data suggests that our model cannot predict the molecule that contains the indole ring with acetamide and azetidine functional groups.

### 2.5. Prediction of FDA-Approved Drugs against KRAS^G12C^ Protein

Table 2 shows the predicted compounds from the machine learning models. The covalent drugs were obtained from the covalent drugs database [23]. We found that 7 out of 67 compounds, including afatinib, dacomitinib, acalabrutinib, neratinib, zanubrutinib, dutasteride, and finasteride, were predicted to be in the active class from the classification model. In addition, sotorasib and adagrasib, which are FDA-approved drugs against KRAS^G12C^, were also predicted to be the active class, which confirms the predictive ability of the model. The predicted pIC_50_ of all compounds was also calculated. The results found that the predicted pIC_50_ values of sotorasib and adagrasib were 6.95 and 8.02, which are different from their experimental pIC_50_ values of 0.57 and 0.28, respectively. The mean absolute error of those two predictions was 0.43, which is acceptable compared to the model performance (all MAE values less than 0.5). We found that afatinib had the highest predicted pIC_50_ value (7.43) compared to dacomitinib (7.32), acalabrutinib (6.75), neratinib (6.68), zanubrutinib (6.49), dutasteride (5.94), and finasteride (5.05). This result implied that afatinib, dacomitinib, acalabrutinib, neratinib, and zanubrutinib have predicted pIC_50_ values higher than six, which is promising for further analyses. 

**Figure 7:**
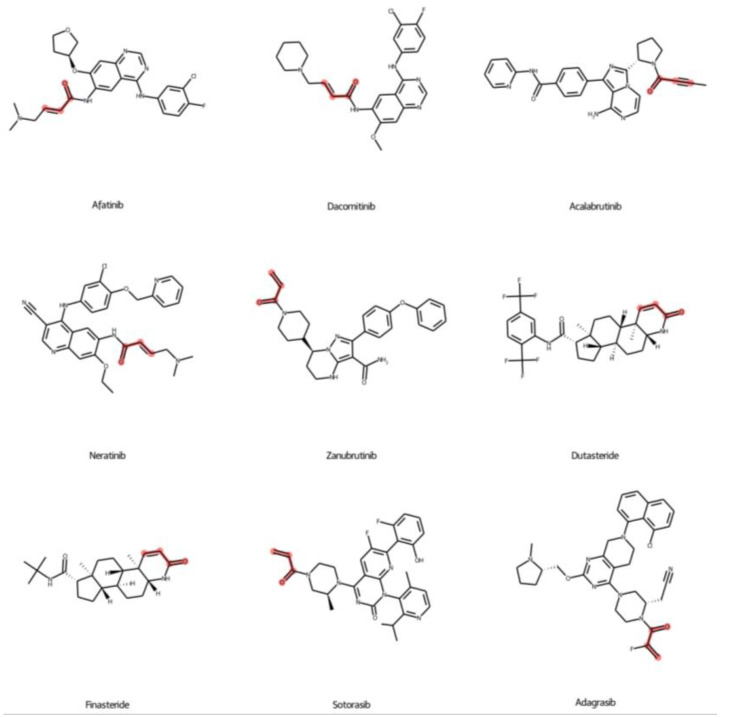
Predicted active compounds with a Michael covalent warhead group. The covalent warhead groups are highlighted in red color. The sotorasib and adagrasib are approved FDA KRAS^G12C^ inhibitors.

Additionally, the difference between primary targets and primary indications of the predicted compounds is also related to the predicted pIC_50_ values. The primary targets and indications of afatinib, dacomitinib, and neratinib are EGFR and NSCLC, while the primary targets and indications of acalabrutinib and zanubrutinib are Bruton tyrosine kinase (BTK) and mantle cell lymphoma (MCL), respectively (Table 2). On the other hand, the primary targets and primary indications of dutasteride and finasteride are 5α-reductase inhibitors and benign prostate hyperplasia (BPH) (Table 2), which is not correlated with the kinase protein. The chemical structures of predicted compounds were also investigated in Figure 7. All seven compounds contain a Michael covalent warhead (R-C=C-C(=O)-R; red color highlight) in the molecule (Figure 7). Only zanubrutinib contains the Michael warhead at the terminal of its structure, similar to sotorasib and adagrasib. Afatinib, dacomitinib, and neratinib have the R group attached to the Michael warhead. On the other hand, the Michael warhead of dutasteride and finasteride are in the ring forms, which may make them difficult to change the structure pose when binding with the KRAS protein.

### 2.6. Covalent Docking of Predicted Compounds against KRAS^G12C^ Protein

**Figure 8:**
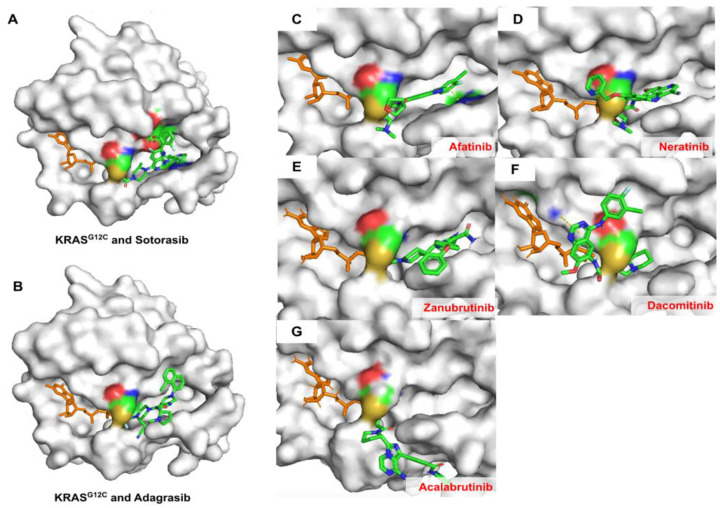
Covalent docking of all 7 compounds with KRAS^G12C^ (PDB: 6OIM). The compounds include (**A**) sotorasib, (**B**) adagrasib, (**C**) afatinib, (**D**) neratinib, (**E**) zanubrutinib, (**F**) dacomitinib, and (**G**) acalabrutinib. The predicted compounds are represented in green, blue, and red colors based on carbon, nitrogen, and oxygen atoms, respectively. The guanosine diphosphate (GDP) is presented in orange color while the KRAS^G12C^ is represented in white color. The cysteine residue 12 is represented in yellow color on the protein surface.

Covalent docking was done to evaluate the covalent interaction between the predicted drugs and the KRAS^G12C^ protein (PDB: 6OIM). Figure 8 depicts the 5 predicted drugs, including afatinib, dacomitinib, acalabrutinib, neratinib, and zanubrutinib, that can covalently bond with the cysteine 12 residue in a similar manner to sotorasib and adagrasib. Sotorasib and adagrasib were used as positive controls (Figure 8A,B). The result also demonstrated that afatinib, neratinib, and zanubrutinib bind in the same allosteric pocket site of KRAS protein as sotorasib and adagrasib (Figure 8C–E). In contrast, dacomitinib and acalabrutinib covalently bond with the cysteine 12 residue on the outside of the allosteric pocket (Figure 8F,G). This result suggests that afatinib, neratinib, and zanubrutinib could inhibit the KRAS^G12C^ protein via a covalent bond with the cysteine 12 residue.

### 2.7. Molecular Dynamics Simulation of FDA-Approved Drugs and KRAS G12C Protein

**Figure 9:**
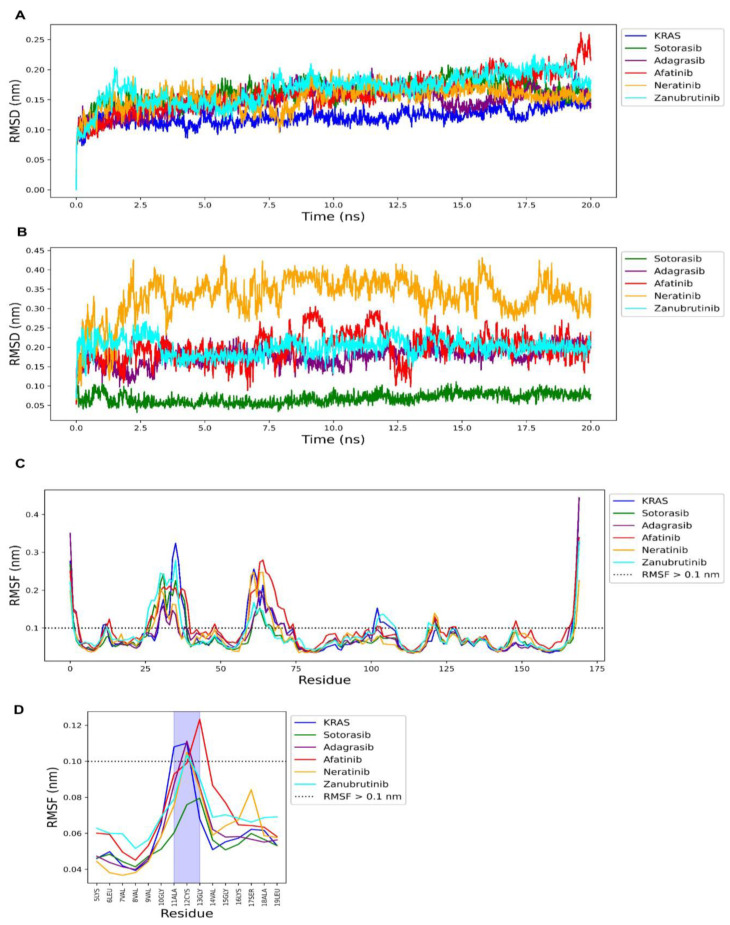
Molecular dynamics simulations of sotorasib, adagrasib, afatinib, neratinib, and zanubrutinib. (**A**) Time series of the distance of c-alpha atoms of KRAS^G12C^ protein when interacting with predicted drugs; and (**B**) time series of the distance of predicted drugs when interacting with the KRAS^G12C^ protein. (**C**) Amino acid distance fluctuation when interacting with predicted drugs; and (**D**) amino acid distance fluctuation at cysteine 12. The KRAS protein, sotorasib, adagrasib, afatinib, neratinib, and zanubrutinib are represented in blue, green, purple, red, orange, and cyan colors, respectively.

The drawback of traditional molecular docking is that it treats the protein as a static object when, in reality, proteins have dynamic movement [25]. To investigate the stability of protein and drug complexes, we also performed molecular dynamic simulation. Figure 9A shows the root mean square deviation (RMSD) of the distance of the alpha carbon (c-alpha) of the KRAS^G12C^ protein after complexing with the drug molecules in an explicit solvent compared with its initial form. The RMSD is a necessary parameter for investigating the stable conformation in drug–protein complexation. A lower RMSD of protein and drug indicates a high conformation of that complex. The results found that afatinib (red), neratinib (orange), and zanubrutinib (cyan) had RMSDs of 0.16 ± 0.03, 0.15 ± 0.02, 0.17 ± 0.02 nm, respectively, which are less than 0.2 nm (2 Å), similar to the RMSD of sotorasib (green; RMSD = 0.16 ± 0.02) and adagrasib (purple; RMSD = 0.15 ± 0.02) (Figure 9A). Figure 9B shows the RMSD of drug atoms during complexation. The result shows that the conformation of sotorasib is the most stable with an RMSD value less than 0.1 nm or 1 Å (0.06 ±0.01 nm), followed by adagrasib (RMSD = 0.17 ± 0.02 nm), afatinib (RMSD = 0.19 nm ± 0.04), neratinib (RMSD = 0.33 ± 0.05), and zanubrutinib (RMSD = 0.2 ± 0.02 nm). This result showed that the ligand conformations of afatinib and zanubrutinib are more stable than for neratinib. 

Fluctuations of amino acid residues were also measured by using the root mean square fluctuation (RMSF) metric. The RMSF of amino acids greater than 0.1 nm or 1 Å is of interest because it determines the flexible loop region for ligand binding. However, large fluctuations at the C-terminus are common and no investigation is needed [31]. Figure 9C shows the RMSF of the KRAS^G12C^ protein without drug (blue) and with drugs, including sotorasib (green), adagrasib (purple), afatinib (red), neratinib (orange), and zanubrutinib (cyan). Overall, sotorasib exhibited the lowest RMSF in most residues compared to other drugs, especially at the cysteine 12 residue, which is the important covalent binding site. However, we found that only afatinib shows an RMSF value less than 0.1 nm at the cysteine 12 residue, similar to sotorasib, which indicates stable covalent binding at cysteine 12 (Figure 9D). Overall, the molecular dynamics results suggest that afatinib could form a complex with KRAS^G12C^ well, similar to the sotorasib molecule. 

### 2.8. Summary of New Predicted FDA-Approved Drugs against KRAS Mutations

Lung adenocarcinoma is the major type of NSCLC with the highest rate of mortality [32]. The TP53, KRAS and EGFR proteins are the top three proteins most commonly mutated in lung adenocarcinoma found in cohort pan-cancer (TCGA) [33] as shown in Supplementary S1, Supplementary Figure S1, and Supplementary Table S1. The KRAS and EGFR mutations in NSCLC patients were significantly correlated to the overall survival of the patients (Supplementary Figure S1), which illustrates the important prognosis of both genes in lung adenocarcinoma.

Afatinib is a second-generation oral tyrosine kinase inhibitor (TKI) that irreversibly inhibits the ErbB/HER family of receptors, including EGFR (ErBB1) and ErbB2 [34]. Afatinib is used in metastasis lung adenocarcinoma EGFR-positive patients [12]. Afatinib is a quinazoline structure containing a 3-chloro-4-fluoroanilino group at the 4-position, a 4-dimethylamino-trans-but-2-enamido group at the 6-position, and an (S)-tetrahydrofuran-3-yloxy group at the 7-position. The Michael covalent acceptor group in the 4-dimethylamino-trans-but-2-enamido group contains the tertiary amine side chain (4-dimethylamino), which could make covalent bonding with cysteine 12 more difficult than sotorasib, and adagrasib. 

Moreover, simulation of the KRAS mutation occurs after EGFR hyperactivation, which makes patients with EGFR/KRAS positive difficult to treat [35]. Inhibiting those two proteins could therefore be beneficial for the patient’s outcome. A recent study found that afatinib had significant inhibitory effects in patient-derived KRAS^G12C^-xenografted mice and KRAS^G12C^-driven mice models [35]. An ongoing phase II clinical trial is also investigating the efficacy of afatinib and selumetinib compared to doxorubicin [NCT02450656] in advanced NSCLC patients with KRAS mutations. Based on those data, afatinib could be a repurposed drug against KRAS^G12C^ mutation. However, the primary indication of afatinib is a treatment of NSCLC, therefore clinical trials, retrospective research, systematic reviews, and meta-analyses should be conducted to investigate the potency of afatinib against the KRAS^G12C^ mutation.

Neratinib is an irreversible oral inhibitor against the human epidermal growth factor receptors EGFR (ErbB1), ErbB2 (HER2), and HER4 protein [36]. Neratinib is very effective against HER2-positive breast cancer [37]. Neratinib is a quinaline compound with a 4-dimethylamino-trans-but-2-enamido group at position 6 like afatinib, but neratinib also contains a cyano group at the 3-position, a 3-chloro-4-(2-pyridylmethoxy)anilino group at the 4-position, and an ethoxy group at the 7-position. The Michael covalent warhead in the 4-dimethylamino-trans-but-2-enamido group structure can bond with the cysteine 12 residue, similar to afatinib. However, the neratinib structure has an additional steric side chain, which is a 3-chloro-4-(2-pyridylmethoxy)anilino group. This side chain makes neratinib more rigid for occupation by KRAS^G12C^ compared with afatinib, sotorasib, and adagrasib. Despite that limitation, neratinib was found to be effective in suppressing KRAS expression in blood cancer cells [38]. That study suggests that neratinib inhibits KRAS expression via suppressing mammalian MST kinase 3 and 4 proteins [38]. In addition, a study found that neratinib in combination with entinostat could rapidly inhibit KRAS expression in melanoma cells [39]. Another study also suggests that neratinib is a multi-target kinase inhibitor that can kill NSCLC cells by indirectly suppressing KRAS^G12V^ expression via inhibiting the MST4 kinase signaling pathway [40]. Moreover, recent evident supports the potential therapeutic activity of neratinib against RAS mutant-NSCLC tumors [41]. An ongoing phase I study is investigating the safety of neratinib with everolimus or trametinib in metastasis patients with EGFR, HER2, HER3/4, or KRAS mutations [NCT03065387]. Those data suggest that neratinib could be a repurposed drug against KRAS mutation. Nevertheless, a more detailed molecular mechanism of action and ligand-protein affinity testing should be performed to confirm the interaction between neratinib and the KRAS protein. 

Zanubrutinib is a second-generation Bruton tyrosine kinase (BTK) inhibitor that was approved for patients with B cell malignancies and patients with relapsed and/or refractory mantle cell lymphoma (R/R MCL) in 2021 [42]. The zanubrutinib structure contains a Michael covalent acceptor group similar to sotorasib in that it does not have a side chain group connected to the c-terminal of the Michael warhead acrylamide group (Figure 8). Zanubrutinib also contains the diphenyl ether side chain that makes its molecular length longer than sotorasib and adagrasib, which makes it occupied outside of the allosteric site of KRAS^G12C^ protein (Figure 8). Overall, the molecular docking analysis found that zanubrutinib may have less potency compared to afatinib or neratinib. Additionally, there is no evidence that zanubrutinib can inhibit KRAS-mutant cancer cells. A recent in vitro study reported that zanubrutinib can kill resistant lung adenocarcinoma cell lines by targeting the aldo-keto reductase 1C3 (AKR1C3) and ATP-binding cassette (ABC) transporters [43]. However, the detailed mechanism of inhibition is still unknown. Furthermore, zanubrutinib was only approved in 2021; thus, there is still not much experimental data on zanubrutinib that can be used to validate for the KRAS protein. Despite that, zanubrutinib is also covalently bonded with the cysteine residue of the BTK protein [44], which could make it possible to bind covalently with the cysteine residue of the KRAS protein. Therefore, much information on zanubrutinib’s potency against KRAS^G12C^ protein is needed. Further study should evaluate the possibility of this compound in the treatment of KRAS^G12C^-mutation tumors.

**Figure 10:**
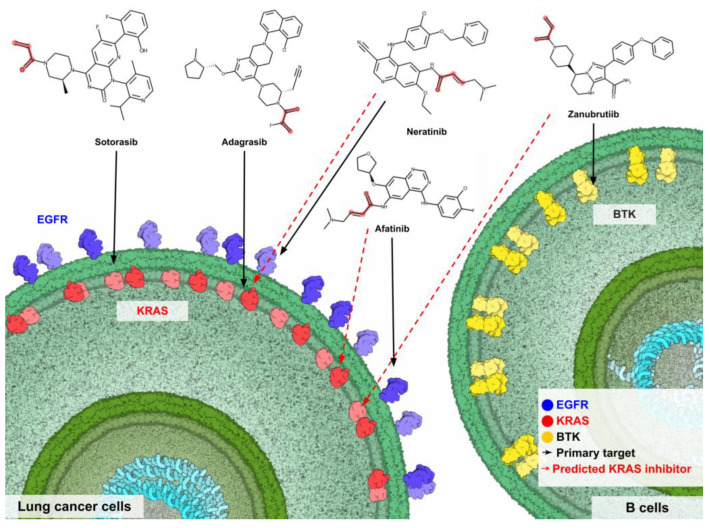
Summary of the newly predicted KRAS^G12C^ inhibitors which included afatinib, neratinib, and zanubrutinib. The EGFR, KRAS^G12C^, and BTK are represented in blue, red, and yellow protein colors. The black arrows indicate the primary targets of drugs, while the red dotted arrow indicates the newly predicted inhibitors against the KRAS^G12C^ protein. The figure was prepared by using CellPAINT software [45].

We have summarized the original and new predicted KRAS^G12C^ inhibitors found in this study in Figure 10. Afatinib, neratinib, and zanubrutinib contain no unreliable structure (outlier), i.e., the indole ring with acetamide, or azetidine. Importantly, our models passed the cross-validation, external test validation, and Y-randomization tests. Thus, the prediction of the KRAS^G21C^-inhibitory ability of these compounds is reliable. Finally, the predictive pIC_50_ values of afatinib, neratinib, and zanubrutinib were higher than 6.4, especially for afatinib, which had the highest predictive pIC_50_ value (7.43) close to sotorasib. Therefore, the FDA-approved drugs afatinib, neratinib, and zanubrutinib are recommended for further investigation against the KRAS^G12C^ protein.

## 3. Materials and Methods

### 3.1. Data Collection

All compounds with KRAS^G12C^ inhibitory concentration at 50% (IC_50_) were obtained from BindingDB [27] and ChEMBL databases [26]. Only compounds with continuous IC_50_ values were used. Redundant compounds were removed by retaining the compound with the lowest IC_50_ value. Compounds with molecular weight higher than 1000 (Da) were removed as they are less likely to be orally active molecules. The IC_50_ values were converted into molar units (M) and transformed into pIC_50_ values by the following Equation (1):

pIC_50_ = −logIC_50_ (M) (1)

The dataset for model construction contained a total of 1255 compounds consisting of training (878 compounds) and test (377 compounds) datasets. Moreover, 67 FDA-approved covalent drugs were collected from the covalent inhibitor CovalentInDB database [23]. All simplified molecular-input line-entry systems (SMILEs) and the pIC_50_ of all compounds are provided in Supplementary Tables S2 and S3. 

### 3.2. Molecular Fingerprints and Molecular Descriptors Calculation

Molecular fingerprints and molecular descriptors were calculated by using the PaDEL-descriptors [46] and Mordred descriptors [47], respectively. PubChem fingerprints (PubChemFP: 881 fingerprints) and Substructure fingerprints count (SubFPC; 307 fingerprints) were the molecular fingerprints used in this study. The Lipinski molecular descriptors (i.e., molecular weight, LogP, H-bond donor, H-bond acceptor, and the number of Lipinski violations) were used as molecular descriptors.

### 3.3. Extreme Gradient Boosting

Extreme gradient boosting (XGBoost) is a tree-boosting algorithm that was used in both classification and regression models. This algorithm can boost its performance through an ensemble method (additive strategy) that includes decision-tree models trained in sequence [48]. For a given molecule with n samples and m descriptors, let $D=\left\{{(x}_{i}{,\;y}_{i})\right\}\;\left(\left|D\right|{=n,\;x}_{i}\;\in \;{\mathbb{R}}^{m}{,\;y}_{i}\in \;\mathbb{R}\right),$ then a tree ensemble model using *K* additive function to predict the output can be expressed as showed in Equation (2):

(2) $$\hat{y_{i}}=\emptyset {(x}_{i})=\overset{K}{\underset{k=1}{\sum }}f_{k}{(x}_{i}{),\;f}_{k}\;\in \;F$$

where $F=\;\{\;f_{k}{(x}_{i}{)=w_{q}(x}_{i})\}\;(q:\;{\mathbb{R}}^{m}\;\to \;T,\;w\;\in \;{\mathbb{R}}^{T})$ is the space of regression tree in an XGBoost. The$\;f_{k}$ function at each of *K* steps maps the molecular descriptor values in $x_{i}$ to the certain output ($y_{i}$). The q represents the structure of each tree, *T* is the number of leaves in the tree, and the $w_{q}$ is the leaf weight. The final prediction ($\hat{y_{i}}$) of the XGBoost model is the sum of all$\;f_{k}{(x}_{i})\;$decision-tree predictions. In addition, the XGBoost algorithm also minimizes the regularized objective by calculating the penalization term and loss function as shown in Equation (3).

(3) $$\begin{array}{c} L(\emptyset )=\underset{i}{\sum }{l(y}_{i},\hat{y_{i}})+\underset{k}{\sum }{\Omega (f}_{k})\;\\ \mathrm{where}\;{\Omega (f}_{k})=\gamma T+\frac{1}{2}{\lambda \|w\|}^{2} \end{array}$$

Here, l is a differentiable convex loss function that measures the difference between the prediction $\hat{y_{i}}$ and the target output${\;y}_{i}$. The second term Ω penalizes the complexity of the model, which is known as the regularization term. The regularization term contains *γ* and *λ* parameters that helps to smooth the final learnt weights to avoid overfitting.

Moreover, XGBoost iteratively trains a new decision tree based on the output of the previous tree. Formally, let ${\hat{y_{i}}}^{\left(t\right)}\;$be the prediction of the *t*-th iteration, the $f_{t}{(x}_{i})$ needs to be added to minimize the objective function as shown in Equation (4).

(4) $$L^{\left(t\right)}(\emptyset )=\overset{n}{\underset{i=1}{\sum }}{l(y}_{i},{\hat{y_{i}}}^{\left(t-1\right)}{+f}_{t}{(x}_{i}{))+\Omega (f}_{t})\;$$

The XGBoost algorithm introduces the first and second derivatives of this objective function, which can be expressed as shown in Equation (5):

(5) $$L^{\left(t\right)}(\emptyset )\;≃\;\overset{n}{\underset{i=1}{\sum }}\left[{l(y}_{i},{\hat{y_{i}}}^{\left(t-1\right)}{)+g}_{i}f_{t}{(x}_{i})+\frac{1}{2}{\;h}_{i}f_{t}{}^{2}{(x}_{i})\right]{+\Omega (f}_{t})\;$$

where $g_{i}=\partial_{{\hat{y_{i}}}^{\left(t-1\right)}}{l(y}_{i},{\hat{y_{i}}}^{\left(t-1\right)})\;$and $h_{i}=\partial^{2}{}_{{\;\hat{y_{i}}}^{\left(t-1\right)}}{l(y}_{i},{\hat{y_{i}}}^{\left(t-1\right)})$ are first and second order gradient statistics on the loss function, respectively. Define $I_{j}{=\{i|q(x}_{i})=j\}\;$as the instance set of leaf *j*, then the constant terms can be removed and the Ω term can be expanded to obtain the following simplified objective at step *t* as shown in Equation (6).

(6) $${\tilde{L}}^{\left(t\right)}(\emptyset )=\overset{n}{\underset{i=1}{\sum }}\left[{\;g}_{i}f_{t}{(x}_{i})+\frac{1}{2}{\;h}_{i}f_{t}{}^{2}{(x}_{i})\right]+\gamma T+\frac{1}{2}\lambda \overset{T}{\underset{j=1}{\sum }}w_{j}{}^{2}\;$$

A total of seven parameters were fine-tuned with grid-search cross validation as described in Section 3.4 and Section 3.6. Default values were used for other parameters.

### 3.4. Classification Model Construction

The classification model was constructed based on the conjoint fingerprint of PubChem fingerprint and Substructure fingerprint count with the XGBoost machine learning model by using XGBoost software [48]. The classification model fine-tuned hyperparameters by using grid-search 5-fold cross-validation (CV). The hyperparameters of the XGBoost model were base_score = 0.5, n_estimators (n) = 100, max_depth = 4, gamma (γ) = 0, reg_alpha = 0, reg_lambda (λ) = 0.5, and learning_rate (*t*) = 0.3. The classification model was internally validated by using the 5-fold CV and externally validated by using an external test dataset. The evaluation metrics are described in Section 3.5. 

### 3.5. Classification Model Evaluation

The classification model was evaluated by using accuracy, and area under the curve of the receiver operating characteristic plot (AUC), metrics. The accuracy metric represents the model’s accuracy for predicting both classes, while the AUC indicates the rate of correct prediction for the active group. The accuracy score was calculated by Equation (7).

(7) $$\mathrm{Accuracy}=\frac{\left(\mathrm{TP}+\mathrm{TN}\right)}{\left(\mathrm{TP}+\mathrm{TN}+\mathrm{FP}+\mathrm{FN}\right)}$$

The TP, TN, FP, and FN are the true positive, true negative, false positive, and false negative values, respectively. The AUC metric was calculated by using the area under the curve between the true positive rate (TPR), and false positive rate (FPR) as shown in Equations (8) and (9).

(8) $$\mathrm{TPR}=\frac{\mathrm{TP}}{\left(\mathrm{TP}+\mathrm{FN}\right)}$$

(9) $$\mathrm{FPR}=\frac{\mathrm{FP}}{\left(\mathrm{FP}+\mathrm{TN}\right)}$$

Both accuracy and AUC metrics were calculated for the training dataset (Accuracy_Tr_ and AUC_Tr_), 5-fold CV (Accuracy_CV_ and AUC_cv_), and the test dataset (Accuracy_Ext_ and AUC_Ext_).

### 3.6. Regression Model Construction

The regression model was constructed based on the conjoint fingerprint of PubChem fingerprint and Substructure fingerprint count (a total of 1188 fingerprints) with the XGBoost algorithm [48]. The regression model fine-tuned hyperparameters by using grid-search 5-fold cross-validation (CV). The hyperparameters of the XGBoost model were base_score = 0.5, n_estimators (n) = 100, max_depth = 4, gamma (γ) = 0, reg_alpha = 0.1, reg_lambda (λ) = 0.5, and learning_rate (*t*) = 0.3. The classification model was internally validated by using the 5-fold CV and externally validated by using an external test dataset. The regression model was evaluated by using the method in Section 3.7. 

### 3.7. Regression Model Evaluation

The regression model was evaluated by the mean absolute error (MAE) and the coefficient of determination (R^2^ for the training dataset or Q^2^ for the validation dataset). The MAE and R^2^ or Q^2^ were calculated by using Equations (10) and (11).

(10) $$\mathrm{MAE}=\mathrm{Average}\;(\left|y_{i}-\hat{y_{i}}\right|)$$

(11) $$R^{2}{\;\mathrm{or}\;Q}^{2}=1-\;\frac{\sum_{i=1}^{n}{{(y}_{i}-\hat{y_{i}})}^{2}}{\sum_{i=1}^{n}{{(y}_{i}-\overset{-}{y_{i}})}^{2}}$$

The $y_{i}$ is an experimental pIC_50_ value, the $\hat{y_{i}}$ is the corresponding predictive pIC_50_ value of the $y_{i}$ value, while the $\overset{-}{y_{i}}$ is the average of experimental pIC_50_ values. The n value is the total number of compounds in the dataset. The MAE value indicates the prediction error of the model. MAE values between 0 and 1 are required for good prediction accuracy (less prediction error). The coefficient of determination (R^2^ or Q^2^) determines the percentage of variance from the experimental pIC_50_ values that can be captured by the model. An R^2^ or Q^2^ near 1 indicates that the model well-explained the variance of y by x. Both MAE and R^2^ or Q^2^ metrics were calculated for the training dataset (MAE_Tr_ and R^2^_Tr_), 5-fold CV (MAE_CV_ and Q^2^_cv_), and the test dataset (MAE_Ext_ and Q^2^_Ext_).

### 3.8. Y-Randomization

The regression model’s robustness and stability were validated using the Y-randomization model (*n* = 100). The Y-randomization experiment can test the reliability of the regression models. In this step, the x–y pairs of the training set were shuffled to generate false x–y pairs. The false x–y pairs were used to train the model and generate the R^2^ and Q^2^ values of the x–y false pairs. The model was determined to be robust if the Y-randomized models performed poorly in comparison to their original x-y pairs. If the Y-randomized models provided similar predictive performance to the original x–y pairs, the model was deemed to be unreliable. 

### 3.9. Feature Importance

The molecular fingerprints that correlated with the output of the models were identified by using Shapley Additive explanations (SHAP) feature importance value. The SHAP values were used to examine the influence of the individual fingerprint on the model’s outcome. SHAP assigns a value to each molecular fingerprint based on the impact of the model output. These values are calculated for each compound separately (local explanation), and then the SHAP values were built-up to explain the global effect of each molecular fingerprint on the model [28]. High SHAP values indicate high feature importance, whereas low SHAP values indicate low feature importance.

### 3.10. Applicability Domain

The applicability domain must be tested to explore the application of the machine learning model [29]. Since the model can be expected to have a reliable prediction for chemicals similar to those used in the model construction, the applicability domain should be tested to identify outliers or unreliable chemical compounds. Standardized residuals of model prediction and the predictive pIC_50_ was plotted to assess the applicability domain and molecular similarity, respectively. The standardized residuals were calculated by using Equation (12):

(12) $$\mathrm{Standardized}\;\mathrm{residual}=\frac{\left(\left|y_{i}-\hat{y_{i}}\right|\;-\mathrm{Average}\;(\left|y_{i}-\hat{y_{i}}\right|\right)}{\mathrm{Standard}\;\mathrm{deviation}\;(\left|y_{i}-\hat{y_{i}}\right|)}$$

The $y_{i}$ is an experimental pIC_50_ value, while the $\hat{y_{i}}$ is the corresponding predictive pIC_50_ value of the y-value. A standard residual higher than 3 is considered an outlier of the model.

### 3.11. Covalent Docking

The covalent docking was performed using SeeSAR software (BioSolveIT, Sankt Augustin, Germany). The covalent warhead functional groups were manually marked based on the position provided by the CovalentInDB [23]. The KRAS active site was defined as the cysteine 12 residue of the KRAS^G12C^ protein (PDB ID: 6OIM). All predicted active class compounds were used to evaluate their binding mode in the KRAS^G12C^ protein. The protein was curated by removing water, adding hydrogen atoms, repairing all missing atoms in the amino acid residues, and calculating the charges by using the MGL tool. The energy minimization of the universal force field (UFF) was utilized for all chemicals before performing the molecular docking. The ligand–protein interaction was evaluated by PyMoL software (Schrödinger, Inc., New York, NY, USA).

### 3.12. Molecular Dynamic Simulation

The molecular dynamics simulation was done by using the GROMACS software [49] with the CHARMM36 force field [50]. The KRAS^G12C^ protein (PDB: 6OIM) was used in the molecular dynamics simulation. The ligand–protein complex was solvated within a cubic box of the transferable intermolecular potential with a three-points (TIP3P) water model (100 × 100 × 100 Å). The CHARMM force field parameters for the ligands were generated using the CHARMM General Force Field (CGenFF) program (https://cgenff.umaryland.edu/ accessed on 15 November 2022) [51]. The protein residues were assigned to their standard ionization states at physiological conditions (pH 7.0), and the whole complexes were neutralized with sufficient numbers of Na+ and Cl− ions added via the Monte Carlo ion-placing method. The MD simulation was conducted for 20 ns. The RMSD, RMSD of ligand, and RMSF were analyzed by using the GROMACS built-in tools. 

### 3.13. Statistical Analysis

The principal component analysis (PCA) was performed by using Scikit-learn library in Python software (Python Software Foundation, Fredericksburg, VA, USA). The molecular fingerprints were normalized by using max–min normalization before conducting the PCA analysis with a 0.95 explain variance threshold. All software was written and visualized using Python 3.9 software and matplotlib library (Python Software Foundation, Fredericksburg, VA, USA), respectively.

## 4. Conclusions

In this work, we describe how to use machine learning, molecular docking, and molecular dynamics tools for drug repurposing. The machine learning models were built by using PubChem fingerprint and Substructure fingerprint count. The extreme gradient boost (XGBoost) algorithm was used to build the models. These models provide fast-running time, high accuracy, and low prediction error for both classification and regression predictions. The drug repurposing was conducted by predicting new inhibitors from the 67 FDA-approved covalent drugs reported in the CovalentInDB. Only afatinib, dacomitinib, acalabrutinib, neratinib, zanubrutinib, dutasteride, and finasteride were predicted to be active, however, when covalent docking was assessed, only afatinib, neratinib, and zanubrutinib were found to be active. Thus, the new inhibitors identified by our model are afatinib, neratinib, and zanubrutinib. Future research is needed to confirm the KRAS^G12C^-inhibitory activity of the predicted compounds in both in vitro and in vivo experiments before conducting any clinical evaluation.

## Figures and Tables

**Table 1:** List of similar importance molecular fingerprints extracted from classification and regression models using the SHAP algorithm.

Molecular Fingerprints	Descriptions	Examples ^1^
PubChemFP378	C(~N)(:C)(:N)	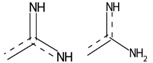
SubFPC274	Aromatic atom	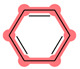
SubFPC307	Chiral center	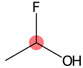
SubFPC171	Aryl Chloride	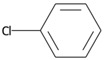
SubFPC1	Primary carbon	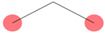
SubFPC295	C-O, N, or S bond	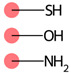
PubChemFP261	≥4 aromatic rings	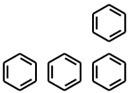
PubChemFP192	≥3 any ring size 6	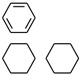
SubFPC300	1,3-tautomerizable	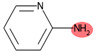
SubFPC2	Secondary carbon	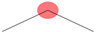

^1^ The red highlight is the example of the substructure molecular fingerprint.

**Table 2:** Predicted FDA approved drugs against KRAS^G12C^ protein.

Name	Predicted Classes	Predicted pIC_50_	Experimental pIC_50_^†^	Primary Targets [30]	Primary Indications [30]
Afatinib	Active	7.43	No report	EGFR and HER2	NSCLC
Dacomitinib	Active	7.32	No report	EGFR T790M	NSCLC
Acalabrutinib	Active	6.75	No report	BTK	MCL
Neratinib	Active	6.68	No report	EGFR, HER2, HER4	Breast cancer
Zanubrutinib	Active	6.49	No report	BTK	MCL
Dutasteride	Active	5.94	No report	5α-Reductase	BPH
Finasteride	Active	5.05	No report	5α-Reductase	Alopecia, BPH
Sotorasib *	Active	6.95	7.52	KRAS^G12C^	NSCLC
Adagrasib *	Active	8.02	8.30	KRAS^G12C^	NSCLC

* FDA-approved inhibitors for KRAS^G12C^ protein. ^†^ Experimental pIC_50_ obtained from ChEMBL and BindingDB.

## Data Availability

All chemical compounds with pIC_50_ values are available in the Supplementary Materials.

## Supplementary Materials

The supporting information can be downloaded at: https://www.mdpi.com/article/10.3390/ijms24010669/s1.
