# Supplementary material for: Drug Repurposing against KRAS Mutant G12C: A Machine Learning, Molecular Docking, and Molecular Dynamics Study
Source: Int J Mol Sci. 2022 Dec 30;24(1):669. doi: 10.3390/ijms24010669 (PMC9821013; doi:10.3390/ijms24010669)
Supplement: Supplementary file 1 [file ijms-24-00669-s001.zip › ijms-2097117-supplementary.pdf]

Supplementary materials

Drugs Repurposing against KRAS Mutant G12C: A Machine Learning, Molecular Docking, and Molecular Dynamics Study

Tarapong Srisongkram *, Natthida Weerapreeyakul

Division of Pharmaceutical Chemistry, Faculty of Pharmaceutical Sciences, Khon Kaen University, Khon Kaen 40002, Thailand; natthida@kku.ac.th

* **Correspondence: tarasri@kku.ac.th**

Supplementary S1. KRAS-associated mutant genes in non-small cell lung cancer (lung adenocarcinoma)

The top 10% mutated genes of lung adenocarcinoma found in cohort pan-cancer (TCGA) were analyzed as shown in Figure 1. The result demonstrates that TP53 (274 cases; 53%), KRAS (134 cases; 26%), FAT4 (97 cases; 19%), EGFR (76 cases; 15%), and SETBP1 (66 cases; 13%) are the top 5 frequent genes highly found in lung adenocarcinoma patients (n=513) (Supplementary Figure 1A, red). The number of mutations in each top 5 genes varied from 14 to 205 consequences. For example, TP53 contains 205 mutations, KRAS contains 14 mutations, FAT4 contains 119 mutations, EGFR contains 44 mutations, and SETBP1 contains 79 mutations (Supplementary Figure 1A, blue). It should be noted that KRAS–the second most mutated gene has only 14 mutation types compared to other genes.

Besides, the $KRAS^{G12C}$ was the highest mutation accounting for 50/134 cases or 37% of total KRAS mutations in the cohort (Supplementary Figure 1B, G12C), while the EGFR L858R was the highest mutation accounting for 21/76 cases or 28% of total EGFR mutations in the cohort (Supplementary Figure 1C, L858R). Moreover, the KRAS and EGFR mutations in NSCLC patients (n=513) were significantly correlated to the overall survival of the patients with a hazard ratio (HR) at 1.56 (95%CI: 1.13-2.15, $p < 0.05$), and 1.49 (95%CI: 1.09-2.04, $p < 0.05$) (Supplementary Figure 1D and 1E), respectively. On the other hand, the TP53 non-significantly showed the correlation between gene expression and overall survival of the patients (HR = 0.85 with the 95%CI: 0.61-1.17, p =0.031) (Supplementary Figure 1F). The significant correlation between the gene expression of KRAS and EGFR and the dead/alive of patients illustrates the important prognosis of both genes in lung adenocarcinoma.

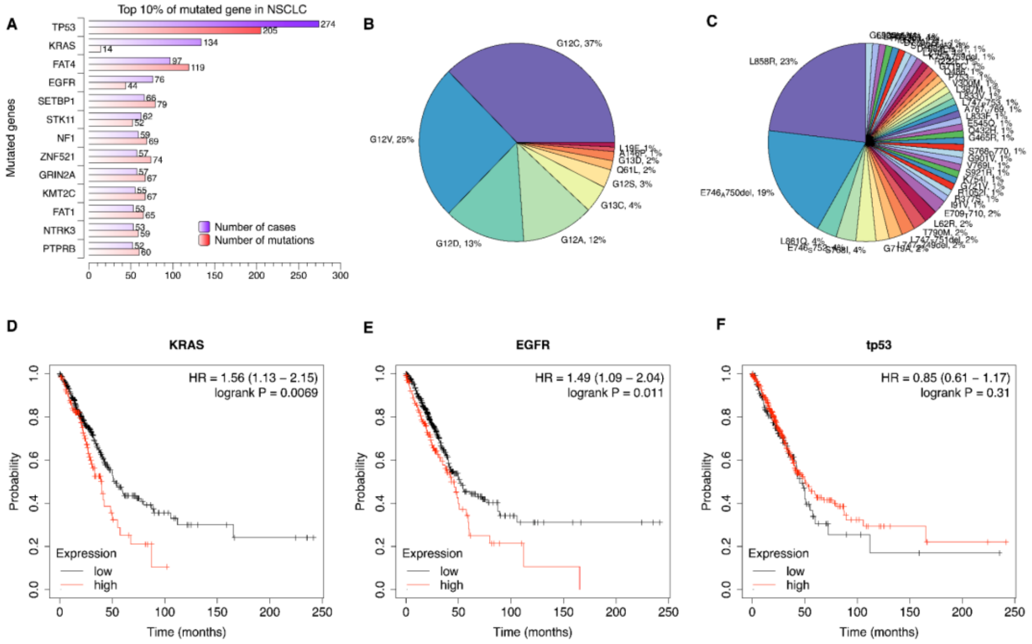

Supplementary Figure S1. Overall mutations of NSCLC patients. (**A**) The total number of cases (purple) and a total number of mutated codons (red) for each gene in the top 10% of mutated genes in lung adenocarcinoma patients. (**B**) Distribution of mutation in KRAS gene. (**C**) Distribution of mutation in EGFR gene. (**D**) Kaplan-Meier analysis of KRAS expression. (**E**) Kaplan-Meier analysis of EGFR expression. (**F**) Kaplan-Meier analysis of TP53 expression. Red and black lines indicate a high and low expression of each gene (n = 513).

Supplementary Table S1 Distribution of gene mutation in EGFR cases (total = 76 cases)

EGFR	AffectedCase
L858R	21
E746_A750del	17
L861Q	3
E746_S752	3
S768I	3
G719A	2
L747_E749del	2
L747_T751del	2
T790M	2
L62R	2
E709_T710	2
I91V	1
R377S	1
R1052I	1

G721V	1
K754I	1
S921R	1
V769L	1
G901V	1
S768_D770	1
G465R	1
Q432H	1
E545Q	1
L833F	1
A767_V769	1
L747_P753	1
L833V	1
L387M	1
V300M	1
P753=	1
Q486	1
G719C	1
R222L	1
K754_I759del	1
L210=	1
D1083Efs*11	1
S752Pfs*3	1
D770_N771	1
I853=	1
H773du	1
L747_T751	1
L907M	1
G652=	1

Supplementary Table S2: Compounds used in the model construction

LigandID	name	pIC50
CHEMBL4855757	Oc1cc(-c2ncc3c(N4CC5CCC(C4)N5)nc(OCC45CCCN4C(CF)CC5)nc3c2F)c2ccccc2c1	10
BDBM50579603	Oc1cc(-c2ncc3c(nc(OCC45CCCN4C(CF)CC5)nc3c2F)N2CC3CCC(C2)N3)c2ccccc2c1	10
BDBM50579600	Oc1cc(-c2ncc3c(nc(OC[C@@]45CCCN4C[C@H](F)C5)nc3c2F)N2CC3CCC(C2)N3)c2c(Cl)cccc2c1	9.7
CHEMBL4857438	Oc1cc(-c2ncc3c(N4CC5CCC(C4)N5)nc(OC[C@@]45CCCN4C[C@H](F)C5)nc3c2F)c2c(Cl)cccc2c1	9.7
CHEMBL4863339	C#Cc1cccc2cc(O)cc(-c3ncc4c(N5CC6CCC(C5)N6)nc(OCC56CCCN5CCC6)nc4c3F)c12	9.52
BDBM50579595	Oc1cc(-c2ncc3c(nc(OCC45CCCN4CCC5)nc3c2F)N2CC3CCC(C2)N3)c2c(cccc2c1)C#C	9.52

BDBM50579597	Oc1cc(-c2ncc3c(nc(OC[C@@]45CCCN4C[C@H](F)C5)nc3c2F)N2CC3CCC(C2)N3)c2c(cccc2c1)C#C	9.4
BDBM50579594	Oc1cc(-c2ncc3c(nc(OCC45CCCN4CCC5)nc3c2F)N2CC3CCC(C2)N3)c2c(Cl)cccc2c1	9.4
CHEMBL4859236	Oc1cc(-c2ncc3c(N4CC5CCC(C4)N5)nc(OCC45CCCN4CCC5)nc3c2F)c2c(Cl)cccc2c1	9.4
BDBM50579601	Oc1cc(-c2ncc3c(nc(OC[C@@]45CCCN4C[C@H](F)C5)nc3c2F)N2CC3CCC(C2)N3)c2c(C#C)c(F)ccc2c1	9.4
CHEMBL4858364	C#Cc1c(F)ccc2cc(O)cc(-c3ncc4c(N5CC6CCC(C5)N6)nc(OC[C@@]56CCCN5C[C@H](F)C6)nc4c3F)c12	9.4
CHEMBL4876040	C#Cc1cccc2cc(O)cc(-c3ncc4c(N5CC6CCC(C5)N6)nc(OC[C@@]56CCCN5C[C@H](F)C6)nc4c3F)c12	9.4
CHEMBL4857719	Oc1cc(-c2ncc3c(N4CC5CCC(C4)N5)nc(OCC45CCCN4CCC5)nc3c2F)c2c(Cl)c(F)ccc2c1	9.3
BDBM50579598	Oc1cc(-c2ncc3c(nc(OCC45CCCN4CCC5)nc3c2F)N2CC3CCC(C2)N3)c2c(Cl)c(F)ccc2c1	9.3
BDBM50572114	CC(C)c1ncnc2CCCOc3ccc(F)c(F)c3-c3nc4n(-c12)c(=O)nc(N1C[C@@H](C)N(C[C@@H]1C)C(=O)C=C)c4cc3Cl	9.29
CHEMBL4855521	[2H]C1([2H])Oc2ccc(F)c(F)c2-c2nc3c(cc2Cl)c(N2C[C@@H](C)N(C(=O)C=C)C[C@@H]2C)nc(=O)n3-c2c(ccnc2C(C)C)OC1([2H])[2H]	9.29
BDBM50572112	[2H]C1([2H])Oc2ccc(F)c(F)c2-c2nc3n(-c4c(OC1([2H])[2H])ccnc4C(C)C)c(=O)nc(N1C[C@@H](C)N(C[C@@H]1C)C(=O)C=C)c3cc2Cl	9.29
CHEMBL4852458	C=CC(=O)N1C[C@H](C)N(c2nc(=O)n3c4nc(c(Cl)cc24)-c2c(ccc(F)c2F)OCCCc2ncnc(C(C)C)c2-3)C[C@H]1C	9.29
BDBM50572118	CC(C)c1nccc2CCC(F)(F)COc3cccc(F)c3-c3nc4n(-c12)c(=O)nc(N1CCN(C[C@@H]1C)C(=O)C=C)c4cc3Cl	9.25
CHEMBL4856277	C=CC(=O)N1CCN(c2nc(=O)n3c4nc(c(Cl)cc24)-c2c(F)cccc2OCC(F)(F)CCc2ccnc(C(C)C)c2-3)[C@@H](C)C1	9.25
CHEMBL4867851	C#Cc1c(F)ccc2cc(O)cc(-c3ncc4c(N5CC6CCC(C5)N6)nc(OCC56CCCN5CCC6)nc4c3F)c12	9.22
CHEMBL4876243	COC1CN2CCCC2(COc2nc(N3CC4CCC(C3)N4)c3cnc(-c4cc(O)cc5cccc(F)c45)c(F)c3n2)C1	9.22
CHEMBL4874297	Oc1cc(-c2ncc3c(N4CC5CCC(C4)N5)nc(OC[C@@]45CCCN4C[C@H](F)C5)nc3c2F)c2c(F)c(F)ccc2c1	9.22
BDBM50579599	Oc1cc(-c2ncc3c(nc(OC[C@@]45CCCN4C[C@H](F)C5)nc3c2F)N2CC3CCC(C2)N3)c2c(F)c(F)ccc2c1	9.22
BDBM50579604	COC1CN2CCCC2(COc2nc(N3CC4CCC(C3)N4)c3cnc(c(F)c3n2)-c2cc(O)cc3cccc(F)c23)C1	9.22
BDBM50579596	Oc1cc(-c2ncc3c(nc(OCC45CCCN4CCC5)nc3c2F)N2CC3CCC(C2)N3)c2c(C#C)c(F)ccc2c1	9.22
BDBM50576728	CN(C)CC1(COc2nc3CN(CCc3c(n2)N2CC3CCC(C2)N3)c2cc(O)cc3cccc(I)c23)CC1	9.15
BDBM50576730	Oc1cc(N2CCc3c(C2)nc(OCC2(CN4CCOCC4)CC2)nc3N2CC3CCC(C2)N3)c2c(I)cccc2c1	9.15
CHEMBL4849611	CN(C)CC1(COc2nc3c(c(N4CC5CCC(C4)N5)n2)CCN(c2cc(O)cc4cccc(I)c24)C3)CC1	9.15
CHEMBL4869200	Oc1cc(N2CCc3c(nc(OCC4(CN5CCOCC5)CC4)nc3N3CC4CCC(C3)N4)C2)c2c(I)cccc2c1	9.15
BDBM50579602	CCc1c(F)ccc2cc(O)cc(-c3ncc4c(nc(OC[C@@]56CCCN5C[C@H](F)C6)nc4c3F)N3CC4CCC(C3)N4)c12	9.1
CHEMBL4863371	CCc1c(F)ccc2cc(O)cc(-c3ncc4c(N5CC6CCC(C5)N6)nc(OC[C@@]56CCCN5C[C@H](F)C6)nc4c3F)c12	9.1
CHEMBL4876274	Oc1cc(N2CCc3c(nc(OCC4(CN5CC[C@@H](F)C5)CC4)nc3N3CC4CCC(C3)N4)C2)c2c(I)cccc2c1	9.05
BDBM50567176	C[C@H](CN(C)C)NC(=O)c1cc(N2CCN([C@@H](CC#N)C2)C(=O)C=C)c2CCN(Cc2n1)c1c(Cl)c(C)cc2[nH]ncc12	9.05
BDBM50576729	Oc1cc(N2CCc3c(C2)nc(OCC2(CN4CC[C@@H](F)C4)CC2)nc3N2CC3CCC(C2)N3)c2c(I)cccc2c1	9.05
CHEMBL4856826	C=CC(=O)N1CCN(c2cc(C(=O)N[C@H](C)CN(C)C)nc3c2CCN(c2c(Cl)c(C)cc4[nH]ncc24)C3)C[C@@H]1CC#N	9.05
BDBM50572113	CC(C)c1nccc2OCC(F)(F)COc3cccc(F)c3-c3nc4n(-c12)c(=O)nc(N1C[C@@H](C)N(C[C@@H]1C)C(=O)C=C)c4cc3Cl	9.04
CHEMBL4879271	C=CC(=O)N1C[C@H](C)N(c2nc(=O)n3c4nc(c(Cl)cc24)-c2c(F)cccc2OCC(F)(F)COc2ccnc(C(C)C)c2-3)C[C@H]1C	9.04
BDBM50567170	CN(C)[C@@H]1CCC[C@H]1NC(=O)c1cc(N2CCN([C@@H](CC#N)C2)C(=O)C=C)c2CCN(Cc2n1)c1cccc2cccc(C)c12	9
CHEMBL4874744	C=CC(=O)N1CCN(c2cc(C(=O)N[C@@H]3CCC[C@H]3N(C)C)nc3c2CCN(c2cccc4cccc(C)c24)C3)C[C@@H]1CC#N	9
CHEMBL4632935	C=CC(=O)N1CCN(c2nc(OC[C@@H]3CCCN3C)nc3c2CCN(c2cccc4cccc(C)c24)C3)C[C@@H]1CC#N	9
BDBM50539761	CN1CCC[C@H]1COc1nc2CN(CCc2c(n1)N1CCN([C@@H](CC#N)C1)C(=O)C=C)c1cccc2cccc(Cl)c12	9
BDBM50539762	CN1CCC[C@H]1COc1nc2CN(CCc2c(n1)N1CCN([C@@H](CC#N)C1)C(=O)C=C)c1cccc2cccc(C)c12	9
CHEMBL4648852	C=CC(=O)N1CCN(c2nc(OC[C@@H]3CCCN3C)nc3c2CCN(c2cccc4cccc(Cl)c24)C3)C[C@@H]1CC#N	9
BDBM50572111	CC(C)c1nccc2OCCOc3c(F)ccc(F)c3-c3nc4n(-c12)c(=O)nc(N1C[C@@H](C)N(C[C@@H]1C)C(=O)C=C)c4cc3Cl	8.96
CHEMBL4875422	C=CC(=O)N1C[C@H](C)N(c2nc(=O)n3c4nc(c(Cl)cc24)-c2c(F)ccc(F)c2OCCOc2ccnc(C(C)C)c2-3)C[C@H]1C	8.96
BDBM50576726	Oc1cc(N2CCc3c(C2)nc(OCC2(CN4CCOCC4)CC2)nc3N2CC3CCC(C2)N3)c2c(Br)cccc2c1	8.92

CHEMBL4872714	Oc1cc(N2CCc3c(nc(OCC4(CN5CCOCC5)CC4)nc3N3CC4CCC(C3)N4)C2)c2c(Br)cccc2c1	8.92
CHEMBL4863593	C=CC(=O)N1C[C@H](C)N(c2nc(=O)n3c4nc(c(F)cc24)-c2c(ccc(F)c2F)OCCC(C)c2ccnc(C(C)C)c2-3)C[C@H]1C	8.85
BDBM50572117	CC(C)c1nccc2C(C)CCOc3ccc(F)c(F)c3-c3nc4n(-c12)c(=O)nc(N1C[C@@H](C)N(C[C@@H]1C)C(=O)C=C)c4cc3F	8.85
CHEMBL4875572	Oc1cc(N2CCc3c(nc(OCC4(CN5CC[C@@H](F)C5)CC4)nc3N3CC4CCC(C3)N4)C2)c2c(Br)cccc2c1	8.82
BDBM50576725	Oc1cc(N2CCc3c(C2)nc(OCC2(CN4CC[C@@H](F)C4)CC2)nc3N2CC3CCC(C2)N3)c2c(Br)cccc2c1	8.82
BDBM50579589	CC(C)c1nccc(C)c1-n1c2nc(c(Cl)cc2c(N2CCN(CC2)C(=O)C=C)c(C#N)c1=O)-c1c(N)c(Cl)cc(Cl)c1F	8.82
CHEMBL4859820	C=CC(=O)N1CCN(c2c(C#N)c(=O)n(-c3c(C)ccnc3C(C)C)c3nc(-c4c(N)c(Cl)cc(Cl)c4F)c(Cl)cc23)CC1	8.81
CHEMBL4864188	C=CC(=O)N1C[C@H](C)N(c2nc(=O)n3c4nc(c(F)cc24)-c2c(F)cccc2OCCCc2ccnc(C(C)C)c2-3)C[C@H]1C	8.77
BDBM50572115	CC(C)c1nccc2CCCOc3cccc(F)c3-c3nc4n(-c12)c(=O)nc(N1C[C@@H](C)N(C[C@@H]1C)C(=O)C=C)c4cc3F	8.77
CHEMBL4850201	C=CC(=O)N1CCN(c2cc(C(=O)N[C@H](C)CN3CC(C)(O)C3)nc3c2CCN(c2cccc4cccc(C)c24)C3)C[C@@H]1CC#N	8.66
BDBM50567171	C[C@H](CN1CC(C)(O)C1)NC(=O)c1cc(N2CCN([C@@H](CC#N)C2)C(=O)C=C)c2CCN(Cc2n1)c1cccc2cccc(C)c12	8.66
CHEMBL4855378	C=CC(=O)N1C[C@H](C)N(c2nc(=O)n3c4nc(c(F)cc24)-c2c(Cl)cccc2OCCCc2ccnc(C(C)C)c2-3)C[C@H]1C	8.64
BDBM50572116	CC(C)c1nccc2CCCOc3cccc(Cl)c3-c3nc4n(-c12)c(=O)nc(N1C[C@@H](C)N(C[C@@H]1C)C(=O)C=C)c4cc3F	8.64
BDBM50572108	CC(C)c1nccc2CCCCOc3cccc(F)c3-c3nc4n(-c12)c(=O)nc(N1CCN(C[C@@H]1C)C(=O)C=C)c4cc3F	8.62
CHEMBL4869082	C=CC(=O)N1CCN(c2nc(=O)n3c4nc(c(F)cc24)-c2c(F)cccc2OCCCCc2ccnc(C(C)C)c2-3)[C@@H](C)C1	8.62
BDBM50579588	CC(C)c1nccc(C)c1-n1c2nc(c(Cl)cc2c(N2CCN([C@H](C)C2)C(=O)C=C)c(C#N)c1=O)-c1c(N)c(Cl)cc(Cl)c1F	8.6
CHEMBL4870767	C=CC(=O)N1CCN(c2c(C#N)c(=O)n(-c3c(C)ccnc3C(C)C)c3nc(-c4c(N)c(Cl)cc(Cl)c4F)c(Cl)cc23)C[C@H]1C	8.6
BDBM50572119	CC(C)c1nccc2CCOc3cccc(F)c3-c3nc4n(-c12)c(=O)nc(N1C[C@@H](C)N(C[C@@H]1C)C(=O)C=C)c4cc3Cl	8.59
CHEMBL4876119	C=CC(=O)N1C[C@H](C)N(c2nc(=O)n3c4nc(c(Cl)cc24)-c2c(F)cccc2OCCc2ccnc(C(C)C)c2-3)C[C@H]1C	8.59
CHEMBL4860030	C=CC(=O)N1CCN(c2c(C#N)c(=O)n(-c3c(C(C)C)ncnc3C(C)C)c3nc(-c4c(N)cccc4F)c(Cl)cc23)CC1	8.56
BDBM50579593	CC(C)c1ncnc(C(C)C)c1-n1c2nc(c(Cl)cc2c(N2CCN(CC2)C(=O)C=C)c(C#N)c1=O)-c1c(N)cccc1F	8.55
BDBM50579587	CC(C)c1nccc(C)c1-n1c2nc(c(Cl)cc2c(N2C[C@H](C)N([C@H](C)C2)C(=O)C=C)c(C#N)c1=O)-c1c(N)c(Cl)cc(Cl)c1F	8.55
CHEMBL4873282	C=CC(=O)N1[C@@H](C)CN(c2c(C#N)c(=O)n(-c3c(C)ccnc3C(C)C)c3nc(-c4c(N)c(Cl)cc(Cl)c4F)c(Cl)cc23)C[C@H]1C	8.55
CHEMBL4640636	CN1CCC[C@H]1COc1nc2c(c(N3CCN(C(=O)/C=C/CF)[C@@H](CC#N)C3)n1)CCN(c1cccc3cccc(Cl)c13)C2	8.52
BDBM50539765	CN1CCC[C@H]1COc1nc2CN(CCc2c(n1)N1CCN([C@@H](CC#N)C1)C(=O)\C=C\CF)c1cccc2cccc(Cl)c12	8.52
CHEMBL4857517	Cc1cccc2cc(O)cc(N3CCc4c(nc(OCC5(CN6CCOCC6)CC5)nc4N4CC5CCC(C4)N5)C3)c12	8.49
BDBM50576727	Cc1cccc2cc(O)cc(N3CCc4c(C3)nc(OCC3(CN5CCOCC5)CC3)nc4N3CC4CCC(C3)N4)c12	8.49
BDBM50572109	CC(C)c1nccc2CCCCOc3cccc(F)c3-c3nc4n(-c12)c(=O)nc(N1C[C@@H](C)N(C[C@@H]1C)C(=O)C=C)c4cc3F	8.46
CHEMBL4870399	C=CC(=O)N1C[C@H](C)N(c2nc(=O)n3c4nc(c(F)cc24)-c2c(F)cccc2OCCCCc2ccnc(C(C)C)c2-3)C[C@H]1C	8.46
BDBM50539760	CN1CCC[C@H]1COc1nc2CN(CCc2c(n1)N1CCN([C@@H](CC#N)C1)C(=O)C=C)c1cccc2cccc(c12)C(F)(F)F	8.4
CHEMBL4636611	C=CC(=O)N1CCN(c2nc(OC[C@@H]3CCCN3C)nc3c2CCN(c2cccc4cccc(C(F)(F)F)c24)C3)C[C@@H]1CC#N	8.4
BDBM50572110	CC(C)c1nccc2CCCOc3cccc(F)c3-c3nc4n(-c12)c(=O)nc(N1CCN(C[C@@H]1C)C(=O)C=C)c4cc3F	8.37
CHEMBL4866126	C=CC(=O)N1CCN(c2nc(=O)n3c4nc(c(F)cc24)-c2c(F)cccc2OCCCc2ccnc(C(C)C)c2-3)[C@@H](C)C1	8.37
BDBM50579592	CC(C)c1nccc(C)c1-n1c2nc(c(Cl)cc2c(N2CCN(CC2)C(=O)C=C)c(C#N)c1=O)-c1ccccc1F	8.36
CHEMBL4876344	C=CC(=O)N1CCN(c2c(C#N)c(=O)n(-c3c(C)ccnc3C(C)C)c3nc(-c4ccccc4F)c(Cl)cc23)CC1	8.36
BDBM50567179	CCC1(CCNC1)NC(=O)c1cc(N2CCN([C@@H](CC#N)C2)C(=O)C=C)c2CCN(Cc2n1)c1cccc2cccc(C)c12	8.35
CHEMBL4860377	C=CC(=O)N1CCN(c2cc(C(=O)NC3(CC)CCNC3)nc3c2CCN(c2cccc4cccc(C)c24)C3)C[C@@H]1CC#N	8.35
BDBM50579591	CC(C)c1ncnc(C(C)C)c1-n1c2nc(c(Cl)cc2c(N2CCN(CC2)C(=O)C=C)c(C#N)c1=O)-c1c(N)c(Cl)cc(Cl)c1F	8.33
BDBM50567175	CN1CCCC1CNC(=O)c1cc(N2CCN([C@@H](CC#N)C2)C(=O)C=C)c2CCN(Cc2n1)c1cccc2cccc(C)c12	8.32
CHEMBL4857822	C=CC(=O)N1CCN(c2c(C#N)c(=O)n(-c3c(C(C)C)ncnc3C(C)C)c3nc(-c4c(N)c(Cl)cc(Cl)c4F)c(Cl)cc23)CC1	8.32
CHEMBL4866701	C=CC(=O)N1CCN(c2cc(C(=O)NCC3CCCN3C)nc3c2CCN(c2cccc4cccc(C)c24)C3)C[C@@H]1CC#N	8.32

CHEMBL4461434	C=CC(=O)N1C[C@@H]2C(=O)N(C)c3cnc4c(F)c(-c5c(O)cccc5F)c(Cl)cc4c3N2C[C@H]1C	8.3
CHEMBL4860108	C=C(F)C(=O)N1CCN(c2cc(C(=O)NC(C)CN3CCC(C)(O)C3)nc3c2CCN(c2cccc4cccc(C)c24)C3)C[C@@H]1CC#N	8.3
CHEMBL4879059	Cc1ccnc(C(C)C)c1-n1c(=O)nc(N2CCN(C(=O)[C@@H]3O[C@H]3CN3CCCCC3)C[C@@H]2C)c2cc(Cl)c(-c3ccccc3F)nc21	8.3
BDBM50567173	CC(CN1CCC(C)(O)C1)NC(=O)c1cc(N2CCN([C@@H](CC#N)C2)C(=O)C(F)=C)c2CCN(Cc2n1)c1cccc2cccc(C)c12	8.3
BDBM50527057	C[C@@H]1CN2[C@H](CN1C(=O)C=C)C(=O)N(C)c1cnc3c(F)c(c(Cl)cc3c21)-c1c(O)cccc1F	8.3
CHEMBL4594350	C=C(F)C(=O)N1CCN(c2nc(OC[C@@H]3CCCN3C)nc3c2CCN(c2cccc4cccc(Cl)c24)C3)C[C@@H]1CC#N	8.3
BDBM50539763	CN1CCC[C@H]1COc1nc2CN(CCc2c(n1)N1CCN([C@@H](CC#N)C1)C(=O)C(F)=C)c1cccc2cccc(Cl)c12	8.3
CHEMBL4867096	CCOC(=O)[C@@H]1O[C@H]1C(=O)N1CCN(c2nc(=O)n(-c3c(C)ccnc3C(C)C)c3nc(-c4ccccc4F)c(Cl)cc23)[C@@H](C)C1	8.3
BDBM50573426	CCOC(=O)[C@@H]1O[C@H]1C(=O)N1CCN([C@@H](C)C1)c1nc(=O)n(-c2c(C)ccnc2C(C)C)c2nc(c(Cl)cc12)-c1ccccc1F	8.3
BDBM50573427	CC(C)c1nccc(C)c1-n1c2nc(c(Cl)cc2c(nc1=O)N1CCN(C[C@@H]1C)C(=O)[C@@H]1O[C@H]1CN1CCCCC1)-c1ccccc1F	8.3
CHEMBL4867102	C=CC(=O)N1CCN(c2c(C#N)c(=O)n(-c3c(C)ccnc3C(C)C)c3nc(-c4c(N)c(Cl)cc(Cl)c4F)c(Cl)cc23)CC1CC#N	8.29
CHEMBL4852554	CN(C)CC1(COc2nc(N3CC4CCC(C3)N4)c3cc(F)c(-c4cc(O)cc5ccccc45)c(F)c3n2)CC1	8.29
BDBM50576731	CN(C)CC1(COc2nc(N3CC4CCC(C3)N4)c3cc(F)c(c(F)c3n2)-c2cc(O)cc3ccccc23)CC1	8.29
BDBM50579590	CC(C)c1nccc(C)c1-n1c2nc(c(Cl)cc2c(N2CCN(C(CC#N)C2)C(=O)C=C)c(C#N)c1=O)-c1c(N)c(Cl)cc(Cl)c1F	8.28
CHEMBL4847146	C=CC(=O)N1CCN(c2cc(C(=O)NC3(C)CCCNC3)nc3c2CCN(c2cccc4cccc(C)c24)C3)C[C@@H]1CC#N	8.27
BDBM50567180	Cc1cccc2cccc(N3CCc4c(C3)nc(cc4N3CCN([C@@H](CC#N)C3)C(=O)C=C)C(=O)NC3(C)CCCNC3)c12	8.27
BDBM50539759	CCc1cccc2cccc(N3CCc4c(C3)nc(OC[C@@H]3CCCN3C)nc4N3CCN([C@@H](CC#N)C3)C(=O)C=C)c12	8.22
CHEMBL4646899	C=CC(=O)N1CCN(c2nc(OC[C@@H]3CCCN3C)nc3c2CCN(c2cccc4cccc(CC)c24)C3)C[C@@H]1CC#N	8.22
BDBM544213	CC(C)c1ncnc(C2CC2)c1-n1c2nc(c(Cl)cc2c(nc1=O)N1C[C@@H](C)N(C[C@@H]1C)C(=O)C=C)-c1ccccc1F	8.22
BDBM544157	CC(C)c1ccccc1-c1nc2n(-c3c(ncnc3C(C)C)C(C)C)c(=O)nc(N3C[C@@H](C)N(C[C@@H]3C)C(=O)C=C)c2cc1Cl	8.22
CHEMBL4846583	C=CC(=O)N1CCN(c2cc(C(=O)NC(C)CN3CCC(C)(O)C3)nc3c2CCN(c2cccc4cccc(C)c24)C3)C[C@@H]1CC#N	8.21
CHEMBL4852249	C=CC(=O)N1CCN(c2cc(C(=O)NC[C@H]3CCCN3)nc3c2CCN(c2cccc4cccc(C)c24)C3)C[C@@H]1CC#N	8.21
BDBM50567177	Cc1cccc2cccc(N3CCc4c(C3)nc(cc4N3CCN([C@@H](CC#N)C3)C(=O)C=C)C(=O)NC[C@H]3CCCN3)c12	8.21
BDBM50567172	CC(CN1CCC(C)(O)C1)NC(=O)c1cc(N2CCN([C@@H](CC#N)C2)C(=O)C=C)c2CCN(Cc2n1)c1cccc2cccc(C)c12	8.21
CHEMBL4859387	C=CC(=O)N1CCN(c2c(C#N)c(=O)n(-c3c(C)ccnc3C(C)C)c3nc(-c4c(N)c(Cl)cc(Cl)c4F)c(Cl)cc23)C[C@@H]1CC#N	8.18
BDBM50579586	CC(C)c1nccc(C)c1-n1c2nc(c(Cl)cc2c(N2CCN([C@@H](CC#N)C2)C(=O)C=C)c(C#N)c1=O)-c1c(N)c(Cl)cc(Cl)c1F	8.17
BDBM535466	C[C@H]1CN(CCN1c1nc(OC[C@@H]2CCCN2C)nc2c(F)c(c(Cl)cc12)-c1nc(N)cc2ccccc12)C(=O)C=C	8.15
BDBM544106	CC(C)c1nccc(C)c1-n1c2nc(c(Cl)cc2c(nc1=O)N1C[C@@H](C)N(C[C@@H]1C)C(=O)C=C)-c1ccc(F)cc1F	8.1
BDBM544283	CC(C)c1nccc(C)c1-n1c2nc(c(Cl)cc2c(nc1=O)N1C[C@@H](C)N(C[C@@H]1C)C(=O)C=C)-c1c(F)cccc1NC(=O)C1CC1	8.1
BDBM535155	C[C@H]1CN(CCN1c1nc(OC[C@@H]2CCCN2C)nc2c(F)c(c(Cl)cc12)-c1nc(N)cc(C)c1C(F)(F)F)C(=O)\C=C\CF	8.1
CHEMBL4456801	C=CC(=O)N1CCN(c2nc(=O)n(-c3ccccc3C(C)C)c3cc(-c4c(C)ccc5[nH]ncc45)c(Cl)cc23)CC1	8.1
BDBM50514386	CC(C)c1ccccc1-n1c2cc(c(Cl)cc2c(nc1=O)N1CCN(CC1)C(=O)C=C)-c1c(C)ccc2[nH]ncc12	8.1
BDBM50567174	C[C@H](CN(C)C)NC(=O)c1cc(N2CCN([C@@H](CC#N)C2)C(=O)C(F)=C)c2CCN(Cc2n1)c1cccc2cccc(C)c12	8.08
CHEMBL4847380	C=CC(=O)N1CCN(c2cc(C(=O)NCC3CCCCN3)nc3c2CCN(c2cccc4cccc(C)c24)C3)C[C@@H]1CC#N	8.08
CHEMBL4847011	C=C(F)C(=O)N1CCN(c2cc(C(=O)N[C@H](C)CN(C)C)nc3c2CCN(c2cccc4cccc(C)c24)C3)C[C@@H]1CC#N	8.08
BDBM50567178	Cc1cccc2cccc(N3CCc4c(C3)nc(cc4N3CCN([C@@H](CC#N)C3)C(=O)C=C)C(=O)NCC3CCCCN3)c12	8.08
BDBM50539766	COC\C=C\C(=O)N1CCN(C[C@@H]1CC#N)c1nc(OC[C@@H]2CCCN2C)nc2CN(CCc12)c1cccc2cccc(Cl)c12	8.05
CHEMBL4645376	COC/C=C/C(=O)N1CCN(c2nc(OC[C@@H]3CCCN3C)nc3c2CCN(c2cccc4cccc(Cl)c24)C3)C[C@@H]1CC#N	8.05
CHEMBL4878409	CN(C)CC1(COc2nc3c(c(N4CC5CCC(C4)N5)n2)CCN(c2cc(O)cc4cccc(Br)c24)C3)CC1(F)F	8.01
BDBM50576732	CN(C)CC1(COc2nc3CN(CCc3c(n2)N2CC3CCC(C2)N3)c2cc(O)cc3cccc(Br)c23)CC1(F)F	8.01
BDBM535253	CN1CCC[C@H]1COc1nc(N2CCN(C(CF)C2)C(=O)C(F)=C)c2cc(Cl)c(cc2n1)-c1nc(N)cc(C)c1C(F)(F)F	8

BDBM535490	COCC1CCC(COc2nc(N3CCN(CC3)C(=O)C=C)c3cc(Cl)c(cc3n2)-c2nc(N)ccc2C(F)(F)F)N1C	8
BDBM535487	CN1C[C@H]2C[C@H]2[C@H]1COc1nc(N2CCN(CC2)C(=O)C=C)c2cc(Cl)c(cc2n1)-c1nc(N)ccc1C(F)(F)F	8
BDBM535468	C[C@H]1CN(CCN1c1nc(OCC2CCC(=O)N2C)nc2c(F)c(c(Cl)cc12)-c1nc(N)cc(C)c1C(F)(F)F)C(=O)C=C	8
BDBM535464	CNc1cc(C)c(c(n1)-c1c(Cl)cc2c(nc(OC[C@@H]3CCCN3C)nc2c1F)N1CCN(C[C@@H]1C)C(=O)C=C)C(F)(F)F	8
BDBM535486	CN1C[C@@H]2C[C@@H]2[C@H]1COc1nc(N2CCN(CC2)C(=O)C=C)c2cc(Cl)c(cc2n1)-c1nc(N)ccc1C(F)(F)F	8
BDBM535463	C[C@H]1CN(CCN1c1nc(OC[C@@H]2CCCN2C)nc2cc(c(Cl)cc12)-c1nc(N)cc(C)c1C(F)(F)F)C(=O)C=C	8
BDBM535296	C[C@H]1CN(CCN1c1nc(OC[C@@H]2CCCN2C)nc2c(F)c(c(SC(F)(F)F)cc12)-c1nc(N)cc(C)c1C(F)(F)F)C(=O)C=C	8
BDBM535485	CN1CC[C@H](F)[C@H]1COc1nc(N2CCN(CC2)C(=O)C=C)c2cc(Cl)c(cc2n1)-c1nc(N)ccc1C(F)(F)F	8
CHEMBL4648671	C=CC(=O)N1CCN(c2nc(OC[C@@H]3CCCN3C)nc3c2CCN(c2cccc4ccccc24)C3)C[C@@H]1CC#N	8
BDBM535472	C[C@H]1CN(CCN1c1nc(OC[C@@H]2CCCN2C)nc2c(F)c(c(Cl)cc12)-c1nc(N)c(F)c2ccccc12)C(=O)C=C	8
BDBM535513	C[C@H]1CN(CCN1c1nc(Oc2cccc3CN(C)CCc23)nc2c(F)c(c(Cl)cc12)-c1nc(N)cc(C)c1C(F)(F)F)C(=O)C=C	8
BDBM535499	C[C@H]1CN(CCN1c1nc(OC[C@@H]2C[C@@H](F)CN2C)nc2c(F)c(c(Cl)cc12)-c1nc(N)cc(C)c1C)C(=O)C=C	8
BDBM535482	CN1CC[C@@H]1COc1nc(N2CCN(CC2)C(=O)C=C)c2cc(Cl)c(cc2n1)-c1nc(N)ccc1C(F)(F)F	8
BDBM535152	C[C@H]1CN(CCN1c1nc(OC[C@@H]2CCCN2C)nc2c(F)c(c(Cl)cc12)-c1nc(N)cc(C)c1C(F)(F)F)C(=O)C=C	8
BDBM535223	CN1CCC[C@H]1COc1nc(N2CCN(C(CC#N)C2)C(=O)C(F)=C)c2cc(Cl)c(cc2n1)-c1nc(N)cc(C)c1C(F)(F)F	8
BDBM535477	CN1CCC[C@H]1COc1nc(N2CCN(CC2)C(=O)C=C)c2cc(Cl)c(cc2n1)-c1nc(N)ccc1C(F)(F)F	8
BDBM535476	C[C@H]1CN(CCN1c1nc(OC[C@@H]2C[C@@H](F)CN2C)nc2c(F)c(c(Cl)cc12)-c1nc(N)cc(C)c1C(F)(F)F)C(=O)C=C	8
BDBM535474	C[C@H]1CN(CCN1c1nc(OC[C@@H]2CCCN2C)nc2c(F)c(c(Cl)cc12)-c1nc(N)cc(C)c1C(F)(F)F)C(=O)C(F)=C	8
BDBM535512	CN1C[C@H](F)CC1(C)COc1nc(N2CCN(CC2)C(=O)C=C)c2cc(Cl)c(cc2n1)-c1nc(N)ccc1C(F)(F)F	8
BDBM535495	CN1C[C@H](F)C[C@H]1COc1nc(N2CCN([C@H](CC#N)C2)C(=O)C(F)=C)c2cc(Cl)c(c(F)c2n1)-c1nc(N)cc(C)c1C(F)(F)F	8
BDBM544382	CC(C)c1nc(Br)nc(C)c1-n1c2nc(c(Cl)cc2c(nc1=O)N1C[C@@H](C)N(C[C@@H]1C)C(=O)C=C)-c1ccccc1F	8
BDBM535491	C[C@H]1CN(CCN1c1nc(OC[C@@H]2C[C@@H](F)CN2C)nc2c(F)c(c(F)cc12)-c1nc(N)cc(C)c1C(F)(F)F)C(=O)C=C	8
BDBM50539754	CN1CCC[C@H]1COc1nc2CN(CCc2c(n1)N1CCN([C@@H](CC#N)C1)C(=O)C=C)c1cccc2ccccc12	8
BDBM544107	COc1c(F)cccc1-c1nc2n(-c3c(C)ccnc3C(C)C)c(=O)nc(N3C[C@@H](C)N(C[C@@H]3C)C(=O)C=C)c2cc1Cl	8
BDBM515895	COc1nccc2CN(CCc12)C(=O)c1c(C2CC2)n(CC(=O)NC2CN(C2)C(=O)C=C)c2c(C)cc(Cl)cc12	8
BDBM548364	CNC(=O)C[C@H](CC(C)C)Nc1nc(nc2cc(ccc12)-c1nccs1)N1CCC2(CN(C2)C(=O)C=C)C1	8
BDBM535493	C[C@H]1CN(CCN1c1nc(OC[C@@H]2C[C@@H](F)CN2C)nc2c(F)c(c(Cl)cc12)-c1nc(N)cc(C)c1C(F)(F)F)C(=O)C(F)=C	8
BDBM50514393	CC(C)c1nccc(C)c1-n1c2nc(c(Cl)cc2c(nc1=O)N1CCN(C[C@@H]1C)C(=O)C=C)-c1c(O)cccc1F	7.96
CHEMBL4539214	C=CC(=O)N1CCN(c2nc(=O)n(-c3c(C)ccnc3C(C)C)c3nc(-c4c(O)cccc4F)c(Cl)cc23)[C@@H](C)C1	7.96
BDBM535505	COCCN1C[C@H](F)C[C@H]1COc1nc(N2CCN(CC2)C(=O)C=C)c2cc(Cl)c(cc2n1)-c1nc(N)ccc1C(F)(F)F	7.96
BDBM535478	CCO[C@@H]1C[C@@H](COc2nc(N3CCN(CC3)C(=O)C=C)c3cc(Cl)c(cc3n2)-c2nc(N)ccc2C(F)(F)F)N(C)C1	7.96
BDBM535233	CN1C[C@H](F)C[C@H]1COc1nc(N2CCN(C(CC#N)C2)C(=O)C(F)=C)c2cc(Cl)c(cc2n1)-c1nc(N)cc(C)c1C(F)(F)F	7.96
BDBM535471	C[C@H]1CN(CCN1c1nc(OC2CCCC2N(C)C)nc2cc(c(Cl)cc12)-c1nc(N)cc(C)c1C(F)(F)F)C(=O)C=C	7.96
BDBM50521250	Cc1ccc2[nH]ncc2c1-c1cc2ncnc3N4CCN(C[C@H]4COc(c1Cl)c23)C(=O)C=C	7.92
BDBM535501	C[C@H]1CN(CCN1c1nc(OC[C@@H]2CCCN2C)nc2c(F)c(c(Cl)cc12)-c1nc(N)cc(C)c1C(F)(F)F)C(=O)\C=C\CO	7.92
BDBM535508	CN1C[C@@H](C[C@H]1COc1nc(N2CCN(CC2)C(=O)C=C)c2cc(Cl)c(cc2n1)-c1nc(N)ccc1C(F)(F)F)OC(F)F	7.92
BDBM535506	C[C@H]1[C@H](F)C[C@@H](COc2nc(N3CCN(CC3)C(=O)C=C)c3cc(Cl)c(cc3n2)-c2nc(N)ccc2C(F)(F)F)N1C	7.92
CHEMBL4568148	C=CC(=O)N1CCN2c3nc(NC4CCN(C5CC5)CC4)nc4cc(-c5c(C)ccc6[nH]ncc56)c(Cl)c(c34)OC[C@@H]2C1	7.92
CHEMBL4475526	C=CC(=O)N1CCN2c3ncnc4cc(-c5c(C)ccc6[nH]ncc56)c(Cl)c(c34)OC[C@@H]2C1	7.92
BDBM50521252	Cc1ccc2[nH]ncc2c1-c1cc2nc(NC3CCN(CC3)C3CC3)nc3N4CCN(C[C@H]4COc(c1Cl)c23)C(=O)C=C	7.92
BDBM535507	COCCN1CCC[C@H]1COc1nc(N2CCN(CC2)C(=O)C=C)c2cc(Cl)c(cc2n1)-c1nc(N)ccc1C(F)(F)F	7.92

BDBM535481	CN1CCC[C@H]1C(C)(C)Oc1nc(N2CCN(CC2)C(=O)C=C)c2cc(Cl)c(cc2n1)-c1nc(N)ccc1C(F)(F)F	7.92
BDBM535511	COCCN1C[C@@H](C[C@H]1COc1nc(N2CCN(CC2)C(=O)C=C)c2cc(Cl)c(cc2n1)-c1nc(N)ccc1C(F)(F)F)OC	7.89
BDBM535484	Nc1ccc(c(n1)-c1cc2nc(OCCN3CC(CF)C3)nc(N3CCN(CC3)C(=O)C=C)c2cc1Cl)C(F)(F)F	7.89
BDBM535479	CN1[C@H](COc2nc(N3CCN(CC3)C(=O)C=C)c3cc(Cl)c(cc3n2)-c2nc(N)ccc2C(F)(F)F)CCC11CC1	7.89
BDBM535470	C[C@H]1CN(CCN1c1nc(OC[C@@H]2CCCN2C)nc2cc(c(Cl)cc12)-c1nc(N)cc(C)c1C(F)(F)F)C(=O)\C=C\C(F)F	7.89
BDBM535157	C[C@H]1CN(CCN1c1ncnc2c(F)c(c(Cl)cc12)-c1nc(N)cc(C)c1C(F)(F)F)C(=O)C=C	7.89
BDBM50539753	CN1CCC[C@H]1COc1nc2CN(CCc2c(n1)N1CCN(C(CC#N)C1)C(=O)C=C)c1cccc2ccccc12	7.85
CHEMBL4648056	C=CC(=O)N1CCN(c2nc(OC[C@@H]3CCCN3C)nc3c2CCN(c2cccc4ccccc24)C3)CC1CC#N	7.85
BDBM535260	CC1CN([C@H](CC#N)CN1c1nc(OC[C@@H]2C[C@@H](F)CN2C)nc2cc(c(Cl)cc12)-c1nc(N)cc(C)c1C(F)(F)F)C(=O)C(F)=C	7.85
BDBM544076	CC(C)c1nccc(C)c1-n1c2nc(c(Cl)cc2c(nc1=O)N1C[C@@H](C)N(C[C@@H]1C)C(=O)C=C)-c1cc(F)c(F)cc1N	7.85
BDBM535137	Nc1ccc(c(n1)-c1c(Cl)cc2c(ncnc2c1F)N1CCN(CC1)C(=O)C=C)C(F)(F)F	7.85
BDBM535462	C[C@H]1CN(C[C@H](C)N1c1ncnc2cc(c(Cl)cc12)-c1nc(N)cc(C)c1C(F)(F)F)C(=O)C=C	7.85
BDBM544428	CC(C)c1nccc(C)c1-n1c2nc(c(Cl)cc2c(nc1=O)N1CCN([C@@H]2C[C@H]12)C(=O)C=C)-c1ccccc1F	7.85
BDBM50573432	CC(C)c1nccc(C)c1-n1c2nc(c(Cl)cc2c(nc1=O)N1C[C@@H](C)N(C[C@@H]1C)C(=O)[C@H]1CO1)-c1c(N)cccc1F	7.82
BDBM535201	Cc1cc(N)nc(-c2cc3ncnc(N4CCN(CC4)C(=O)C=C)c3cc2Cl)c1C(F)(F)F	7.82
CHEMBL4854390	Cc1ccnc(C(C)C)c1-n1c(=O)nc(N2C[C@@H](C)N(C(=O)[C@H]3CO3)C[C@@H]2C)c2cc(Cl)c(-c3c(N)cccc3F)nc21	7.82
CHEMBL4591572	C=CC(=O)N1CCN2c3c(cnc4c(F)c(-c5c(O)cccc5F)c(Cl)cc34)OC[C@H]2C1	7.8
BDBM50527051	Oc1cccc(F)c1-c1c(Cl)cc2c3N4CCN(C[C@@H]4COc3cnc2c1F)C(=O)C=C	7.8
BDBM544046	CC(C)c1ccccc1-c1nc2n(-c3c(C)ccnc3C(C)C)c(=O)nc(N3C[C@@H](C)N(C[C@@H]3C)C(=O)C=C)c2cc1Cl	7.8
CHEMBL3577123	CNc1ncc2cc(-c3cc(NC(=O)NCCC(C)(C)C)c(F)cc3C)cnc2n1	7.8
BDBM535488	C[C@H](CN(C)C)Oc1nc(N2CCN(CC2)C(=O)C=C)c2cc(Cl)c(cc2n1)-c1nc(N)ccc1C(F)(F)F	7.77
BDBM535128	Fc1c(c(Cl)cc2c(ncnc12)N1CCN(CC1)C(=O)C=C)-c1nccc2ccccc12	7.77
CHEMBL4566835	C=CC(=O)N1CCN2c3nc(NCCN(C)C)nc4cc(-c5c(C)ccc6[nH]ncc56)c(Cl)c(c34)OC[C@@H]2C1	7.74
BDBM50521253	CN(C)CCNc1nc2N3CCN(C[C@H]3COc3c(Cl)c(cc(n1)c23)-c1c(C)ccc2[nH]ncc12)C(=O)C=C	7.74
CHEMBL4452974	C=CC(=O)N1CCN2c3c(cnc4c(F)c(-c5c(O)cccc5F)c(Cl)cc34)N(C)C(=O)[C@H]2C1	7.74
BDBM50573429	CC(C)c1nccc(C)c1-n1c2nc(c(Cl)cc2c(nc1=O)N1CC(C1)NC(=O)C1CO1)-c1ccccc1F	7.74
BDBM50527059	CN1C(=O)[C@H]2CN(CCN2c2c1cnc1c(F)c(c(Cl)cc21)-c1c(O)cccc1F)C(=O)C=C	7.74
CHEMBL4852058	Cc1ccnc(C(C)C)c1-n1c(=O)nc(N2CC(NC(=O)C3CO3)C2)c2cc(Cl)c(-c3ccccc3F)nc21	7.74
CHEMBL4853619	CN1C[C@H](F)C[C@H]1COc1nc2c(c(N3CC4CCC(C3)N4)n1)CCN(c1cc(O)cc3cccc(Br)c13)C2	7.73
CHEMBL4443236	C=CC(=O)N1CCN2c3nc(N4CC(N(C)C)C4)nc4cc(-c5c(C)ccc6[nH]ncc56)c(Cl)c(c34)OC[C@@H]2C1	7.72
BDBM535183	Cc1nc(N2CCN(CC2)C(=O)C=C)c2cc(Cl)c(cc2n1)-c1nc(N)cc(C)c1C(F)(F)F	7.72
BDBM548305	CNC(=O)C[C@H](CC(C)C)Nc1nc(nc2CC(C)CCc12)N1CC2(CN(C2)C(=O)C=C)C(F)(F)C1	7.72
BDBM544069	CC(C)c1nccc(C)c1-n1c2nc(c(Cl)cc2c(nc1=O)N1C[C@@H](C)N(C[C@@H]1C)C(=O)C=C)-c1c(F)cccc1C(N)=O	7.72
BDBM544057	CC(C)c1nccc(C)c1-n1c2nc(c(Cl)cc2c(nc1=O)N1C[C@@H](C)N(C[C@@H]1C)C(=O)C=C)-c1ccccc1N	7.72
BDBM50521251	CN(C)C1CN(C1)c1nc2N3CCN(C[C@H]3COc3c(Cl)c(cc(n1)c23)-c1c(C)ccc2[nH]ncc12)C(=O)C=C	7.72
BDBM50576733	CN1C[C@H](F)C[C@H]1COc1nc2CN(CCc2c(n1)N1CC2CCC(C1)N2)c1cc(O)cc2cccc(Br)c12	7.72
BDBM544105	CC(C)c1nccc(C)c1-n1c2nc(c(Cl)cc2c(nc1=O)N1C[C@@H](C)N(C[C@@H]1C)C(=O)C=C)-c1cc(C)ccc1F	7.7
BDBM544417	CC(C)c1nccc(C)c1-n1c2nc(c(Cl)cc2c(nc1=O)N1C[C@@H](C)N(C[C@@H]1C)C(=O)C=C)-c1c(F)cccc1S(C)(=O)=O	7.7
BDBM535496	CN1C[C@H](F)C[C@H]1COc1nc(N2CCN([C@@H](CC#N)C2)C(=O)C(F)=C)c2cc(Cl)c(c(F)c2n1)-c1nc(N)cc(C)c1C(F)(F)F	7.7
CHEMBL4872788	Fc1c(-c2cccc3c2CC2CC32)ncc2c(N3CC4CCC(C3)N4)nc(OC[C@@]34CCCN3C[C@H](F)C4)nc12	7.7
BDBM50579605	F[C@H]1CN2CCC[C@@]2(COc2nc(N3CC4CCC(C3)N4)c3cnc(c(F)c3n2)-c2cccc3C4CC4Cc23)C1	7.7

BDBM544100	CCc1ccccc1-c1nc2n(-c3c(C)ccnc3C(C)C)c(=O)nc(N3C[C@@H](C)N(C[C@@H]3C)C(=O)C=C)c2cc1Cl	7.68
CHEMBL4434842	C=CC(=O)N1CCN(c2nc(=O)n(-c3c(C(C)C)ncnc3C(C)C)c3nc(-c4c(O)cccc4F)c(Cl)cc23)[C@@H](C)C1	7.68
BDBM50514400	CC(C)c1ncnc(C(C)C)c1-n1c2nc(c(Cl)cc2c(nc1=O)N1CCN(C[C@@H]1C)C(=O)C=C)-c1c(O)cccc1F	7.68
CHEMBL4648124	C=CC(=O)N1CCN(c2nc(OC[C@@H]3CCCN3C)nc3c2CCN(c2cccc4cccc(OC)c24)C3)C[C@@H]1CC#N	7.66
BDBM548347	CNC(=O)C[C@H](CC(C)C)Nc1nc(nc2cc(OC)cnc12)N1CCC2(CN(C2)C(=O)C=C)C1	7.66
BDBM544248	CC(C)c1ccnc(C(C)C)c1-n1c2nc(c(Cl)cc2c(nc1=O)N1C[C@@H](C)N(C[C@@H]1C)C(=O)C=C)-c1c(O)cccc1F	7.66
BDBM50539757	COc1cccc2cccc(N3CCc4c(C3)nc(OC[C@@H]3CCCN3C)nc4N3CCN([C@@H](CC#N)C3)C(=O)C=C)c12	7.66
BDBM535510	Nc1ccc(c(n1)-c1cc2nc(OC[C@@H]3CCCN3CC(F)F)nc(N3CCN(CC3)C(=O)C=C)c2cc1Cl)C(F)(F)F	7.64
BDBM535503	Nc1ccc(c(n1)-c1cc2nc(OC[C@@H]3CCCN3C3COC3)nc(N3CCN(CC3)C(=O)C=C)c2cc1Cl)C(F)(F)F	7.64
BDBM548341	CNC(=O)C[C@H](CC(C)C)Nc1nc(nc2cc(cnc12)-c1nccs1)N1CCC2(CN(C2)C(=O)C=C)C1	7.64
BDBM543968	CC(C)c1nccc(N(C)C)c1-n1c2nc(c(Cl)cc2c(nc1=O)N1C[C@@H](C)N(C[C@@H]1C)C(=O)C=C)-c1c(N)cccc1F	7.64
BDBM544091	CC(C)c1nccc(C)c1-n1c2nc(c(Cl)cc2c(nc1=O)N1C[C@@H](C)N(C[C@@H]1C)C(=O)C=C)-c1cccc(Cl)c1F	7.64
CHEMBL4858243	Cc1ccnc(C(C)C)c1-n1c(=O)nc(N2C[C@@H](C)N(C(=O)[C@H]3CO3)C[C@@H]2C)c2cc(Cl)c(-c3c(O)cccc3F)nc21	7.62
BDBM50573431	CC(C)c1nccc(C)c1-n1c2nc(c(Cl)cc2c(nc1=O)N1C[C@@H](C)N(C[C@@H]1C)C(=O)[C@H]1CO1)-c1c(O)cccc1F	7.62
BDBM548358	CC(C)C[C@@H](Cc1cc(C)on1)Nc1nc(nc2CC(C)(C)CCc12)N1CCC2(CN(C2)C(=O)C=C)C1	7.62
BDBM544047	CC(C)c1nccc(C)c1-n1c2nc(c(Cl)cc2c(nc1=O)N1C[C@@H](C)N(C[C@@H]1C)C(=O)C=C)-c1cccc2cc[nH]c12	7.62
CHEMBL4464232	C=CC(=O)N1CCN(c2nc(=O)n(-c3c(C)cccc3CC)c3nc(-c4c(O)cccc4F)c(Cl)cc23)[C@@H](C)C1	7.6
CHEMBL4452137	C=CC(=O)N1CCN(c2nc(=O)n(-c3c(C)cccc3C(C)C)c3nc(-c4c(O)cccc4F)c(Cl)cc23)[C@@H](C)C1	7.6
BDBM544090	CC(C)c1nccc(C)c1-n1c2nc(c(Cl)cc2c(nc1=O)N1C[C@@H](C)N(C[C@@H]1C)C(=O)C=C)-c1ccccc1C	7.6
BDBM544306	CC(C)c1cccc2C\C=C\Cc3ccccc3-c3nc4n(-c12)c(=O)nc(N1C[C@@H](C)N(C[C@@H]1C)C(=O)C=C)c4cc3Cl	7.6
BDBM535504	CN(C)C1(COc2nc(N3CCN(CC3)C(=O)C=C)c3cc(Cl)c(cc3n2)-c2nc(N)ccc2C(F)(F)F)CC1	7.6
BDBM535214	CCc1cc(N)nc(-c2cc3ncnc(N4CCN(CC4)C(=O)C=C)c3cc2Cl)c1C(F)(F)F	7.6
BDBM50514379	CC(C)c1cccc(C)c1-n1c2nc(c(Cl)cc2c(nc1=O)N1CCN(C[C@@H]1C)C(=O)C=C)-c1c(O)cccc1F	7.6
BDBM50514372	CCc1cccc(C)c1-n1c2nc(c(Cl)cc2c(nc1=O)N1CCN(C[C@@H]1C)C(=O)C=C)-c1c(O)cccc1F	7.6
BDBM544207	CNc1nc(C(C)C)c(c(n1)C(C)C)-n1c2nc(c(Cl)cc2c(nc1=O)N1C[C@@H](C)N(C[C@@H]1C)C(=O)C=C)-c1ccccc1F	7.59
CHEMBL4870135	COC(=O)/C=C/C(=O)N1CCN(c2nc(=O)n(-c3c(C)ccnc3C(C)C)c3nc(-c4ccccc4F)c(Cl)cc23)[C@@H](C)C1	7.59
BDBM535480	CN1CC(F)(F)CC1(C)COc1nc(N2CCN(CC2)C(=O)C=C)c2cc(Cl)c(cc2n1)-c1nc(N)ccc1C(F)(F)F	7.59
BDBM50573423	COC(=O)\C=C\C(=O)N1CCN([C@@H](C)C1)c1nc(=O)n(-c2c(C)ccnc2C(C)C)c2nc(c(Cl)cc12)-c1ccccc1F	7.59
BDBM50573424	CC(C)c1nccc(C)c1-n1c2nc(c(Cl)cc2c(nc1=O)N1CCN(C[C@@H]1C)C(=O)[C@H]1CO1)-c1ccccc1F	7.57
CHEMBL4849074	Cc1ccnc(C(C)C)c1-n1c(=O)nc(N2CCN(C(=O)[C@H]3CO3)C[C@@H]2C)c2cc(Cl)c(-c3ccccc3F)nc21	7.57
BDBM544245	CC(C)c1ccnc(C(C)C)c1-n1c2nc(c(Cl)cc2c(nc1=O)N1C[C@@H](C)N(C[C@@H]1C)C(=O)C=C)-c1ccccc1F	7.57
CHEMBL4449810	C=CC(=O)N1CCN(c2nc(=O)n(-c3ccccc3C(C)C)c3cc(-c4c(O)cccc4F)c(Cl)cc23)[C@@H](C)C1	7.55
BDBM535502	C[C@H]1CN(CCN1c1nc(OC[C@@H]2CCCN2C)nc2c(F)c(c(Cl)cc12)-c1nc(N)cc(C)c1C(F)(F)F)C(=O)\C=C\CCl	7.55
BDBM50514388	CC(C)c1ccccc1-n1c2cc(c(Cl)cc2c(nc1=O)N1CCN(C[C@@H]1C)C(=O)C=C)-c1c(O)cccc1F	7.55
BDBM544247	CC(C)c1ccnc(C(C)C)c1-n1c2nc(c(Cl)cc2c(nc1=O)N1C[C@@H](C)N(C[C@@H]1C)C(=O)C=C)-c1ccccc1C(F)(F)F	7.55
BDBM544104	CC(C)c1nccc(C)c1-n1c2nc(c(Cl)cc2c(nc1=O)N1C[C@@H](C)N(C[C@@H]1C)C(=O)C=C)-c1cccc(F)c1C	7.55
BDBM544375	COc1ccc(CN(Cc2ccc(OC)cc2)c2ncnc(C(C)C)c2-n2c3nc(Cl)c(Cl)cc3c(nc2=O)N2C[C@@H](C)N(C[C@@H]2C)C(=O)C=C)cc1	7.54
CHEMBL4452819	C=CC(=O)N1CCN2c3ncnc4c(F)c(-c5c(O)cccc5F)c(Cl)c(c34)OC[C@@H]2C1	7.54
BDBM50521249	Oc1cccc(F)c1-c1c(Cl)c2OC[C@@H]3CN(CCN3c3ncnc(c1F)c23)C(=O)C=C	7.54
BDBM544002	CCc1ccnc(C(C)C)c1-n1c2nc(c(Cl)cc2c(nc1=O)N1C[C@@H](C)N(C[C@@H]1C)C(=O)C=C)-c1ccccc1F	7.52
BDBM50514402	CC(C)c1nccc(C)c1-n1c2nc(c(F)cc2c(nc1=O)N1CCN(C[C@@H]1C)C(=O)C=C)-c1c(O)cccc1F	7.52

BDBM544386	CC(C)c1nc(F)nc(C)c1-n1c2nc(c(Cl)cc2c(nc1=O)N1C[C@@H](C)N(C[C@@H]1C)C(=O)C=C)-c1ccccc1F	7.52
CHEMBL4535757	C=CC(=O)N1CCN(c2nc(=O)n(-c3c(C)ccnc3C(C)C)c3nc(-c4c(O)cccc4F)c(F)cc23)[C@@H](C)C1	7.52
BDBM544351	CC(C)c1nc(C=C)cc(C)c1-n1c2nc(c(Cl)cc2c(nc1=O)N1C[C@@H](C)N(C[C@@H]1C)C(=O)C=C)-c1ccccc1F	7.51
BDBM544052	CC(C)c1nccc(C)c1-n1c2nc(-c3cc(C)sc3C)c(Cl)cc2c(nc1=O)N1C[C@@H](C)N(C[C@@H]1C)C(=O)C=C	7.51
BDBM544171	CC(C)c1ncnc(Cl)c1-n1c2nc(c(Cl)cc2c(nc1=O)N1C[C@@H](C)N(C[C@@H]1C)C(=O)C=C)-c1ccccc1F	7.51
BDBM535210	COc1cc2c(ncnc2cc1-c1nc(N)cc2cccc(F)c12)N1CCN(CC1)C(=O)C=C	7.49
BDBM50521254	Oc1cccc(Cl)c1-c1cc2ncnc3N4CCN(C[C@H]4COc(c1Cl)c23)C(=O)C=C	7.49
BDBM535175	Cc1cc(N)nc(-c2cc3ncnc(N4CCN(CC4)C(=O)C(F)=C)c3cc2Cl)c1C(F)(F)F	7.49
BDBM544084	CC#Cc1ccc(F)c(c1)-c1nc2n(-c3c(C)ccnc3C(C)C)c(=O)nc(N3C[C@@H](C)N(C[C@@H]3C)C(=O)C=C)c2cc1Cl	7.49
CHEMBL4450793	C=CC(=O)N1CCN2c3ncnc4cc(-c5c(O)cccc5Cl)c(Cl)c(c34)OC[C@@H]2C1	7.49
BDBM544272	CC(C)c1ccnc(C(C)C)c1-n1c2nc(c(Cl)cc2c(nc1=O)N1CCN(C[C@@H]1C)C(=O)C=C)-c1ccccc1C	7.49
BDBM535208	Nc1ccc(c(n1)-c1cc2ncnc(N3CCN([C@H](C3)C#N)C(=O)C(F)=C)c2cc1Cl)C(F)(F)F	7.49
BDBM535177	Nc1cc2cccc(F)c2c(n1)-c1c(Cl)cc2c(ncnc2c1F)N1CCN(CC1)C(=O)C=C	7.49
BDBM544096	CC(C)c1nccc(C)c1-n1c2nc(c(Cl)cc2c(nc1=O)N1C[C@@H](C)N(C[C@@H]1C)C(=O)C=C)-c1ccccc1Cl	7.48
BDBM544246	CC(C)c1ccnc(C(C)C)c1-n1c2nc(c(Cl)cc2c(nc1=O)N1C[C@@H](C)N(C[C@@H]1C)C(=O)C=C)-c1ccccc1C	7.48
BDBM535182	CNc1nc(N2CCN(CC2)C(=O)C=C)c2cc(Cl)c(cc2n1)-c1nc(N)cc(C)c1C(F)(F)F	7.48
BDBM50514370	C[C@H]1CN(CCN1c1nc(=O)n(-c2c(ncnc2C2CC2)C2CC2)c2nc(c(Cl)cc12)-c1c(O)cccc1F)C(=O)C=C	7.47
CHEMBL4588277	C=CC(=O)N1CCN(c2nc(=O)n(-c3c(C4CC4)ncnc3C3CC3)c3nc(-c4c(O)cccc4F)c(Cl)cc23)[C@@H](C)C1	7.47
BDBM544111	CC(C)c1nccc(C)c1-n1c2nc(c(Cl)cc2c(nc1=O)N1C[C@@H](C)N(C[C@@H]1C)C(=O)C=C)-c1cccc(F)c1F	7.47
BDBM544252	CC(C)c1ccccc1-c1nc2n(-c3c(ccnc3C(C)C)C(C)C)c(=O)nc(N3C[C@@H](C)N(C[C@@H]3C)C(=O)C=C)c2cc1Cl	7.47
BDBM544271	CC(C)c1nccc(C2CC2)c1-n1c2nc(c(Cl)cc2c(nc1=O)N1C[C@@H](C)N(C[C@@H]1C)C(=O)C=C)-c1ccccc1C	7.46
BDBM544216	CC(C)c1ncnc(C2CC2)c1-n1c2nc(c(Cl)cc2c(nc1=O)N1C[C@@H](C)N(C[C@@H]1C)C(=O)C=C)-c1ccccc1C	7.46
BDBM535221	C[C@H]1CN(CCN1c1ncnc2cc(c(Cl)cc12)-c1nc(N)ccc1C(F)(F)F)C(=O)C=C	7.46
BDBM50514383	CCc1cccc(CC)c1-n1c2nc(c(Cl)cc2c(nc1=O)N1CCN(C[C@@H]1C)C(=O)C=C)-c1c(O)cccc1F	7.44
BDBM544369	CC(C)c1ncnc(N)c1-n1c2nc(c(Cl)cc2c(nc1=O)N1C[C@@H](C)N(C[C@@H]1C)C(=O)C=C)-c1ccccc1F	7.44
CHEMBL4554946	C=CC(=O)N1CCN(c2nc(=O)n(-c3c(CC)cccc3CC)c3nc(-c4c(O)cccc4F)c(Cl)cc23)[C@@H](C)C1	7.44
BDBM544250	CC(C)c1ccnc(C(C)C)c1-n1c2nc(c(Cl)cc2c(nc1=O)N1C[C@@H](C)N(C[C@@H]1C)C(=O)C=C)-c1ccccc1Cl	7.43
BDBM544368	COc1ccc(CNc2nc(C(C)C)c(c(n2)C(C)C)-n2c3nc(c(Cl)cc3c(nc2=O)N2C[C@@H](C)N(C[C@@H]2C)C(=O)C=C)-c2ccccc2F)cc1	7.42
CHEMBL4527861	C=CC(=O)N1CCN(c2nc(=O)n(-c3c(C)ncnc3C(C)C)c3nc(-c4c(O)cccc4F)c(Cl)cc23)[C@@H](C)C1	7.42
BDBM544068	CC(C)c1nccc(C)c1-n1c2nc(c(Cl)cc2c(nc1=O)N1C[C@@H](C)N(C[C@@H]1C)C(=O)C=C)-c1ccccc1C(F)(F)F	7.42
BDBM50514395	CC(C)c1ncnc(C)c1-n1c2nc(c(Cl)cc2c(nc1=O)N1CCN(C[C@@H]1C)C(=O)C=C)-c1c(O)cccc1F	7.42
BDBM50573422	CC(C)c1nccc(C)c1-n1c2nc(c(Cl)cc2c(nc1=O)N1CCN(C[C@@H]1C)C(=O)C=O)-c1ccccc1F	7.41
BDBM548357	CC(C)C[C@@H](Cc1nnc(C)o1)Nc1nc(nc2CC(C)(C)CCc12)N1CCC2(CN(C2)C(=O)C=C)C1	7.41
BDBM544109	CC(C)c1nccc(C)c1-n1c2nc(c(Cl)cc2c(nc1=O)N1C[C@@H](C)N(C[C@@H]1C)C(=O)C=C)-c1cccc(F)c1Cl	7.41
BDBM544108	CC(C)c1nccc(C)c1-n1c2nc(c(Cl)cc2c(nc1=O)N1C[C@@H](C)N(C[C@@H]1C)C(=O)C=C)-c1cccc(F)c1C(F)(F)F	7.41
CHEMBL4861413	Cc1ccnc(C(C)C)c1-n1c(=O)nc(N2CCN(C(=O)C=O)C[C@@H]2C)c2cc(Cl)c(-c3ccccc3F)nc21	7.41
BDBM544437	CC(C)c1nccc(C)c1-n1c2nc(c(Cl)cc2c(nc1=O)N1C[C@@H](C)N(C[C@@H]1C)C(=O)C=C)-c1cc(ccc1F)C#C	7.4
BDBM544089	CC(C)c1nccc(C)c1-n1c2nc(c(Cl)cc2c(nc1=O)N1C[C@@H](C)N(C[C@@H]1C)C(=O)C=C)-c1cc(Cl)ccc1F	7.4
BDBM544070	CNc1ccccc1-c1nc2n(-c3c(C)ccnc3C(C)C)c(=O)nc(N3C[C@@H](C)N(C[C@@H]3C)C(=O)C=C)c2cc1Cl	7.4
BDBM544284	CC(C)c1nccc(C)c1-n1c2nc(c(Cl)cc2c(nc1=O)N1C[C@@H](C)N(C[C@@H]1C)C(=O)C=C)-c1c(F)cccc1NC(=O)C(F)F	7.39
BDBM544062	CC(C)c1nccc(C)c1-n1c2nc(c(Cl)cc2c(nc1=O)N1C[C@@H](C)N(C[C@@H]1C)C(=O)C=C)-c1cccc(F)c1N	7.38

BDBM544083	CC(C)c1nccc(C)c1-n1c2nc(c(Cl)cc2c(nc1=O)N1C[C@@H](C)N(C[C@@H]1C)C(=O)C=C)-c1cc(F)c(F)c(F)c1N	7.38
BDBM544061	CC(C)c1nccc(C)c1-n1c2nc(c(Cl)cc2c(nc1=O)N1C[C@@H](C)N(C[C@@H]1C)C(=O)C=C)-c1ccccc1C(F)F	7.37
BDBM544033	CC(C)c1cccc(CN(C)C)c1-n1c2nc(c(Cl)cc2c(nc1=O)N1CCN(C[C@@H]1C)C(=O)C=C)-c1ccccc1F	7.37
CHEMBL4450657	C=CC(=O)N1CCN(c2nc(=O)n(-c3ccccc3C(C)C)c3nc(-c4c(O)cccc4F)c(Cl)cc23)[C@@H](C)C1	7.36
BDBM544064	CC(C)c1nccc(C)c1-n1c2nc(c(Cl)cc2c(nc1=O)N1C[C@@H](C)N(C[C@@H]1C)C(=O)C=C)-c1ccccc1N(C)C	7.36
BDBM50514378	CC(C)c1ccccc1-n1c2nc(c(Cl)cc2c(nc1=O)N1CCN(C[C@@H]1C)C(=O)C=C)-c1c(O)cccc1F	7.36
BDBM544049	CC(C)c1nccc(C)c1-n1c2nc(c(Cl)cc2c(nc1=O)N1C[C@@H](C)N(C[C@@H]1C)C(=O)C=C)-c1ccccc1C1CC1	7.36
CHEMBL3577120	CNc1cc2nc(C)c(-c3ccc(F)c(NC(=O)NCCC(C)(C)C)c3)cc2cn1	7.34
BDBM544326	CC(C)c1nc(C=C)nc(C(C)C)c1-n1c2nc(c(Cl)cc2c(nc1=O)N1C[C@@H](C)N(C[C@@H]1C)C(=O)C=C)-c1ccccc1F	7.34
BDBM50514387	CC(C)c1ccccc1-n1c2cc(c(Cl)cc2c(nc1=O)N1CCN(C[C@@H]1C)C(=O)C=C)-c1ccccc1F	7.33
CHEMBL4465551	C=CC(=O)N1CCN(c2nc(=O)n(-c3ccccc3C(C)C)c3cc(-c4ccccc4F)c(Cl)cc23)[C@@H](C)C1	7.33
BDBM544082	CC(C)c1nccc(C)c1-n1c2nc(c(Cl)cc2c(nc1=O)N1C[C@@H](C)N(C[C@@H]1C)C(=O)C=C)-c1cc(Br)ccc1F	7.32
BDBM544381	CC(C)c1nc(nc(C)c1-n1c2nc(c(Cl)cc2c(nc1=O)N1C[C@@H](C)N(C[C@@H]1C)C(=O)C=C)-c1ccccc1F)N(C)C	7.32
CHEMBL4456598	C=CC(=O)N1CCN(c2nc(OC[C@@H]3CCCN3C)nc3c2CCN(c2cc(O)cc4ccccc24)C3)CC1	7.32
BDBM544354	CC(C)c1cnc(C)c(C)c1-n1c2nc(c(Cl)cc2c(nc1=O)N1C[C@H](C)N(C[C@@H]1C)C(=O)C=C)-c1ccccc1F	7.32
CHEMBL3577119	CNc1ncc2cc(-c3ccc(F)c(NC(=O)NCCC(C)(C)C)c3)c(C)nc2n1	7.32
BDBM50507447	CN1CCC[C@H]1COc1nc2CN(CCc2c(n1)N1CCN(CC1)C(=O)C=C)c1cc(O)cc2ccccc12	7.32
BDBM544403	CC(C)c1nccc(C)c1-n1c2nc(c(Cl)cc2c(nc1=O)N1C[C@@H](C)N(C[C@@H]1C)C(=O)C=C)-c1ccccc1S(C)=O	7.31
BDBM544336	CCc1cc(C)c(c(n1)C(C)C)-n1c2nc(c(Cl)cc2c(nc1=O)N1C[C@@H](C)N(C[C@@H]1C)C(C)=O)-c1ccccc1F	7.31
BDBM548359	CC(C)C[C@@H](Cc1ncc[nH]1)Nc1nc(nc2CC(C)(C)CCc12)N1CCC2(CN(C2)C(=O)C=C)C1	7.31
BDBM548356	CC(C)C[C@@H](Cc1nnc(C)s1)Nc1nc(nc2CC(C)(C)CCc12)N1CCC2(CN(C2)C(=O)C=C)C1	7.3
BDBM544161	CC(C)c1cncc(C)c1-n1c2nc(c(Cl)cc2c(nc1=O)N1C[C@@H](C)N(C[C@@H]1C)C(=O)C=C)-c1ccccc1F	7.3
BDBM544405	CC(C)c1nccc(C)c1-n1c2nc(c(Cl)cc2c(nc1=O)N1C[C@@H](C)N(C[C@@H]1C)C(=O)C=C)-c1c(F)cccc1S(C)=O	7.3
CHEMBL4467413	C=CC(=O)N1CCN(c2nc(=O)n(-c3ccccc3C(C)(C)C)c3nc(-c4c(O)cccc4F)c(Cl)cc23)[C@@H](C)C1	7.29
BDBM50514369	C[C@H]1CN(CCN1c1nc(=O)n(-c2ccccc2C(C)(C)C)c2nc(c(Cl)cc12)-c1c(O)cccc1F)C(=O)C=C	7.29
BDBM544066	CC(C)c1nccc(C)c1-n1c2nc(c(Cl)cc2c(nc1=O)N1C[C@@H](C)N(C[C@@H]1C)C(=O)C=C)-c1ccccc1C(N)=O	7.29
BDBM544210	CC(C)c1nc(NCCN(C)C)nc(C(C)C)c1-n1c2nc(c(Cl)cc2c(nc1=O)N1C[C@@H](C)N(C[C@@H]1C)C(=O)C=C)-c1ccccc1F	7.29
BDBM50514399	CC(C)c1cccnc1-n1c2nc(c(Cl)cc2c(nc1=O)N1CCN(C[C@@H]1C)C(=O)C=C)-c1c(O)cccc1F	7.28
BDBM50527058	C[C@@H]1CN2[C@@H](COc3cnc4cc(c(Cl)cc4c23)-c2c(O)cccc2F)CN1C(=O)C=C	7.28
BDBM50514381	CC(C)n1ncc(C)c1-n1c2nc(c(Cl)cc2c(nc1=O)N1CCN(C[C@@H]1C)C(=O)C=C)-c1c(O)cccc1F	7.28
CHEMBL4467518	C=CC(=O)N1CCN(c2nc(=O)n(-c3ncccc3C(C)C)c3nc(-c4c(O)cccc4F)c(Cl)cc23)[C@@H](C)C1	7.28
BDBM544288	COc1cc(-c2nc3n(-c4c(C)ccnc4C(C)C)c(=O)nc(N4C[C@@H](C)N(C[C@@H]4C)C(=O)C=C)c3cc2Cl)c2ccccc2c1	7.28
BDBM544141	CC(C)c1nccc(C)c1-n1c2nc(C3CCCCC3)c(Cl)cc2c(nc1=O)N1C[C@@H](C)N(C[C@@H]1C)C(=O)C=C	7.28
BDBM535211	Nc1cc2ccc(F)cc2c(n1)-c1cc2ncnc(N3CCN(CC3)C(=O)C=C)c2cc1Cl	7.28
BDBM544181	CC(C)c1ccccc1-c1nc2n(-c3c(nccc3N(C)C)C(C)C)c(=O)nc(N3C[C@@H](C)N(C[C@@H]3C)C(=O)C=C)c2cc1Cl	7.28
BDBM544278	CCC(=O)Nc1cccc(F)c1-c1nc2n(-c3c(C)ccnc3C(C)C)c(=O)nc(N3C[C@@H](C)N(C[C@@H]3C)C(=O)C=C)c2cc1Cl	7.28
BDBM548353	CC(C)C[C@@H](Cc1ccon1)Nc1nc(nc2CC(C)(C)CCc12)N1CCC2(CN(C2)C(=O)C=C)C1	7.28
BDBM544029	CC(C)c1cccc(CN2CCCC2)c1-n1c2nc(c(Cl)cc2c(nc1=O)N1C[C@@H](C)N(C[C@@H]1C)C(=O)C=C)-c1ccccc1F	7.28
CHEMBL4455191	C=CC(=O)N1C[C@@H]2COc3cnc4cc(-c5c(O)cccc5F)c(Cl)cc4c3N2C[C@H]1C	7.28
BDBM544338	CC(C)c1nc(C)nc(C(C)C)c1-n1c2nc(c(Cl)cc2c(nc1=O)N1C[C@@H](C)N(C[C@@H]1C)C(=O)C=C)-c1ccccc1F	7.28
CHEMBL4516179	C=CC(=O)N1CCN(c2nc(=O)n(-c3c(C)cnn3C(C)C)c3nc(-c4c(O)cccc4F)c(Cl)cc23)[C@@H](C)C1	7.28

BDBM544274	CC(C)c1nccc(C)c1-n1c2nc(c(Cl)cc2c(nc1=O)N1C[C@@H](C)N(C[C@@H]1C)C(=O)C=C)-c1c(F)cccc1NC(C)=O	7.27
BDBM544079	CC(C)c1nccc(C)c1-n1c2nc(c(Cl)cc2c(nc1=O)N1C[C@@H](C)N(C[C@@H]1C)C(=O)C=C)-c1cc(O)ccc1F	7.27
BDBM544088	CC(C)c1nccc(C)c1-n1c2nc(c(Cl)cc2c(nc1=O)N1C[C@@H](C)N(C[C@@H]1C)C(=O)C=C)-c1ccccc1C(C)O	7.27
BDBM544118	CC(C)c1nccc(C)c1-n1c2nc(-c3sccc3C)c(Cl)cc2c(nc1=O)N1C[C@@H](C)N(C[C@@H]1C)C(=O)C=C	7.26
BDBM544365	CC(C)c1ccccc1-n1c2nc(c(Cl)cc2c(nc1=N)N1C[C@@H](C)N(C[C@@H]1C)C(=O)C=C)-c1ccccc1F	7.26
BDBM544418	CC(C)c1cc(cc(C)c1-n1c2nc(c(Cl)cc2c(nc1=O)N1C[C@@H](C)N(C[C@@H]1C)C(=O)C=C)-c1ccccc1F)S(C)(=O)=O	7.26
CHEMBL4591772	C=CC(=O)N1CCN(c2nc(=O)n(-c3ccccc3C(C)C)c3nc(-c4ccccc4Cl)c(Cl)cc23)[C@@H](C)C1	7.25
BDBM544282	COC(=O)Nc1cccc(F)c1-c1nc2n(-c3c(C)ccnc3C(C)C)c(=O)nc(N3C[C@@H](C)N(C[C@@H]3C)C(=O)C=C)c2cc1Cl	7.25
BDBM50514404	CC(C)c1ccccc1-n1c2nc(c(Cl)cc2c(nc1=O)N1CCN(C[C@@H]1C)C(=O)C=C)-c1ccccc1Cl	7.25
BDBM544376	CC(C)c1nc(C)nc(C)c1-n1c2nc(c(Cl)cc2c(nc1=O)N1C[C@@H](C)N(C[C@@H]1C)C(=O)C=C)-c1ccccc1F	7.25
BDBM548360	CC(C)C[C@@H](Cc1c[nH]nn1)Nc1nc(nc2CC(C)(C)CCc12)N1CCC2(CN(C2)C(=O)C=C)C1	7.24
BDBM544185	CC(C)c1cncc(C(C)C)c1-n1c2nc(c(Cl)cc2c(nc1=O)N1C[C@@H](C)N(C[C@@H]1C)C(=O)C=C)-c1ccccc1F	7.24
BDBM544092	CC(C)c1nccc(C)c1-n1c2nc(c(Cl)cc2c(nc1=O)N1C[C@@H](C)N(C[C@@H]1C)C(=O)C=C)-c1ccccc1C#C	7.24
BDBM548334	CNC(=O)C[C@H](CC(C)C)Nc1nc(nc2CC(C)(C)CCc12)N1CCC2(CN(C2)C(=O)C=C)C1	7.24
BDBM548337	CNC(=O)C[C@H](CC(C)C)Nc1nc(nc2CC(CCc12)C(F)(F)F)N1CCC2(CN(C2)C(=O)C=C)C1	7.24
CHEMBL3577114	CNc1cc2c(cn1)cc(-c1ccc(F)c(NC(=O)Nc3cc(C(C)(C)C)no3)c1)c(=O)n2[C@H]1CCOC1	7.24
BDBM544363	CC(C)c1nc(N)cc(C)c1-n1c2nc(c(Cl)cc2c(nc1=O)N1CCN(C[C@@H]1C)C(=O)C=C)-c1ccccc1F	7.24
BDBM535202	CN(C)CCNc1nc(N2CCN(CC2)C(=O)C=C)c2cc(Cl)c(cc2n1)-c1nc(N)ccc1C(F)(F)F	7.23
BDBM544199	CC(C)c1ncccc(C)c1-n1c2nc(c(Cl)nc2c(nc1=O)N1C[C@@H](C)N(C[C@@H]1C)C(=O)C=C)-c1ccccc1F	7.23
BDBM544055	CC(C)c1nccc(C)c1-n1c2nc(c(Cl)cc2c(nc1=O)N1C[C@@H](C)N(C[C@@H]1C)C(=O)C=C)C1=CCCCC1	7.23
BDBM544222	CCc1ncnc(C(C)C)c1-n1c2nc(c(Cl)cc2c(nc1=O)N1C[C@@H](C)N(C[C@@H]1C)C(=O)C=C)-c1ccccc1C	7.23
BDBM544280	CC(C)c1nccc(C)c1-n1c2nc(c(Cl)cc2c(nc1=O)N1C[C@@H](C)N(C[C@@H]1C)C(=O)C=C)-c1ccccc1NC(=O)C1CC1	7.22
BDBM544219	CCc1ncnc(C(C)C)c1-n1c2nc(c(Cl)cc2c(nc1=O)N1C[C@@H](C)N(C[C@@H]1C)C(=O)C=C)-c1ccccc1F	7.22
BDBM515893	CCc1c(C(=O)N2CCc3c(C2)cccc3OC)c2cc(Cl)cc(C)c2n1CC(=O)NC1CN(C1)C(=O)C=C	7.22
BDBM548343	CNC(=O)C[C@H](CC(C)C)Nc1nc(nc2cc(cnc12)C1CC1)N1CCC2(CN(C2)C(=O)C=C)C1	7.22
BDBM544153	CC(C)c1cnn(C(C)C)c1-n1c2nc(c(Cl)cc2c(nc1=O)N1C[C@@H](C)N(C[C@@H]1C)C(=O)C=C)-c1ccccc1F	7.21
BDBM544305	CC(C)c1cccc2C\C=C/Cc3ccccc3-c3nc4n(-c12)c(=O)nc(N1C[C@@H](C)N(C[C@@H]1C)C(=O)C=C)c4cc3Cl	7.21
BDBM544044	CC(C)c1cc(CN(C)C)cc(C)c1-n1c2nc(c(F)cc2c(nc1=O)N1C[C@@H](C)N(C[C@@H]1C)C(=O)C=C)-c1c(O)cccc1F	7.21
BDBM544036	CC(C)c1nccc(CN(C)C)c1-n1c2nc(c(Cl)cc2c(nc1=O)N1C[C@@H](C)N(C[C@@H]1C)C(=O)C=C)-c1ccccc1F	7.21
BDBM544034	CC(C)c1cccc(CN(C)C)c1-n1c2nc(c(Cl)cc2c(nc1=O)N1C[C@@H](C)N(C[C@@H]1C)C(=O)C=C)-c1ccccc1F	7.21
BDBM535186	Nc1ccc(c(n1)-c1cc2ncnc(N3CCN(CC3)C(=O)C=C)c2cc1Cl)C(F)(F)F	7.21
BDBM544321	COc1nc(C(C)C)c(c(n1)C(C)C)-n1c2nc(c(Cl)cc2c(nc1=O)N1C[C@@H](C)N(C[C@@H]1C)C(=O)C=C)-c1ccccc1F	7.21
BDBM50514392	CC(C)c1ncsc1-n1c2nc(c(Cl)cc2c(nc1=O)N1CCN(C[C@@H]1C)C(=O)C=C)-c1c(O)cccc1F	7.2
CHEMBL4573279	C=CC(=O)N1CCN(c2nc(=O)n(-c3scnc3C(C)C)c3nc(-c4c(O)cccc4F)c(Cl)cc23)[C@@H](C)C1	7.2
CHEMBL4640234	C=CC(=O)N1CCN(c2nc(OC[C@@H]3CCCN3C)nc3c2CCN(c2cccc4cccc(C#N)c24)C3)C[C@@H]1CC#N	7.19
BDBM535149	Cc1cc(N)nc(c1Cl)-c1cc2ncnc(N3CCN(CC3)C(=O)C=C)c2cc1Cl	7.19
BDBM544281	COC(=O)Nc1ccccc1-c1nc2n(-c3c(C)ccnc3C(C)C)c(=O)nc(N3C[C@@H](C)N(C[C@@H]3C)C(=O)C=C)c2cc1Cl	7.19
BDBM544032	CC(C)c1cccc(CN2CCC2)c1-n1c2nc(c(Cl)cc2c(nc1=O)N1C[C@@H](C)N(C[C@@H]1C)C(=O)C=C)-c1ccccc1F	7.19
BDBM544341	CC(C)c1nc(CN2CCN(C)CC2)nc(C(C)C)c1-n1c2nc(c(Cl)cc2c(nc1=O)N1C[C@@H](C)N(C[C@@H]1C)C(=O)C=C)-c1ccccc1F	7.19
BDBM544324	CC(C)c1nc(OCCN(C)C)nc(C(C)C)c1-n1c2nc(c(Cl)cc2c(nc1=O)N1C[C@@H](C)N(C[C@@H]1C)C(=O)C=C)-c1ccccc1F	7.19
CHEMBL4578214	C=CC(=O)N1CCN(c2nc(=O)n(-c3c(C(C)C)ccnc3C)c3nc(-c4c(O)cccc4F)c(Cl)cc23)[C@@H](C)C1	7.19

BDBM544276	CC(C)c1nccc(C)c1-n1c2nc(c(Cl)cc2c(nc1=O)N1C[C@@H](C)N(C[C@@H]1C)C(=O)C=C)-c1c(F)cccc1NC(=O)C(F)(F)F	7.19
CHEMBL4873951	Cc1ccnc(C(C)C)c1-n1c(=O)nc(N2CCN(C(=O)[C@@H]3O[C@H]3CO)C[C@@H]2C)c2cc(Cl)c(-c3ccccc3F)nc21	7.19
BDBM50514394	CC(C)c1ccnc(C)c1-n1c2nc(c(Cl)cc2c(nc1=O)N1CCN(C[C@@H]1C)C(=O)C=C)-c1c(O)cccc1F	7.19
BDBM50573428	CC(C)c1nccc(C)c1-n1c2nc(c(Cl)cc2c(nc1=O)N1CCN(C[C@@H]1C)C(=O)[C@@H]1O[C@H]1CO)-c1ccccc1F	7.19
BDBM50539758	CN1CCC[C@H]1COc1nc2CN(CCc2c(n1)N1CCN([C@@H](CC#N)C1)C(=O)C=C)c1cccc2cccc(C#N)c12	7.19
BDBM544110	CC(C)c1nccc(C)c1-n1c2nc(c(Cl)cc2c(nc1=O)N1C[C@@H](C)N(C[C@@H]1C)C(=O)C=C)-c1cc(F)ccc1F	7.18
BDBM544230	CC(C)c1cc(cc(C)c1-n1c2nc(c(Cl)cc2c(nc1=O)N1C[C@@H](C)N(C[C@@H]1C)C(=O)C=C)-c1ccccc1F)N(C)C	7.18
BDBM544332	CC(C)c1nc(nc(C)c1-n1c2nc(c(Cl)cc2c(nc1=O)N1C[C@@H](C)N(C[C@@H]1C)C(=O)C=C)-c1ccccc1F)C1CC1	7.18
BDBM50514390	CC(C)c1ccccc1-n1c2nc(c(Cl)cc2c(nc1=O)N1CCN(C[C@@H]1C)C(=O)C=C)-c1ccccc1F	7.18
BDBM544390	CC(C)c1nc(nc(C)c1-n1c2nc(c(Cl)cc2c(nc1=O)N1C[C@@H](C)N(C[C@@H]1C)C(=O)C=C)-c1ccccc1F)C#C[Si](C)(C)C	7.18
BDBM544173	CC(C)c1ncnc(Cl)c1-n1c2nc(c(Cl)cc2c(nc1=O)N1C[C@@H](C)N(C[C@@H]1C)C(=O)C=C)-c1ccccc1C	7.18
CHEMBL4450519	C=CC(=O)N1CCN(c2nc(=O)n(-c3ccccc3C(C)C)c3nc(-c4ccccc4F)c(Cl)cc23)[C@@H](C)C1	7.18
BDBM544412	CC(C)c1cc(cc(C)c1-n1c2nc(c(Cl)cc2c(nc1=O)N1C[C@@H](C)N(C[C@@H]1C)C(=O)C=C)-c1ccccc1C)S(C)=O	7.18
BDBM544138	CC(C)c1nccc(C)c1-n1c2nc(c(F)cc2c(nc1=O)N1C[C@@H](C)N(C[C@@H]1C)C(=O)C=C)-c1c(F)cccc1C(N)=O	7.17
BDBM544258	CC(C)c1nccc(C)c1-n1c2nc(c(Cl)cc2c(nc1=O)N1C[C@@H](C)N(C[C@@H]1C)C(=O)C=C)-c1cc(F)c(F)cc1O	7.17
BDBM544287	CC(C)c1nccc(C)c1-n1c2nc(c(Cl)cc2c(nc1=O)N1C[C@@H](C)N(C[C@@H]1C)C(=O)C=C)-c1c(F)cccc1NCC#C	7.17
BDBM544377	CC(C)c1ncnc(C)c1-n1c2nc(c(Cl)cc2c(nc1=O)N1C[C@@H](C)N(C[C@@H]1C)C(=O)C=C)-c1ccccc1F	7.17
CHEMBL4439782	C=CC(=O)N1CCN(c2nc(=O)n(-c3ccccc3C(C)C)c3nc(-c4c(O)cccc4F)c(Cl)cc23)CC1	7.16
BDBM50514389	CC(C)c1ccccc1-n1c2nc(c(Cl)cc2c(nc1=O)N1CCN(CC1)C(=O)C=C)-c1c(O)cccc1F	7.16
BDBM544346	COc1ccc(C)c(c1)-c1nc2n(-c3c(ncnc3C(C)C)C(C)C)c(=O)nc(N3C[C@@H](C)N(BC=O)C[C@@H]3C)c2cc1Cl	7.16
BDBM544356	CC(C)c1cnc(C)c(C)c1-n1c2nc(c(Cl)cc2c(nc1=O)N1C[C@@H](C)N(C[C@@H]1C)C(=O)C=C)-c1ccccc1F	7.16
CHEMBL4572730	C=CC(=O)N1CCN2c3c(cnc4c(OC)c(-c5c(O)cccc5F)c(Cl)cc34)OC[C@H]2C1	7.15
BDBM535180	Nc1ccc(c(n1)-c1cc2ncnc(N3CCN(CC3)C(=O)C=C)c2cc1C1CC1)C(F)(F)F	7.15
BDBM544388	CC(C)c1nc(C=C)nc(C)c1-n1c2nc(c(Cl)cc2c(nc1=O)N1C[C@@H](C)N(C[C@@H]1C)C(=O)C=C)-c1ccccc1F	7.15
BDBM544166	CC(C)c1cccc(C(C)C)c1-n1c2nc(c(Cl)cc2c(nc1=O)N1C[C@@H](C)N(C[C@@H]1C)C(=O)C=C)-c1ccccc1F	7.15
BDBM50527050	COc1c(c(Cl)cc2c3N4CCN(C[C@@H]4COc3cnc12)C(=O)C=C)-c1c(O)cccc1F	7.15
BDBM544440	CC(C)c1nc(N)nc(C(C)C)c1-n1c2nc(c(Cl)cc2c(nc1=O)N1C[C@@H](C)N(C[C@@H]1C)C(=O)C=C)-c1ccccc1F	7.15
BDBM543961	CC(C)c1nccc(N(C)C)c1-n1c2nc(c(Cl)cc2c(nc1=O)N1C[C@@H](C)N(C[C@@H]1C)C(=O)C=C)-c1ccccc1F	7.15
BDBM515791	CCc1c(C(=O)OC)c2cc(Cl)cc(C)c2n1CC(=O)NC1CN(C1)C(=O)C=C	7.15
BDBM544348	CC(C)c1nc(cc(C)c1-n1c2nc(c(Cl)cc2c(nc1=O)N1C[C@H](C)N(C[C@@H]1C)C(=O)C=C)-c1ccccc1F)N1CCC1	7.15
BDBM544334	CC(C)c1nc(C)cc(C)c1-n1c2nc(c(Cl)cc2c(nc1=O)N1C[C@@H](C)N(C[C@@H]1C)C(=O)C=C)-c1ccccc1F	7.14
BDBM50527054	Oc1cccc(F)c1-c1cc2ncc3NC(=O)[C@H]4CN(CCN4c3c2cc1Cl)C(=O)C=C	7.14
CHEMBL4453777	C=CC(=O)N1CCN2c3c(cnc4cc(-c5c(O)cccc5F)c(Cl)cc34)NC(=O)[C@H]2C1	7.14
BDBM544103	CC(C)c1nccc(C)c1-n1c2nc(c(Cl)cc2c(nc1=O)N1C[C@@H](C)N(C[C@@H]1C)C(=O)C=C)-c1cccc(C)c1F	7.13
BDBM544255	CC(C)c1ccnc(C(C)C)c1-n1c2nc(c(Cl)cc2c(nc1=O)N1C[C@H](C)N([C@H](C)C1)C(=O)C=C)-c1ccccc1C	7.12
BDBM544387	CC(C)c1nc(nc(C)c1-n1c2nc(c(Cl)cc2c(nc1=O)N1C[C@@H](C)N(C[C@@H]1C)C(=O)C=C)-c1ccccc1F)C(C)=C	7.12
BDBM544077	CC(C)c1nccc(C)c1-n1c2nc(c(Cl)cc2c(nc1=O)N1C[C@@H](C)N(C[C@@H]1C)C(=O)C=C)-c1ccccc1NS(C)(=O)=O	7.12
BDBM544120	CC(C)c1ccccc1-c1nc2n(-c3c(C)ccnc3C(C)C)c(=O)nc(N3C[C@@H](C)N(C[C@@H]3C)C(=O)C=C)c2cc1F	7.11
BDBM544031	CC(C)c1cccc(CN2CC(F)(F)C2)c1-n1c2nc(c(Cl)cc2c(nc1=O)N1C[C@@H](C)N(C[C@@H]1C)C(=O)C=C)-c1ccccc1F	7.11
BDBM544391	CC(C)c1cnnc(C(C)C)c1-n1c2nc(c(Cl)cc2c(nc1=O)N1C[C@@H](C)N(C[C@@H]1C)C(=O)C=C)-c1ccccc1F	7.11
BDBM535509	C[C@H]1CN(CCN1c1nc(OC[C@@H]2CCCN2)nc2c(F)c(c(Cl)cc12)-c1nc(N)cc(C)c1C(F)(F)F)C(=O)C=C	7.1

CHEMBL4469638	C=CC(=O)N1CCN(c2nc(=O)n(-c3ccccc3C(C)C)c3cc(-c4ccccc4F)c(Cl)cc23)CC1	7.1
BDBM544169	CC(C)c1cc(Br)nc(C(C)C)c1-n1c2nc(c(Cl)cc2c(nc1=O)N1C[C@@H](C)N(C[C@@H]1C)C(=O)C=C)-c1ccccc1F	7.1
BDBM544373	CC(C)c1nc(Cl)nc(C)c1-n1c2nc(c(Cl)cc2c(nc1=O)N1C[C@@H](C)N(C[C@@H]1C)C(=O)C=C)-c1ccccc1F	7.1
BDBM544343	CC(C)c1ncnc(C(C)C)c1-n1c2nc(c(Cl)cc2c(nc1=O)N1C[C@@H](C)N(C[C@@H]1C)C(=O)C=C)-c1ccccc1C(F)(F)F	7.1
BDBM50514368	CC(C)c1ccccc1-n1c2cc(c(Cl)cc2c(nc1=O)N1CCN(CC1)C(=O)C=C)-c1ccccc1F	7.1
BDBM515896	COc1cccc2CN(CCc12)C(=O)c1c(CC2CC2)n(CC(=O)NC2CN(C2)C(=O)C=C)c2c(C)cc(Cl)cc12	7.1
BDBM544193	CC(C)c1nc(Br)nc(C(C)C)c1-n1c2nc(c(Cl)cc2c(nc1=O)N1C[C@@H](C)N(C[C@@H]1C)C(=O)C=C)-c1ccccc1F	7.1
BDBM544187	CCN(CC)c1ccnc(C(C)C)c1-n1c2nc(c(Cl)cc2c(nc1=O)N1C[C@@H](C)N(C[C@@H]1C)C(=O)C=C)-c1ccccc1C	7.1
CHEMBL4483782	C=CC(=O)N1CCN2c3c(cnc4cc(-c5c(C)ccc6[nH]ncc56)c(Cl)cc34)OC[C@H]2C1	7.09
BDBM50527052	Cc1ccc2[nH]ncc2c1-c1cc2ncc3OC[C@H]4CN(CCN4c3c2cc1Cl)C(=O)C=C	7.09
BDBM544328	CC(C)c1nc(CN(C)C)nc(C(C)C)c1-n1c2nc(c(Cl)cc2c(nc1=O)N1C[C@@H](C)N(C[C@@H]1C)C(=O)C=C)-c1ccccc1F	7.09
BDBM548323	CC(C)C[C@@H](Cc1nnc[nH]1)Nc1nc(nc2cc(ccc12)C(C)C)N1CCC2(CN(C2)C(=O)C=C)C1	7.08
BDBM544183	CC(C)c1ncn(C)c(=O)c1-n1c2nc(c(Cl)cc2c(nc1=O)N1C[C@@H](C)N(C[C@@H]1C)C(=O)C=C)-c1ccccc1F	7.08
BDBM534957	Nc1cc2ccccc2c(n1)-c1cc2ncnc(N3CCN(CC3)C(=O)C=C)c2cc1Cl	7.08
BDBM535304	C[C@@H]1CN(CCN1c1nc(OC[C@@H]2CCCN2C)nc2c(F)c(c(SC(F)(F)F)cc12)-c1nc(N)cc(C)c1C(F)(F)F)C(=O)C=C	7.08
BDBM548345	CNC(=O)C[C@H](CC(C)C)Nc1nc(nc2cc(ccc12)C1CC1)N1CCC2(CN(C2)C(=O)C=C)C1	7.08
BDBM544269	CC(C)c1ccnc(C(C)C)c1-n1c2nc(c(Cl)cc2c(nc1=O)N1C[C@@H](C)N(C[C@@H]1C)C(=O)C=C)-c1ccccc1	7.07
BDBM544063	CC(C)c1nccc(C)c1-n1c2nc(c(Cl)cc2c(nc1=O)N1C[C@@H](C)N(C[C@@H]1C)C(=O)C=C)-c1ccccc1NC(C)=O	7.07
BDBM544234	CCC(=O)Nc1cccc(F)c1-c1nc2n(-c3c(nccc3N(C)C)C(C)C)c(=O)nc(N3C[C@@H](C)N(C[C@@H]3C)C(=O)C=C)c2cc1Cl	7.06
BDBM544192	CC(C)c1nccc(N(C)C)c1-n1c2nc(c(Cl)cc2c(nc1=O)N1C[C@@H](C)N(C[C@@H]1C)C(=O)C=C)-c1ccccc1C	7.06
BDBM544400	CC(C)c1cc(ccc1-n1c2nc(c(Cl)cc2c(nc1=O)N1C[C@@H](C)N(C[C@@H]1C)C(=O)C=C)-c1ccccc1F)S(C)=O	7.06
BDBM50514396	CC(C)c1cccc(C)c1-n1c2nc(c(F)cc2c(nc1=O)N1CCN(C[C@@H]1C)C(=O)C=C)-c1c(O)cccc1F	7.05
CHEMBL4452622	C=CC(=O)N1CCN(c2nc(=O)n(-c3c(C)cccc3C(C)C)c3nc(-c4c(O)cccc4F)c(F)cc23)[C@@H](C)C1	7.05
BDBM543955	CC(C)c1nccc(Cl)c1-n1c2nc(c(Cl)cc2c(nc1=O)N1C[C@@H](C)N(C[C@@H]1C)C(=O)C=C)-c1ccccc1F	7.05
BDBM515935	Cc1c(C(=O)N2CCc3c(C)cccc3C2)c2cc(Br)ccc2n1CC(=O)NC1CN(C1)C(=O)C=C	7.05
BDBM515910	COc1cccc2CN(CCc12)C(=O)c1c(C)n(CC(=O)N(C)C2CN(C2)C(=O)C=C)c2c(C)cc(Br)cc12	7.05
CHEMBL3577115	CCn1c(=O)c(-c2ccc(F)c(NC(=O)Nc3cc(C(C)(C)C)no3)c2)cc2cnc(NC)cc21	7.04
BDBM544423	CC(C)c1nccc(C)c1N1CC(=O)N=C(N2C[C@@H](C)N(C[C@@H]2C)C(=O)C=C)c2cc(Cl)c(nc12)-c1ccccc1F	7.03
BDBM544050	CC(C)c1nccc(C)c1-n1c2nc(-c3cscc3C)c(Cl)cc2c(nc1=O)N1C[C@@H](C)N(C[C@@H]1C)C(=O)C=C	7.03
BDBM544060	CC(C)c1nccc(C)c1-n1c2nc(c(Cl)cc2c(nc1=O)N1C[C@@H](C)N(C[C@@H]1C)C(=O)C=C)-c1c(C)ccc2cn[nH]c12	7.03
BDBM544366	CC(C)c1ccccc1-n1c2nc(c(Cl)cc2c(nc1=O)N1C[C@@H](C)N(C[C@@H]1C)C(=O)C=C)-c1ccccc1F	7.03
BDBM548354	CC(C)C[C@@H](Cc1ccno1)Nc1nc(nc2CC(C)(C)CCc12)N1CCC2(CN(C2)C(=O)C=C)C1	7.03
BDBM50573425	CC(C)c1nccc(C)c1-n1c2nc(c(Cl)cc2c(nc1=O)N1CCN(C[C@@H]1C)C(=O)C1OC1C)-c1ccccc1F	7.02
BDBM544339	CC(C)c1nc(nc(C(C)C)c1-n1c2nc(c(Cl)cc2c(nc1=O)N1C[C@@H](C)N(C[C@@H]1C)C(=O)C=C)-c1ccccc1F)C1CC1	7.02
BDBM544229	CC(C)c1nc(CN2CCCC2)cc(C)c1-n1c2nc(c(Cl)cc2c(nc1=O)N1C[C@@H](C)N(C[C@@H]1C)C(=O)C=C)-c1ccccc1F	7.02
BDBM544048	CC(C)c1nccc(C)c1-n1c2nc(-c3ccsc3C)c(Cl)cc2c(nc1=O)N1C[C@@H](C)N(C[C@@H]1C)C(=O)C=C	7.02
CHEMBL4877899	Cc1ccnc(C(C)C)c1-n1c(=O)nc(N2CCN(C(=O)C3OC3C)C[C@@H]2C)c2cc(Cl)c(-c3ccccc3F)nc21	7.02
CHEMBL4455938	C=CC(=O)N1CCN2c3ncnc4cc(-c5c(O)cccc5F)c(Cl)c(c34)OC[C@@H]2C1	7.01
BDBM50527046	Cc1c(c(Cl)cc2c3N4CCN(C[C@@H]4COc3cnc12)C(=O)C=C)-c1c(O)cccc1F	7.01
BDBM50521255	Oc1cccc(F)c1-c1cc2ncnc3N4CCN(C[C@H]4COc(c1Cl)c23)C(=O)C=C	7.01
BDBM544208	CC(C)c1nc(nc(C(C)C)c1-n1c2nc(c(Cl)cc2c(nc1=O)N1C[C@@H](C)N(C[C@@H]1C)C(=O)C=C)-c1ccccc1F)N(C)C	7.01

CHEMBL4567284	C=CC(=O)N1CCN2c3c(cnc4c(C)c(-c5c(O)cccc5F)c(Cl)cc34)OC[C@H]2C1	7.01
BDBM544394	CC(C)c1cnnc(C(C)C)c1-n1c2nc(c(Cl)cc2c(nc1=O)N1C[C@H](C)N([C@H](C)C1)C(=O)C=C)-c1ccccc1F	7.01
CHEMBL4517656	C=CC(=O)N1CCN(c2nc(=O)n(-c3ccccc3C(C)C)c3cc(-c4c(O)cccc4F)c(Cl)cc23)CC1	7
BDBM515909	COc1cccc2CN(CCc12)C(=O)c1c(CC2CC2)n(CC(=O)N(C)C2CN(C2)C(=O)C=C)c2c(C)cc(Cl)cc12	7
BDBM515976	CN(C1CN(C1)C(=O)C=C)C(=O)Cn1cc(I)c2cc(I)ccc12	7
BDBM515931	CN(C1CN(C1)C(=O)C=C)C(=O)Cn1c(C)c(C(=O)N2CCN(CC2)c2ccc(Br)cn2)c2cc(Br)ccc12	7
BDBM50517182	COc1cccc2CN(CCc12)C(=O)c1c(C)n(CC(=O)NC2CN(C2)C(=O)C=C)c2c(C)cc(Cl)cc12	7
BDBM544071	CC(C)c1nccc(C)c1-n1c2nc(c(Cl)cc2c(nc1=O)N1C[C@@H](C)N(C[C@@H]1C)C(=O)C=C)-c1cc(F)cc(F)c1N	7
BDBM50514377	CC(C)c1ccccc1-n1c2cc(c(Cl)cc2c(nc1=O)N1CCN(CC1)C(=O)C=C)-c1c(O)cccc1F	7
BDBM544211	CC(C)c1nc(nc(C(C)C)c1-n1c2nc(c(Cl)cc2c(nc1=O)N1C[C@@H](C)N(C[C@@H]1C)C(=O)C=C)-c1ccccc1F)N1CCC1	6.99
BDBM544322	CC(C)c1nc(nc(C(C)C)c1-n1c2nc(c(Cl)cc2c(nc1=O)N1C[C@@H](C)N(C[C@@H]1C)C(=O)C=C)-c1ccccc1F)C#N	6.98
BDBM50517187	COc1cccc2CN(CCc12)C(=O)c1c(C2CC2)n(CC(=O)NC2CN(C2)C(=O)C=C)c2c(C)cc(Cl)cc12	6.96
BDBM515899	COC(=O)c1c(C)n(CC(=O)NC2CN(C2)C(=O)C=C)c2c(C)cc(Br)cc12	6.96
BDBM50517179	COc1cccc2CN(CCc12)C(=O)c1c(C)n(CC(=O)NC2CN(C2)C(=O)C=C)c2ccc(Br)cc12	6.96
BDBM544038	CC(C)c1ncnc(CN(C)C)c1-n1c2nc(c(Cl)cc2c(nc1=O)N1C[C@@H](C)N(C[C@@H]1C)C(=O)C=C)-c1ccccc1F	6.96
BDBM515968	Cc1c(C(=O)N2CCc3c(O)cccc3C2)c2cc(Br)ccc2n1CC(=O)NC1CN(C1)C(=O)C=C	6.96
BDBM516036	COc1cccc2CN(CCc12)C(=O)c1c(C)n(C(C)C(=O)NC2CN(C2)C(=O)C=C)c2ccc(Cl)cc12	6.96
CHEMBL4549665	C=CC(=O)N1CCN(c2nc(=O)n(-c3ccccc3C3CC3)c3nc(-c4c(O)cccc4F)c(Cl)cc23)[C@@H](C)C1	6.96
CHEMBL4541628	C=CC(=O)N1CC(NC(=O)Cn2c(C)c(C(=O)N3CCc4c(cccc4OC)C3)c3cc(Br)cc(C)c32)C1	6.96
BDBM50514401	C[C@H]1CN(CCN1c1nc(=O)n(-c2ccccc2C2CC2)c2nc(c(Cl)cc12)-c1c(O)cccc1F)C(=O)C=C	6.96
BDBM50517181	COc1cccc2CN(CCc12)C(=O)c1c(C)n(CC(=O)NC2CN(C2)C(=O)C=C)c2c(C)cc(Br)cc12	6.96
BDBM544188	CCN(CC)c1ccnc(C(C)C)c1-n1c2nc(c(Cl)cc2c(nc1=O)N1C[C@@H](C)N(C[C@@H]1C)C(=O)C=C)-c1ccccc1C(C)C	6.95
BDBM544078	CC(C)c1nccc(C)c1-n1c2nc(c(Cl)cc2c(nc1=O)N1C[C@@H](C)N(C[C@@H]1C)C(=O)C=C)-c1ccccc1S(N)(=O)=O	6.95
BDBM50517238	C[C@@H]1Cc2c(C[C@H]1c1c(Cl)c(C)cc3[nH]ncc13)nc(nc2N1CCN(CC1)C(=O)C=C)N1CC(C1)N(C)C	6.94
CHEMBL4457820	C=CC(=O)N1CCN(c2nc(N3CC(N(C)C)C3)nc3c2C[C@@H](C)[C@H](c2c(Cl)c(C)cc4[nH]ncc24)C3)CC1	6.94
CHEMBL4464335	C=CC(=O)N1CC(NC(=O)Cn2c(C3CC3)c(C(=O)N3CCc4c(cccc4OC)C3)c3cc(Cl)cc(C)c32)C1	6.94
BDBM544209	CC(C)c1nc(nc(C(C)C)c1-n1c2nc(c(Cl)cc2c(nc1=O)N1C[C@@H](C)N(C[C@@H]1C)C(=O)C=C)-c1ccccc1F)N1CCOCC1	6.94
BDBM544087	CC(C)c1nccc(C)c1-n1c2nc(c(Cl)cc2c(nc1=O)N1C[C@@H](C)N(C[C@@H]1C)C(=O)C=C)-c1ccccc1	6.93
BDBM544086	CC(C)c1nccc(C)c1-n1c2nc(c(Cl)cc2c(nc1=O)N1C[C@@H](C)N(C[C@@H]1C)C(=O)C=C)-c1ccccc1C(C)=O	6.93
BDBM50459706	Oc1cccc(F)c1-c1c(Cl)cc2c(ncnc2c1F)N1CCN(CC1)C(=O)C=C	6.92
BDBM515908	COc1cccc2CN(CCc12)C(=O)c1c(C2CC2)n(CC(=O)N(C)C2CN(C2)C(=O)C=C)c2c(C)cc(Cl)cc12	6.92
CHEMBL4214264	C=CC(=O)N1CCN(c2ncnc3c(F)c(-c4c(O)cccc4F)c(Cl)cc23)CC1	6.92
CHEMBL3577121	CNc1cc2nc(C)c(-c3cc(NC(=O)NCCC(C)(C)C)c(F)cc3C)cc2cn1	6.92
CHEMBL3577116	CCn1c(=O)c(-c2cc(NC(=O)NCCC(C)(C)C)c(F)cc2C)cc2cnc(NC)cc21	6.92
BDBM515906	CCc1c(C(=O)N2CCc3c(C2)ccnc3OC)c2cc(Cl)cc(C)c2n1CC(=O)N(C)C1CN(C1)C(=O)C=C	6.92
BDBM50517189	COc1cccc2CN(CCc12)C(=O)c1c(C)n(CC(=O)NC2CN(C2)C(=O)C=C)c2ccc(Cl)cc12	6.92
BDBM548348	CC(C)C[C@@H](Cc1nccs1)Nc1nc(nc2CC(C)(C)CCc12)N1CCC2(CN(C2)C(=O)C=C)C1	6.92
BDBM544059	CC(C)c1nccc(C)c1-n1c2nc(c(Cl)cc2c(nc1=O)N1C[C@@H](C)N(C[C@@H]1C)C(=O)C=C)-c1ccccc1OC(F)F	6.92
BDBM544094	CC#Cc1cccc(c1)-c1nc2n(-c3c(C)ccnc3C(C)C)c(=O)nc(N3C[C@@H](C)N(C[C@@H]3C)C(=O)C=C)c2cc1Cl	6.91
BDBM544073	CC(C)c1nccc(C)c1-n1c2nc(c(Cl)cc2c(nc1=O)N1C[C@@H](C)N(C[C@@H]1C)C(=O)C=C)-c1ccccc1C(=O)N(C)C	6.91
BDBM544102	CC(C)c1nccc(C)c1-n1c2nc(c(Cl)cc2c(nc1=O)N1C[C@@H](C)N(C[C@@H]1C)C(=O)C=C)-c1ccc(F)cc1	6.9

BDBM544331	CC(C)c1nc(CN2CCCC2)nc(C(C)C)c1-n1c2nc(c(Cl)cc2c(nc1=O)N1C[C@@H](C)N(C[C@@H]1C)C(=O)C=C)-c1ccccc1F	6.9
CHEMBL4473733	C=CC(=O)N1CCN(c2nc(OC[C@H]3CCCN3C)nc3c2C[C@@H](C)[C@H](c2c(C)ccc4[nH]ncc24)C3)CC1	6.89
BDBM544202	CC(C)c1nccc(C)c1-n1c2nc(c(Cl)nc2c(nc1=O)N1[C@@H](C)CN(C[C@H]1C)C(=O)C=C)-c1ccccc1F	6.89
CHEMBL4441771	C=CC(=O)N1CCN(c2nc(=O)n(-c3c(C)ncnc3C(C)C)c3nc(-c4c(O)cccc4F)c(F)cc23)[C@@H](C)C1	6.89
BDBM544344	CC(C)c1nc(cc(C)c1-n1c2nc(c(Cl)cc2c(nc1=O)N1C[C@@H](C)N(C[C@@H]1C)C(=O)C=C)-c1ccccc1F)C(C)=C	6.89
BDBM515979	COc1cccc2CN(CCc12)C(=O)c1cn(CC(=O)NC2CN(C2)C(=O)C=C)c2c(Cl)cc(Cl)cc12	6.89
BDBM50517180	COc1cccc2CN(CCc12)C(=O)c1cn(CC(=O)NC2CN(C2)C(=O)C=C)c2c(C)cc(Br)cc12	6.89
BDBM544112	COc1ccccc1-c1nc2n(-c3c(C)ccnc3C(C)C)c(=O)nc(N3C[C@@H](C)N(C[C@@H]3C)C(=O)C=C)c2cc1Cl	6.89
BDBM516004	Cc1cccc2CN(CCc12)C(=O)c1cn(CC(=O)NC2CN(C2)C(=O)C=C)c2c(C)cc(Br)cc12	6.89
BDBM515943	COc1cccc2CN(CCc12)C(=O)c1c(C)n(CC(=O)N(C)C2CN(C2)C(=O)C=C)c2ccc(Cl)cc12	6.89
BDBM515911	CCc1c(C(=O)N2CCN(C[C@H]2C)c2ccc(cn2)C#N)c2cc(Cl)cc(C)c2n1CC(=O)N(C)C1CN(C1)C(=O)C=C	6.89
BDBM50514397	CC(C)c1ncnc(C)c1-n1c2nc(c(F)cc2c(nc1=O)N1CCN(C[C@@H]1C)C(=O)C=C)-c1c(O)cccc1F	6.89
BDBM50517237	C[C@@H]1Cc2c(C[C@H]1c1c(C)ccc3[nH]ncc13)nc(OC[C@H]1CCCN1C)nc2N1CCN(CC1)C(=O)C=C	6.89
BDBM544330	CC(C)c1nc(CN2CCOCC2)nc(C(C)C)c1-n1c2nc(c(Cl)cc2c(nc1=O)N1C[C@@H](C)N(C[C@@H]1C)C(=O)C=C)-c1ccccc1F	6.88
BDBM544385	CCc1nc(C)c(c(n1)C(C)C)-n1c2nc(c(Cl)cc2c(nc1=O)N1C[C@@H](C)N(C[C@@H]1C)C(=O)C=C)-c1ccccc1F	6.88
CHEMBL4461913	C=CC(=O)N1CCN2c3c(cnc4cc(-c5c(O)cccc5F)c(Cl)cc34)N(C)C[C@H]2C1	6.88
BDBM50527055	CN1C[C@H]2CN(CCN2c2c1cnc1cc(c(Cl)cc21)-c1c(O)cccc1F)C(=O)C=C	6.88
BDBM544349	CC(C)c1cc(C)c(c(n1)C(C)C)-n1c2nc(c(Cl)cc2c(nc1=O)N1C[C@@H](C)N(C[C@@H]1C)C(=O)C=C)-c1ccccc1F	6.88
BDBM544098	CC(C)c1nccc(C)c1-n1c2nc(c(Cl)cc2c(nc1=O)N1C[C@@H](C)N(C[C@@H]1C)C(=O)C=C)-c1cccc(C)c1	6.87
BDBM544163	CC(C)c1cncc(C(C)C)c1-n1c2nc(c(F)cc2c(nc1=O)N1C[C@@H](C)N(C[C@@H]1C)C(=O)C=C)-c1c(O)cccc1F	6.87
BDBM544129	CC(C)c1nccc(C)c1-n1c2nc(c(F)cc2c(nc1=O)N1C[C@@H](C)N(C[C@@H]1C)C(=O)C=C)-c1c(C)cccc1F	6.86
BDBM50517173	COc1cccc2CN(CCc12)C(=O)c1c(C2CC2)n(CC(=O)NC2CN(C2)C(=O)C=C)c2ccc(Br)cc12	6.86
CHEMBL4579956	C=CC(=O)N1CC(NC(=O)Cn2c(C3CC3)c(C(=O)N3CCc4c(cccc4OC)C3)c3cc(Br)ccc32)C1	6.86
BDBM544074	CC(C)c1nccc(C)c1-n1c2nc(c(Cl)cc2c(nc1=O)N1C[C@@H](C)N(C[C@@H]1C)C(=O)C=C)-c1ccccc1C(O)=O	6.86
BDBM50514374	CC(C)c1ccnc(C)c1-n1c2nc(c(F)cc2c(nc1=O)N1CCN(C[C@@H]1C)C(=O)C=C)-c1c(O)cccc1F	6.85
BDBM544359	CC(C)c1cc(nc(C(C)C)c1-n1c2nc(c(Cl)cc2c(nc1=O)N1C[C@@H](C)N(C[C@@H]1C)C(=O)C=C)-c1ccccc1F)C1CCC1	6.85
BDBM548340	CNC(=O)C[C@H](CC(C)C)Nc1nc(nc2cc(ccc12)C(F)(F)F)N1CCC2(CN(C2)C(=O)C=C)C1	6.85
CHEMBL4458041	C=CC(=O)N1CCN(c2nc(=O)n(-c3c(C(C)C)ccnc3C)c3nc(-c4c(O)cccc4F)c(F)cc23)[C@@H](C)C1	6.85
BDBM515967	Brc1ccc2n(CC(=O)NC3CN(C3)C(=O)C=C)c3nc(Oc4ccccn4)ccc3c2c1	6.85
BDBM50514391	CC(C)c1ccccc1-n1c2nc(c(Cl)cc2c(nc1=O)N1CCN(C[C@@H]1C)C(=O)C=C)-c1ccccc1O	6.84
CHEMBL4450041	C=CC(=O)N1CCN(c2nc(=O)n(-c3ccccc3C(C)C)c3nc(-c4ccccc4O)c(Cl)cc23)[C@@H](C)C1	6.84
BDBM544072	CNC(=O)c1ccccc1-c1nc2n(-c3c(C)ccnc3C(C)C)c(=O)nc(N3C[C@@H](C)N(C[C@@H]3C)C(=O)C=C)c2cc1Cl	6.83
BDBM515907	CCc1c(C(=O)N2CCc3c(C2)cccc3OC)c2cc(Cl)cc(C)c2n1CC(=O)N(C)C1CN(C1)C(=O)C=C	6.82
BDBM515894	CCc1c(C(=O)N2CCN(C[C@H]2C)c2ccc(cn2)C#N)c2cc(Cl)cc(C)c2n1CC(=O)NC1CN(C1)C(=O)C=C	6.82
BDBM515990	Cc1cc(Br)c2CCN(Cc2c1)C(=O)c1cn(CC(=O)NC2CN(C2)C(=O)C=C)c2c(C)cc(Br)cc12	6.82
BDBM50527048	CN1C(=O)[C@H]2CN(CCN2c2c1cnc1cc(c(Cl)cc21)-c1c(O)cccc1F)C(=O)C=C	6.82
CHEMBL4551121	C=CC(=O)N1CCN2c3c(cnc4cc(-c5c(O)cccc5F)c(Cl)cc34)N(C)C(=O)[C@H]2C1	6.82
CHEMBL4591653	C=CC(=O)N1CC(NC(=O)Cn2c(C)c(C(=O)N3CCc4c(cccc4OC)C3)c3cc(Cl)cc(C)c32)C1	6.82
BDBM544285	CC(C)c1nccc(C)c1-n1c2nc(c(Cl)cc2c(nc1=O)N1C[C@@H](C)N(C[C@@H]1C)C(=O)C=C)-c1ccccc1NC(=O)C(F)F	6.82
BDBM535158	Nc1ccc(c(n1)-c1cc2ncnc(N3CCN(CC3)C(=O)C=C)c2cc1N1CCC1)C(F)(F)F	6.82
CHEMBL3577124	CNc1ncc2cc(-c3cc(NC(=O)NCCC(C)(C)C)c(F)cc3C)c(C)nc2n1	6.82

BDBM515903	COc1cccc2[C@@H](C)N([C@H](C)Cc12)C(=O)c1c(C)n(CC(=O)NC2CN(C2)C(=O)C=C)c2c(C)cc(Br)cc12	6.8
BDBM544164	CC(C)c1ncnc(C(C)C)c1-n1c2nc(c(F)cc2c(nc1=O)N1C[C@@H](C)N(C[C@@H]1C)C(=O)C=C)-c1ccccc1F	6.8
BDBM515900	C[C@H]1N(CCc2ccccc12)C(=O)c1c(C)n(CC(=O)NC2CN(C2)C(=O)C=C)c2c(C)cc(Br)cc12	6.8
CHEMBL3577117	CNc1cc2nc(C)c(-c3cc(NC(=O)NC[C@@H](O)C(C)(C)C)c(F)cc3C)cc2cn1	6.8
BDBM516068	CN(CC(=O)Nc1ccc(Cl)c(c1)C(F)(F)F)C(=O)c1cn(CC(=O)NC2CN(C2)C(=O)C=C)c2ccc(Br)cc12	6.8
CHEMBL4528930	C=CC(=O)N1CCN(c2nc(=O)n(-c3ccnn3C(C)C)c3nc(-c4c(O)cccc4F)c(Cl)cc23)[C@@H](C)C1	6.79
BDBM50514380	CC(C)n1nccc1-n1c2nc(c(Cl)cc2c(nc1=O)N1CCN(C[C@@H]1C)C(=O)C=C)-c1c(O)cccc1F	6.79
BDBM548351	CC(C)C[C@@H](Cn1cccn1)Nc1nc(nc2CC(C)(C)CCc12)N1CCC2(CN(C2)C(=O)C=C)C1	6.79
BDBM544097	CC(C)c1nccc(C)c1-n1c2nc(c(Cl)cc2c(nc1=O)N1C[C@@H](C)N(C[C@@H]1C)C(=O)C=C)-c1cccc(F)c1	6.79
BDBM544286	CC(C)c1nccc(C)c1-n1c2nc(c(Cl)cc2c(nc1=O)N1C[C@@H](C)N(C[C@@H]1C)C(=O)C=C)-c1cccc(F)c1NC(C)=O	6.78
BDBM544297	CC(C)c1nccc(C)c1-n1c2nc(c(Cl)cc2c(nc1=O)N1CCN(C(CC#N)C1)C(=O)C=C)-c1c(O)cccc1F	6.78
BDBM544293	CC(C)c1nccc(C)c1-n1c2nc(c(Cl)cc2c(nc1=O)N1CCN(C(CC#N)C1)C(=O)C=C)-c1c(N)cccc1F	6.78
BDBM516087	CCc1ccccc1NC(=O)CCNC(=O)c1cn(CC(=O)NC2CN(C2)C(=O)C=C)c2ccc(Br)cc12	6.77
BDBM516032	Cc1ccc2CCN(Cc2c1)C(=O)c1cn(CC(=O)NC2CN(C2)C(=O)C=C)c2ccc(Br)cc12	6.77
CHEMBL4467039	C=CC(=O)N1CCN(c2nc(N3CC(N(C)C)C3)nc3c2C[C@@H](C)[C@H](c2c(Cl)ccc4[nH]ncc24)C3)CC1	6.77
BDBM50517241	C[C@@H]1Cc2c(C[C@H]1c1c(Cl)ccc3[nH]ncc13)nc(nc2N1CCN(CC1)C(=O)C=C)N1CC(C1)N(C)C	6.77
BDBM50517184	COc1cccc2CN(CCc12)C(=O)c1cn(CC(=O)NC2CN(C2)C(=O)C=C)c2ccc(Br)cc12	6.77
BDBM515922	COc1nccc2CN(CCc12)C(=O)c1c(C)n(CC(=O)NC2CN(C2)C(=O)C=C)c2ccc(Br)cc12	6.77
BDBM515944	Cc1c(C(=O)N2CCN(CC2)c2ccc(Br)cn2)c2cc(Br)ccc2n1CC(=O)NC1CN(C1)C(=O)C=C	6.77
BDBM515947	Cc1cccc(C)c1OCCNC(=O)c1cn(CC(=O)NC2CN(C2)C(=O)C=C)c2c(Cl)cc(Cl)cc12	6.77
BDBM515984	Cc1cccc2CN(CCc12)C(=O)c1cn(CC(=O)NC2CN(C2)C(=O)C=C)c2c(Cl)cc(Cl)cc12	6.77
BDBM515996	Cc1cccc2CN(CCc12)C(=O)c1cn(CC(=O)NC2CN(C2)C(=O)C=C)c2ccc(Br)cc12	6.77
BDBM544099	CC(C)c1nccc(C)c1-n1c2nc(c(Cl)cc2c(nc1=O)N1C[C@@H](C)N(C[C@@H]1C)C(=O)C=C)-c1cccc(Cl)c1	6.77
CHEMBL4457550	C=CC(=O)N1CC(NC(=O)Cn2cc(C(=O)N3CCc4c(cccc4OC)C3)c3cc(Br)cc(C)c32)C1	6.76
BDBM544051	CC(C)c1nccc(C)c1-n1c2nc(C3=CCCCC3)c(Cl)cc2c(nc1=O)N1C[C@@H](C)N(C[C@@H]1C)C(=O)C=C	6.76
BDBM544237	CC(C)c1nccc(N(C)C)c1-n1c2nc(c(Cl)cc2c(nc1=O)N1C[C@@H](C)N(C[C@@H]1C)C(=O)C=C)-c1c(F)cccc1NC(=O)C1CC1	6.75
BDBM516017	COc1cccc2CN(CCc12)C(=O)c1cn(CC(=O)N(C)C2CN(C2)C(=O)C=C)c2ccc(Br)cc12	6.74
BDBM516014	Brc1ccc2CN(CCc2c1)C(=O)c1cn(CC(=O)NC2CN(C2)C(=O)C=C)c2ccc(Br)cc12	6.74
BDBM516002	Cc1cc(Br)c2CCN(Cc2c1)C(=O)c1cn(CC(=O)NC2CN(C2)C(=O)C=C)c2c(Cl)cc(Cl)cc12	6.74
BDBM544177	CC(C)c1cc(cc(C)c1-n1c2nc(c(Cl)cc2c(nc1=O)N1C[C@@H](C)N(C[C@@H]1C)C(=O)C=C)-c1ccccc1F)P(C)(C)=O	6.74
BDBM544131	CC(C)c1nccc(C)c1-n1c2nc(c(F)cc2c(nc1=O)N1C[C@@H](C)N(C[C@@H]1C)C(=O)C=C)-c1c(F)cccc1Cl	6.74
BDBM535148	Cc1cc(N)nc(c1C)-c1cc2ncnc(N3CCN(CC3)C(=O)C=C)c2cc1Cl	6.74
BDBM515938	COc1nccc2CN(CCc12)C(=O)c1c(C)n(CC(=O)NC2CN(C2)C(=O)C=C)c2ccc(Cl)cc12	6.74
BDBM515961	Cc1c(C(=O)N2CCc3c(C)cc(Br)cc3C2)c2cc(Br)ccc2n1CC(=O)NC1CN(C1)C(=O)C=C	6.74
BDBM544430	CC(C)c1nccc(C)c1-n1c2nc(c(Cl)cc2c(nc1=O)N1C[C@@H](C)N(C[C@@H]1C)C(=O)C=C)-c1cc(ccc1F)S(C)(=O)=O	6.74
BDBM548331	CNC(=O)C[C@H](CC(C)C)Nc1nc(nc2cc(ccc12)C#N)N1CCC2(CN(C2)C(=O)C=C)C1	6.74
BDBM515963	CN(C1CN(C1)C(=O)C=C)C(=O)Cn1c(C)c(C(=O)N2CCN(CC2)c2ccc(C)cn2)c2cc(Cl)cc(C)c12	6.74
BDBM544325	CCCS(=O)(=O)c1nc(C(C)C)c(c(n1)C(C)C)-n1c2nc(c(Cl)cc2c(nc1=O)N1C[C@@H](C)N(C[C@@H]1C)C(=O)C=C)-c1ccccc1F	6.72
BDBM515932	C[C@@H]1CN(C[C@@H](C)N1C(=O)c1c(C)n(CC(=O)NC2CN(C2)C(=O)C=C)c2ccc(Br)cc12)c1ccc(cn1)C#N	6.72
BDBM50517188	Brc1ccc2n(CC(=O)NC3CN(C3)C(=O)C=C)cc(C(=O)N3CCc4ccccc4C3)c2c1	6.72
BDBM544279	CC(C)c1nccc(C)c1-n1c2nc(c(Cl)cc2c(nc1=O)N1C[C@@H](C)N(C[C@@H]1C)C(=O)C=C)-c1ccccc1NC(=O)C(F)(F)F	6.72

BDBM544292	CC(C)c1ccccc1-c1nc2n(-c3c(CN4CCCC4)ccnc3C(C)C)c(=O)nc(N3C[C@@H](C)N(C[C@@H]3C)C(=O)C=C)c2cc1Cl	6.71
BDBM548333	CNC(=O)C[C@H](CC(C)C)Nc1nc(nc2CC(C)CCc12)N1CC(F)C2(CN(C2)C(=O)C=C)C1	6.71
BDBM544228	CC(C)c1nc(CN(C)C)cc(C)c1-n1c2nc(c(Cl)cc2c(nc1=O)N1C[C@@H](C)N(C[C@@H]1C)C(=O)C=C)-c1ccccc1F	6.71
BDBM544165	CC(C)c1ncnc(C(C)C)c1-n1c2nc(c(F)cc2c(nc1=O)N1[C@@H](C)CN(C[C@H]1C)C(=O)C=C)-c1ccccc1F	6.71
BDBM50514382	C[C@H]1CN(CCN1c1nc(=O)n(-c2c(C)cccc2C)c2nc(c(Cl)cc12)-c1c(O)cccc1F)C(=O)C=C	6.7
CHEMBL4458945	C=CC(=O)N1CCN(c2nc(=O)n(-c3c(C)cccc3C)c3nc(-c4c(O)cccc4F)c(Cl)cc23)[C@@H](C)C1	6.7
BDBM535195	Nc1ccc(OC(F)(F)F)c(n1)-c1cc2ncnc(N3CCN(CC3)C(=O)C=C)c2cc1Cl	6.7
BDBM535169	Cc1cc(N)nc(-c2cc3ncnc(N4CCN(C(C4)C#N)C(=O)C4=CCC4)c3cc2Cl)c1C(F)(F)F	6.7
BDBM515946	Cc1c(-c2ccccc2C2CC2)c2cc(Cl)ccc2n1CC(=O)NC1CN(C1)C(=O)C=C	6.7
BDBM515964	CCc1ccccc1-c1c(C)n(CC(=O)NC2CN(C2)C(=O)C=C)c2ccc(Cl)cc12	6.7
BDBM544058	CC(C)c1nccc(C)c1-n1c2nc(c(Cl)cc2c(nc1=O)N1C[C@@H](C)N(C[C@@H]1C)C(=O)C=C)-c1cccnc1N	6.7
BDBM515919	C[C@H]1Cc2cc(F)ccc2[C@@H](C)N1C(=O)c1c(C)n(CC(=O)NC2CN(C2)C(=O)C=C)c2c(C)cc(Br)cc12	6.7
BDBM544342	CC(C)c1nc(nc(C(C)C)c1-n1c2nc(c(Cl)cc2c(nc1=O)N1C[C@@H](C)N(C[C@@H]1C)C(=O)C=C)-c1ccccc1F)-c1ccoc1	6.69
CHEMBL4456368	C=CC(=O)N1CCN2c3c(cnc4cc(-c5c(O)cccc5F)c(Cl)cc34)OC[C@H]2C1	6.68
BDBM50527061	Oc1cccc(F)c1-c1cc2ncc3OC[C@H]4CN(CCN4c3c2cc1Cl)C(=O)C=C	6.68
BDBM50517242	C[C@@H]1Cc2c(C[C@H]1c1c(C)ccc3[nH]ncc13)nc(OCCCN(C)C)nc2N1CCN(CC1)C(=O)C=C	6.67
BDBM548306	CNC(=O)C[C@H](CC(C)C)Nc1nc(nc2CC(C)CCc12)N1CCC2(CN(C2)C(=O)C=C)C1	6.67
CHEMBL4466335	C=CC(=O)N1CCN(c2nc(OCCCN(C)C)nc3c2C[C@@H](C)[C@H](c2c(C)ccc4[nH]ncc24)C3)CC1	6.67
CHEMBL4454645	C=CC(=O)N1CCN(c2nc(=O)n(-c3ccccc3C(C)C)c3cc(-c4ccccc4Cl)c(Cl)cc23)[C@@H](C)C1	6.66
BDBM50514403	CC(C)c1ccccc1-n1c2cc(c(Cl)cc2c(nc1=O)N1CCN(C[C@@H]1C)C(=O)C=C)-c1ccccc1Cl	6.66
BDBM515914	CC(C)c1ccccc1-c1c(C)n(CC(=O)NC2CN(C2)C(=O)C=C)c2ccc(Cl)cc12	6.66
BDBM516088	Clc1ccc(CCNC(=O)c2cn(CC(=O)NC3CN(C3)C(=O)C=C)c3c(Cl)cc(Cl)cc23)c(Cl)c1	6.66
BDBM515918	C[C@@H]1CN(CCN1C(=O)c1c(C)n(CC(=O)N(C)C2CN(C2)C(=O)C=C)c2ccc(Br)cc12)c1ccc(cn1)C#N	6.66
BDBM515925	COc1nccc2CN(CCc12)C(=O)c1cn(CC(=O)NC2CN(C2)C(=O)C=C)c2ccc(Cl)c(Cl)c12	6.66
BDBM515948	CN(C1CN(C1)C(=O)C=C)C(=O)Cn1c(C)c(C(=O)N2CCN(CC2)c2ccc(cn2)[N+]([O-])=O)c2cc(Br)ccc12	6.66
BDBM544123	CC(C)c1nccc(C)c1-n1c2nc(c(F)cc2c(nc1=O)N1C[C@@H](C)N(C[C@@H]1C)C(=O)C=C)-c1cc(C)ccc1F	6.65
BDBM544389	CC(C)c1nc(C)c(c(n1)C(C)C)-n1c2nc(c(Cl)cc2c(nc1=O)N1C[C@@H](C)N(C[C@@H]1C)C(=O)C=C)-c1ccccc1F	6.65
CHEMBL4873683	Cc1ccnc(C(C)C)c1-n1c(=O)nc(N(C)C2CN(C(=O)C3CO3)C2)c2cc(Cl)c(-c3ccccc3F)nc21	6.64
BDBM535167	Cc1cc(N)nc(-c2cc3ncnc(c3cc2Cl)C2(CCN(CC2)C(=O)C=C)C#N)c1C(F)(F)F	6.64
BDBM50573430	CC(C)c1nccc(C)c1-n1c2nc(c(Cl)cc2c(nc1=O)N(C)C1CN(C1)C(=O)C1CO1)-c1ccccc1F	6.64
BDBM515965	Brc1ccc2n(CC(=O)NC3CN(C3)C(=O)C=C)c3nc(Oc4cccnc4)ccc3c2c1	6.64
BDBM516071	Clc1ccc(CCNC(=O)c2cn(CC(=O)NC3CN(C3)C(=O)C=C)c3ccc(Br)cc23)c(Cl)c1	6.64
BDBM515993	Cc1c(C(=O)N2CCN(CC2)c2ccc(cn2)C#N)c2cc(Br)ccc2n1CC(=O)NC1CN(C1)C(=O)C=C	6.64
BDBM544244	CC(C)c1nc(N)cc(C)c1-n1c2nc(c(F)cc2c(nc1=O)N1C[C@H](C)N([C@H](C)C1)C(=O)C=C)-c1c(N)cccc1F	6.64
BDBM535134	Nc1cc2ccccc2c(n1)-c1c(Cl)cc2c(ncnc2c1F)N1CCN(CC1)C(=O)C=C	6.62
BDBM535161	CC(F)(F)c1cc2c(ncnc2cc1-c1nc(N)ccc1C(F)(F)F)N1CCN(CC1)C(=O)C=C	6.62
BDBM515977	Cc1c(-c2ccccc2-c2ccccc2)c2cc(Cl)ccc2n1CC(=O)NC1CN(C1)C(=O)C=C	6.62
BDBM515937	CN(C1CN(C1)C(=O)C=C)C(=O)Cn1c(C)c(C(=O)N2CCN(CC2)c2ccc(cn2)C#N)c2cc(Br)ccc12	6.62
BDBM544301	CC(C)c1nccc(C)c1-n1c2nc(c(Cl)cc2c(nc1=O)N1CCN(C(CC#N)C1)C(=O)C=C)-c1ccccc1F	6.62
BDBM544093	CC(C)c1nccc(C)c1-n1c2nc(c(Cl)cc2c(nc1=O)N1C[C@@H](C)N(C[C@@H]1C)C(=O)C=C)-c1cccc(c1)C#C	6.61
BDBM544085	CC(C)c1nccc(C)c1-n1c2nc(c(Cl)cc2c(nc1=O)N1C[C@@H](C)N(C[C@@H]1C)C(=O)C=C)-c1c(F)cccc1C#N	6.61

CHEMBL4645768	C=CC(=O)N1CCN(c2nc(OC[C@@H]3CCCN3C)nc3c2CCN(c2c(C)c(C)cc4[nH]ncc24)C3)CC1	6.6
BDBM50539747	CN1CCC[C@H]1COc1nc2CN(CCc2c(n1)N1CCN(CC1)C(=O)C=C)c1c(C)c(C)cc2[nH]ncc12	6.6
BDBM515972	Cc1cc(Br)cc2c(cn(CC(=O)NC3CN(C3)C(=O)C=C)c12)-c1ccccc1-c1ccccc1	6.6
BDBM516034	COC(=O)c1ccc2CN(CCc2c1)C(=O)c1cn(CC(=O)NC2CN(C2)C(=O)C=C)c2ccc(Br)cc12	6.59
BDBM516063	Cc1cc(Br)cc2c(I)cn(CC(=O)NC3CN(C3)C(=O)C=C)c12	6.59
BDBM516072	Cc1cc(Br)cc2c(cn(CC(=O)NC3CN(C3)C(=O)C=C)c12)C(=O)NCCc1ccc(Cl)cc1Cl	6.59
BDBM516077	CCc1ccccc1NC(=O)CCNC(=O)c1cn(CC(=O)NC2CN(C2)C(=O)C=C)c2ccc(Br)cc12	6.59
BDBM544127	CC(C)c1nccc(C)c1-n1c2nc(c(F)cc2c(nc1=O)N1C[C@@H](C)N(C[C@@H]1C)C(=O)C=C)-c1ccccc1C	6.59
BDBM548335	CNC(=O)C[C@H](CC(C)C)Nc1nc(nc2cc(Cl)ccc12)N1CCC2(CN(C2)C(=O)C=C)C1	6.58
BDBM516155	Cc1cc(Br)c2CCN(Cc2c1)C(=O)c1cn(CC(=O)NC2CN(C2)C(=O)C=C)c2ccc(cc12)[N+]([O-])=O	6.57
BDBM544290	CCN(CC)Cc1ccnc(C(C)C)c1-n1c2nc(c(Cl)cc2c(nc1=O)N1C[C@@H](C)N(C[C@@H]1C)C(=O)C=C)-c1ccccc1C(C)C	6.57
CHEMBL3577118	CNc1cc2nc(C)c(-c3cc(NC(=O)NCCC(C)(C)C)ccc3C)cc2cn1	6.57
BDBM544081	CC(C)c1nccc(C)c1-n1c2nc(c(Cl)cc2c(nc1=O)N1C[C@@H](C)N(C[C@@H]1C)C(=O)C=C)-c1ccccc1[N+]([O-])=O	6.56
BDBM515952	COc1cccc2CN(CCc12)C(=O)c1c(C)n(C2CCN(C3CN(C3)C(=O)C=C)C2=O)c2ccc(Br)cc12	6.55
BDBM516076	[O-][N+](=O)c1ccc(cc1)N1CCN(CC1)C(=O)c1cn(CC(=O)NC2CN(C2)C(=O)C=C)c2ccc(Br)cc12	6.55
BDBM544291	CCN(Cc1ccnc(C(C)C)c1-n1c2nc(c(Cl)cc2c(nc1=O)N1C[C@@H](C)N(C[C@@H]1C)C(=O)C=C)-c1ccccc1C(C)C)C(C)C	6.55
BDBM515999	COc1nccc2CN(CCc12)C(=O)c1cn(CC(=O)NC2CN(C2)C(=O)C=C)c2ccc(Br)cc12	6.55
BDBM516030	Cc1cc(Br)c2CCN(Cc2c1)C(=O)c1cn(CC(=O)NC2CN(C2)C(=O)C=C)c2ccc(Br)cc12	6.55
BDBM535174	Nc1ccc(c(n1)-c1cc2ncnc(N3CCN(C(C3)C(F)(F)F)C(=O)C=C)c2cc1Cl)C(F)(F)F	6.54
BDBM535213	Nc1ccc(CC(F)(F)F)c(n1)-c1cc2ncnc(N3CCN(CC3)C(=O)C=C)c2cc1Cl	6.54
BDBM515945	Cc1c(C(=O)N2CCc3ccccc3C2(C)C)c2cc(Br)cc(C)c2n1CC(=O)NC1CN(C1)C(=O)C=C	6.54
BDBM544329	CC(C)c1nc(CN2CCC2)nc(C(C)C)c1-n1c2nc(c(Cl)cc2c(nc1=O)N1C[C@@H](C)N(C[C@@H]1C)C(=O)C=C)-c1ccccc1F	6.53
BDBM515957	CN(C1CN(C1)C(=O)C=C)C(=O)Cn1c(C)c(C(=O)N2CCN(CC2)c2ccc(C)cn2)c2cc(Br)ccc12	6.52
BDBM515927	COc1cccc2CN(CCc12)C(=O)c1c(C)n(CC(=O)NC2CN(C2)C(=O)C=C)c2ccc(cc12)[N+]([O-])=O	6.52
BDBM544119	CC(C)c1nccc(C)c1-n1c2nc(C3CC3(C)C)c(Cl)cc2c(nc1=O)N1C[C@@H](C)N(C[C@@H]1C)C(=O)C=C	6.52
CHEMBL4520093	C=CC(=O)N1CC(NC(=O)Cn2c(C)c(C(=O)N3CCc4c(cccc4OC)C3)c3cc(Br)ccc32)C1	6.52
BDBM544145	CC(C)c1nccc(C)c1-n1c2nc(C3CCC3)c(Cl)cc2c(nc1=O)N1C[C@@H](C)N(C[C@@H]1C)C(=O)C=C	6.52
BDBM50517178	Fc1ccc2CN(CCc2c1)C(=O)c1cn(CC(=O)NC2CN(C2)C(=O)C=C)c2ccc(Br)cc12	6.51
BDBM50517186	Clc1cccc2CN(CCc12)C(=O)c1cn(CC(=O)NC2CN(C2)C(=O)C=C)c2ccc(Br)cc12	6.51
BDBM516006	Clc1ccc2CN(CCc2c1Cl)C(=O)c1cn(CC(=O)NC2CN(C2)C(=O)C=C)c2ccc(Br)cc12	6.51
BDBM544317	C[C@@H]1CN([C@@H](C)CN1C(=O)C=C)c1nc(=N)n(CC(C)(C)C)c2nc(c(Cl)cc12)-c1ccccc1F	6.5
BDBM544256	CC(C)c1nccc(C)c1-n1c2nc(c(Cl)cc2c(nc1=O)N1C[C@@H](C)N(C[C@@H]1C)C(=O)C=C)-c1cc(OC(F)(F)F)ccc1F	6.5
BDBM516144	Fc1ccc(Oc2ccc3c4cc(Br)ccc4n(CC(=O)NC4CN(C4)C(=O)C=C)c3n2)cc1	6.49
BDBM544121	CC(C)c1nccc(C)c1-n1c2nc(c(F)cc2c(nc1=O)N1C[C@@H](C)N(C[C@@H]1C)C(=O)C=C)-c1ccccc1CO	6.49
BDBM544155	CC(C)c1ncnc(C(C)C)c1-n1c2nc(c(Cl)cc2c(nc1=O)N1C[C@@H](C)N(C[C@@H]1C)C(=O)C=C)-c1ccc(cc1F)C#N	6.49
BDBM516009	[O-][N+](=O)c1ccc(nc1)N1CCN(CC1)C(=O)c1cn(CC(=O)NC2CN(C2)C(=O)C=C)c2ccc(Br)cc12	6.48
BDBM544227	CC(C)c1nccc(C)c1-n1c2nc(c(F)cc2c(nc1=O)N1C[C@@H](C)N(C[C@@H]1C)C(=O)C#CCN(C)C)-c1ccccc1F	6.48
BDBM515926	C[C@@H]1CN(CCN1C(=O)c1c(C)n(CC(=O)NC2CN(C2)C(=O)C=C)c2ccc(Br)cc12)c1ccc(cn1)C#N	6.48
BDBM515949	CC1Cc2ccccc2CN1C(=O)c1c(C)n(CC(=O)NC2CN(C2)C(=O)C=C)c2c(C)cc(Br)cc12	6.48
BDBM548321	CNC(=O)C[C@H](CC(C)C)Nc1nc(nc2ccccc12)N1CCC2(CN(C2)C(=O)C=C)C(F)(F)C1	6.48
BDBM548330	CNC(=O)C[C@H](CC(C)C)Nc1nc(nc2cc(C)ccc12)N1CCC2(CN(C2)C(=O)C=C)C1	6.47

BDBM515916	COc1cccc2CN(CCc12)C(=O)c1c(C)n(CC(=O)NC2CN(C2)C(=O)C=C)c2c(C)cc(C)cc12	6.47
BDBM535181	Cc1cc(N)nc(-c2cc3nc(nc(N4CCN(CC4)C(=O)C=C)c3cc2Cl)C(F)(F)F)c1C(F)(F)F	6.46
BDBM535199	Nc1nc(ccc1CC(F)(F)F)-c1cc2ncnc(N3CCN(CC3)C(=O)C=C)c2cc1Cl	6.46
BDBM544148	CC(C)c1nccc(C)c1-n1c2nc(c(Cl)cc2c(nc1=O)N1C[C@@H](C)N(C[C@@H]1C)C(=O)C=C)-c1ncccc1C	6.46
BDBM544318	C\N=c1/nc(N2C[C@@H](C)N(C[C@@H]2C)C(=O)C=C)c2cc(Cl)c(nc2n1CC(C)(C)C)-c1ccccc1F	6.46
BDBM516021	COC(=O)c1c(I)c2cc(Br)ccc2n1CC(=O)NC1CN(C1)C(=O)C=C	6.46
BDBM50539746	CN1CCC[C@H]1COc1nc2CN(CCc2c(n1)N1CCN(CC1)C(=O)C=C)c1c(ccc2[nH]ncc12)C(F)(F)F	6.45
CHEMBL4642659	C=CC(=O)N1CCN(c2nc(OC[C@@H]3CCCN3C)nc3c2CCN(c2c(C(F)(F)F)ccc4[nH]ncc24)C3)CC1	6.45
BDBM544277	CC(C)c1nccc(C)c1-n1c2nc(c(Cl)cc2c(nc1=O)N1C[C@@H](C)N(C[C@@H]1C)C(=O)C=C)-c1c(F)cccc1N(C)C(C)=O	6.45
BDBM543967	CC(C)c1nccc(N(C)C)c1-n1c2nc(c(Cl)cc2c(nc1=O)N1C[C@@H](C)N(C[C@@H]1C)C(=O)C=C)-c1ccccc1S(C)(=O)=O	6.45
BDBM535184	Cc1c(cc(N)nc1-c1cc2ncnc(N3CCN(CC3)C(=O)C=C)c2cc1Cl)C(F)(F)F	6.44
BDBM515956	Cc1c(C(=O)N2CCN(CC2)c2ccc(cn2)[N+]([O-])=O)c2cc(Br)ccc2n1CC(=O)NC1CN(C1)C(=O)C=C	6.44
BDBM516001	Cc1cc(Cl)cc2c(Br)cn(CC(=O)NC3CN(C3)C(=O)C=C)c12	6.43
BDBM516244	CC1CCC(=CC1)c1ccncc1-c1c(C)n(CC(=O)NC2CN(C2)C(=O)C=C)c2c(C)cc(Cl)cc12	6.43
CHEMBL4573277	C=CC(=O)N1CC(NC(=O)Cn2c(C)c(C(=O)N3CCc4c(cccc4OC)C3)c3cc(Cl)ccc32)C1	6.43
BDBM544323	COCCOc1nc(C(C)C)c(c(n1)C(C)C)-n1c2nc(c(Cl)cc2c(nc1=O)N1C[C@@H](C)N(C[C@@H]1C)C(=O)C=C)-c1ccccc1F	6.43
BDBM548339	CNC(=O)C[C@H](CC(C)C)Nc1nc(nc2cc(C)cnc12)N1CCC2(CN(C2)C(=O)C=C)C1	6.43
BDBM544040	CC(C)c1cc(CN(C)C)cc(C)c1-n1c2nc(c(F)cc2c(nc1=O)N1C[C@@H](C)N(C[C@@H]1C)C(=O)C=C)-c1ccccc1F	6.43
BDBM548349	CC(C)C[C@@H](Cc1noc(C)n1)Nc1nc(nc2CC(C)(C)CCc12)N1CCC2(CN(C2)C(=O)C=C)C1	6.42
BDBM515915	Cc1c(-c2ccccc2N2CCCC2)c2cc(Cl)ccc2n1CC(=O)NC1CN(C1)C(=O)C=C	6.42
BDBM544320	CC(C)c1ncnc(C(C)C)c1-n1c2nc(c(F)cc2c(nc1=O)N1C[C@@H](C)N(C[C@@H]1C)C(=O)C=C)-c1ccc(F)cc1F	6.42
BDBM544021	CC(C)c1nccc(C)c1-n1c2nc(c(F)cc2c(nc1=O)N1CCN(CC1)C(=O)C=C)-c1c(O)cccc1F	6.42
BDBM516103	Brc1ccc2n(CC(=O)NC3CN(C3)C(=O)C=C)c3ccc(Br)cc3c2c1	6.41
CHEMBL4560503	C=CC(=O)N1CCN2c3c(cnc4cc(-c5c(O)cccc5F)c(Cl)cc34)NC[C@H]2C1	6.41
BDBM544132	CC(C)c1nccc(C)c1-n1c2nc(c(F)cc2c(nc1=O)N1C[C@@H](C)N(C[C@@H]1C)C(=O)C=C)-c1ccccc1C(F)F	6.41
BDBM516029	CC(C)c1ccccc1-c1cn(CC(=O)NC2CN(C2)C(=O)C=C)c2c(C)cc(Br)cc12	6.41
BDBM544136	CC(C)c1nccc(C)c1-n1c2nc(c(F)cc2c(nc1=O)N1C[C@@H](C)N(C[C@@H]1C)C(=O)C=C)-c1ccccc1C(N)=O	6.41
BDBM50527047	Oc1cccc(F)c1-c1cc2ncc3NC[C@H]4CN(CCN4c3c2cc1Cl)C(=O)C=C	6.41
BDBM548314	CNC(=O)C[C@H](CC(C)C)Nc1nc(nc2scnc12)N1CCC2(CN(C2)C(=O)C=C)C1	6.4
BDBM544239	CC(C)c1nc(N)cc(C)c1-n1c2nc(c(F)cc2c(nc1=O)N1C[C@H](C)N([C@H](C)C1)C(=O)C=C)-c1c(O)cccc1F	6.4
BDBM544065	CC(C)c1nccc(C)c1-n1c2nc(c(Cl)cc2c(nc1=O)N1C[C@@H](C)N(C[C@@H]1C)C(=O)C=C)-c1cccc2CC(=O)Nc12	6.39
BDBM516157	COc1cccc2CN(CCc12)C(=O)c1cn(CC(=O)NC2CN(C2)C(=O)C=C)c2ccc(cc12)[N+]([O-])=O	6.39
BDBM548363	CNC(=O)C[C@H](CC(C)C)Nc1nc(nc2cc(C)ccc12)N1CCC2(CN(C2)C(=O)C(=C)C(F)(F)F)C1	6.39
BDBM516031	Cc1c(-c2ccccc2C)c2cc(Cl)ccc2n1CC(=O)NC1CN(C1)C(=O)C=C	6.38
BDBM515902	C[C@@H]1N(CCc2ccccc12)C(=O)c1c(C)n(CC(=O)NC2CN(C2)C(=O)C=C)c2c(C)cc(Br)cc12	6.38
BDBM516044	Brc1ccc2n(CC(=O)NC3CN(C3)C(=O)C=C)cc(C(=O)N3CCc4c(C3)cccc4C#N)c2c1	6.38
BDBM516027	Brc1cccc2n(CC(=O)NC3CN(C3)C(=O)C=C)c3ccccc3c12	6.37
BDBM548336	CNC(=O)C[C@H](CC(C)C)Nc1nc(nc2CCCOc12)N1CCC2(CN(C2)C(=O)C=C)C1	6.37
BDBM548309	CNC(=O)C[C@H](CC(C)C)Nc1nc(cc2ccccc12)N1CCC2(CN(C2)C(=O)C=C)C1	6.37
BDBM515985	Clc1ccnc2n(CC(=O)NC3CN(C3)C(=O)C=C)c3ccc(Br)cc3c12	6.37
BDBM544249	CC(C)c1ccnc(C(C)C)c1-n1c2nc(c(F)cc2c(nc1=O)N1C[C@@H](C)N(C[C@@H]1C)C(=O)C=C)-c1ccccc1C	6.36

BDBM516066	Cc1ccccc1OCCNC(=O)c1cn(CC(=O)NC2CN(C2)C(=O)C=C)c2ccc(Br)cc12	6.36
BDBM515953	CN(C1CN(C1)C(=O)C=C)C(=O)Cn1cc(I)c2cc(Br)ccc12	6.36
CHEMBL4750445	Cn1cnc(Cn2ccc3ccc(CNCc4[nH]c5ccccc5c4[C@H]4NC(=O)c5ccc(O)cc54)cc32)c1	6.35
CHEMBL4438343	C=CC(=O)N1CCN(c2nc(=O)n(-c3ccccc3C(C)C)c3cc(-c4ccccc4C(F)(F)F)c(Cl)cc23)[C@@H](C)C1	6.35
BDBM50549225	Cn1cnc(Cn2ccc3ccc(CNCc4[nH]c5ccccc5c4[C@H]4NC(=O)c5ccc(O)cc45)cc23)c1	6.35
BDBM50514405	CC(C)c1ccccc1-n1c2cc(c(Cl)cc2c(nc1=O)N1CCN(C[C@@H]1C)C(=O)C=C)-c1ccccc1C(F)(F)F	6.35
BDBM548338	CNC(=O)C[C@H](CC(C)C)Nc1nc(nc2CCC(C)Cc12)N1CCC2(CN(C2)C(=O)C=C)C1	6.35
BDBM515973	Cc1ccc2n(CC(=O)NC3CN(C3)C(=O)C=C)cc(I)c2c1	6.35
BDBM515917	COc1nccc2CN(CCc12)C(=O)c1cn(CC(=O)N(C)C2CN(C2)C(=O)C=C)c2ccc(Cl)c(Cl)c12	6.35
BDBM535150	Cc1ccc(N)nc1-c1cc2ncnc(N3CCN(CC3)C(=O)C=C)c2cc1Cl	6.35
BDBM544139	CC(C)c1nccc(C)c1-n1c2nc(c(F)cc2c(nc1=O)N1C[C@@H](C)N(C[C@@H]1C)C(=O)C=C)-c1ccccc1N	6.34
BDBM544232	CC(C)c1nccc(N(C)C)c1-n1c2nc(c(Cl)cc2c(nc1=O)N1C[C@@H](C)N(C[C@@H]1C)C(=O)C=C)-c1c(F)cccc1NC(=O)C(F)(F)F	6.34
BDBM544128	CC(C)c1nccc(C)c1-n1c2nc(c(F)cc2c(nc1=O)N1C[C@@H](C)N(C[C@@H]1C)C(=O)C=C)-c1c(C)cccc1C	6.33
BDBM516062	CCC(CNC(=O)c1cn(CC(=O)NC2CN(C2)C(=O)C=C)c2ccc(Br)cc12)Oc1cccc(Cl)c1	6.33
BDBM544125	COc1ccc(F)c(c1)-c1nc2n(-c3c(C)ccnc3C(C)C)c(=O)nc(N3C[C@@H](C)N(C[C@@H]3C)C(=O)C=C)c2cc1F	6.33
BDBM516058	Brc1ccc2n(CC(=O)NC3CN(C3)C(=O)C=C)cc(C(=O)N3CC4(CCN4c4ccccn4)C3)c2c1	6.33
BDBM548312	CNC(=O)C[C@H](CC(C)C)Nc1nc(nc2CN(Cc12)C(C)=O)N1CCC2(CN(C2)C(=O)C=C)C1	6.33
BDBM516000	Clc1ccc2n(cc(I)c2c1)C1CCN(C2CN(C2)C(=O)C=C)C1=O	6.32
BDBM515987	COc1cccc2CN(CCc12)C(=O)c1c(C)n(CC(=O)NC2(C)CN(C2)C(=O)C=C)c2ccc(Br)cc12	6.32
BDBM516156	Cc1cccc2CN(CCc12)C(=O)c1cn(CC(=O)NC2CN(C2)C(=O)C=C)c2ccc(cc12)[N+]([O-])=O	6.32
BDBM544075	CC(C)c1ncccc(C)c1-n1c2nc(c(Cl)cc2c(nc1=O)N1C[C@@H](C)N(C[C@@H]1C)C(=O)C=C)-c1cccc2cn[nH]c12	6.32
BDBM548310	CNC(=O)C[C@H](CC(C)C)Nc1nc(nc2ccoc12)N1CCC2(CN(C2)C(=O)C=C)C1	6.31
BDBM544380	CC(C)c1nc(C)nc(C)c1-n1c2nc(c(C)cc2c(nc1=O)N1C[C@@H](C)N(C[C@@H]1C)C(=O)C=C)-c1ccccc1F	6.31
BDBM515995	Brc1ccc2n(CC(=O)NC3CN(C3)C(=O)C=C)cc(I)c2c1	6.31
CHEMBL4536127	C=CC(=O)N1CCN(c2ncnc3cc(-c4c(C)ccc5[nH]ncc45)c(Cl)cc23)CC1	6.3
BDBM50527056	Cc1ccc2[nH]ncc2c1-c1cc2ncnc(N3CCN(CC3)C(=O)C=C)c2cc1Cl	6.3
BDBM544434	CC(C)c1nccc(C)c1-n1c2nc(c(C)nc2c(nc1=O)N1C[C@@H](C)N(C[C@@H]1C)C(=O)C=C)-c1ccccc1F	6.3
BDBM516078	COc1ccc2ccccc2c1CCNC(=O)c1cn(CC(=O)NC2CN(C2)C(=O)C=C)c2ccc(Br)cc12	6.29
BDBM50517185	Fc1cccc2CN(CCc12)C(=O)c1cn(CC(=O)NC2CN(C2)C(=O)C=C)c2ccc(Br)cc12	6.29
BDBM515939	Cc1c(C(=O)N2CCN(CC2)c2ccc(C)cn2)c2cc(Br)ccc2n1CC(=O)NC1CN(C1)C(=O)C=C	6.28
BDBM515962	C[C@H]1CN(CCN1C(=O)c1c(C)n(CC(=O)N(C)C2CN(C2)C(=O)C=C)c2ccc(Br)cc12)c1ccc(cn1)C#N	6.28
BDBM516069	CN(C)Cc1ccc(nc1)N1CCN(CC1)C(=O)c1cn(CC(=O)NC2CN(C2)C(=O)C=C)c2ccc(Br)cc12	6.28
BDBM515981	Brc1ccc2n(CC(=O)NC3CN(C3)C(=O)C=C)c3ccccc3c2c1	6.28
BDBM516049	Clc1ccc2n(CC(=O)NC3CN(C3)C(=O)C=C)cc(I)c2c1	6.28
BDBM516057	Cc1c(-c2cccc3ncccc23)c2cc(Cl)ccc2n1CC(=O)NC1CN(C1)C(=O)C=C	6.28
BDBM50507455	C[C@@H](CN(C)C)Oc1nc2CN(CCc2c(n1)N1CCN(CC1)C(=O)C=C)c1cc(O)cc2ccccc12	6.27
CHEMBL4465028	C=CC(=O)N1CCN(c2nc(O[C@@H](C)CN(C)C)nc3c2CCN(c2cc(O)cc4ccccc24)C3)CC1	6.27
BDBM535179	Cc1cc2c(ncnc2cc1-c1nc(N)ccc1C(F)(F)F)N1CCN(CC1)C(=O)C=C	6.27
BDBM515974	Cc1c(C(=O)N2CCOC(C)(C)C2)c2cc(Br)ccc2n1CC(=O)NC1CN(C1)C(=O)C=C	6.27
BDBM544340	CCc1nc(C(C)C)c(c(n1)C(C)C)-n1c2nc(c(Cl)cc2c(nc1=O)N1C[C@@H](C)N(C[C@@H]1C)C(=O)C=C)-c1ccccc1F	6.26
BDBM515978	Cc1cccc(C)c1OCCNC(=O)c1cn(CC(=O)NC2CN(C2)C(=O)C=C)c2ccc(Br)cc12	6.25

BDBM50517174	Clc1ccc2CN(CCc2c1)C(=O)c1cn(CC(=O)NC2CN(C2)C(=O)C=C)c2ccc(Br)cc12	6.24
BDBM544144	CC(C)c1nccc(C)c1-n1c2nc(C3CC3)c(Cl)cc2c(nc1=O)N1C[C@@H](C)N(C[C@@H]1C)C(=O)C=C	6.24
BDBM516003	COc1ccc(nc1)N1CCN(CC1)C(=O)c1cn(CC(=O)NC2CN(C2)C(=O)C=C)c2ccc(Br)cc12	6.24
BDBM544224	C[C@@H]1CN([C@@H](C)CN1C(=O)C=C)c1nc(=O)n(CC(C)(C)C)c2nc(c(Cl)cc12)-c1ccccc1F	6.24
BDBM515924	Cc1cc(Cl)cc2c(C(=O)N3CCN(CC3)c3ccc(cn3)C#N)c(C3CC3)n(CC(=O)NC3CN(C3)C(=O)C=C)c12	6.24
BDBM544289	CC(C)c1nccc(C)c1-n1c2nc(c(F)cc2c(nc1=O)N1C[C@@H](C)N(C[C@@H]1C)C(=O)C=C)-c1cc(O)cc2ccccc12	6.24
BDBM544101	CC(C)c1nccc(C)c1-n1c2nc(c(Cl)cc2c(nc1=O)N1C[C@@H](C)N(C[C@@H]1C)C(=O)C=C)-c1cccc(c1)C(F)(F)F	6.23
BDBM516060	Clc1ccccc1-c1ccc2c(I)cn(CC(=O)NC3CN(C3)C(=O)C=C)c2c1	6.23
BDBM535197	Nc1ccc(C(F)F)c(n1)-c1cc2ncnc(N3CCN(CC3)C(=O)C=C)c2cc1Cl	6.23
BDBM516086	Cc1nc(CCNC(=O)c2cn(CC(=O)NC3CN(C3)C(=O)C=C)c3ccc(Br)cc23)sc1C	6.22
BDBM515929	CN(C1CN(C1)C(=O)C=C)C(=O)Cn1c(C2CC2)c(C(=O)N2CCN(CC2)c2ccc(cn2)C#N)c2cc(Cl)cc(C)c12	6.21
BDBM515934	Cc1c(C(=O)N2CCN(CC2)c2ccc(C)cn2)c2cc(Cl)cc(C)c2n1CC(=O)NC1CN(C1)C(=O)C=C	6.21
CHEMBL4454588	C=CC(=O)N1CCN2c3c(cnc4cc(-c5c(C)ccc6[nH]ncc56)c(Cl)cc34)OC[C@@H]2C1	6.21
BDBM50527053	Cc1ccc2[nH]ncc2c1-c1cc2ncc3OC[C@@H]4CN(CCN4c3c2cc1Cl)C(=O)C=C	6.21
BDBM544362	CC(C)c1nc(N)cc(C)c1-n1c2nc(c(Cl)cc2c(cc1=O)N1CCN(C[C@@H]1C)C(=O)C=C)-c1ccccc1F	6.21
BDBM544143	CC(C)c1nccc(C)c1-n1c2nc(C3CCCC3)c(Cl)cc2c(nc1=O)N1C[C@@H](C)N(C[C@@H]1C)C(=O)C=C	6.2
CHEMBL4444447	C=CC(=O)N1CC(NC(=O)Cn2cc(C(=O)N3CCc4c(cccc4OC)C3)c3cc(Br)ccc32)C1	6.2
BDBM50527049	Cc1ccc2[nH]ncc2c1-c1cc2ncc3OCC[C@H]4CN(CCN4c3c2cc1Cl)C(=O)C=C	6.2
BDBM516045	[O-][N+](=O)c1ccc2n(CC(=O)NC3CN(C3)C(=O)C=C)c3CCCCCc3c2c1	6.2
CHEMBL4548586	C=CC(=O)N1CCN2c3c(cnc4cc(-c5c(C)ccc6[nH]ncc56)c(Cl)cc34)OCC[C@H]2C1	6.2
BDBM544024	CC(C)c1nccc(C)c1-n1c2nc(c(F)cc2c(nc1=O)N1C[C@@H](C)N(C[C@@H]1C)C(=O)C=C)-c1ccccc1P(C)(C)=O	6.19
BDBM516080	Brc1ccc2n(CC(=O)NC3CN(C3)C(=O)C=C)cc(C(=O)NCCC(=O)Nc3cccc4ccccc34)c2c1	6.19
BDBM516010	CC(C)(C)OC(=O)N1CCc2c(C1)n(CC(=O)NC1CN(C1)C(=O)C=C)c1ccc(Br)cc21	6.19
BDBM50517240	C[C@@H]1Cc2c(C[C@H]1c1c(C)ccc3[nH]ncc13)ncnc2N1CCN(CC1)C(=O)C=C	6.19
CHEMBL4572411	C=CC(=O)N1CCN(c2ncnc3c2C[C@@H](C)[C@H](c2c(C)ccc4[nH]ncc24)C3)CC1	6.19
BDBM515921	Cc1cc(Cl)cc2c(C(=O)N3CCCC(C)(C)C3)c(C3CC3)n(CC(=O)NC3CN(C3)C(=O)C=C)c12	6.18
BDBM516037	CN(C)C(CNC(=O)c1cn(CC(=O)NC2CN(C2)C(=O)C=C)c2ccc(Br)cc12)c1sccc1C	6.18
BDBM515960	COc1cccc2CN(CCc12)C(=O)c1c(C)n([C@@H]2CCN(C3CN(C3)C(=O)C=C)C2=O)c2ccc(Br)cc12	6.18
BDBM516252	Cc1c(-c2cncnc2C2CCCCC2)c2cc(Cl)cc(C)c2n1CC(=O)NC1CN(C1)C(=O)C=C	6.18
BDBM516026	Cc1c(C(=O)N2CCc3ncccc3C2)c2cc(Br)ccc2n1CC(=O)NC1CN(C1)C(=O)C=C	6.18
BDBM50514376	CC(C)c1ccccc1-c1nnc(N2CCN(CC2)C(=O)C=C)c2cc(Cl)c(cc12)-c1c(O)cccc1F	6.17
BDBM516083	CN(CCNC(=O)c1cn(CC(=O)NC2CN(C2)C(=O)C=C)c2ccc(Br)cc12)c1cccc(C)c1	6.17
BDBM544184	CC(C)c1cc(ccc1N1C(=C)N=C(N2C[C@@H](C)N(C[C@@H]2C)C(=C)C=C)c2cc(Cl)c(nc12)-c1ccccc1F)S(C)=C	6.17
CHEMBL4460809	C=CC(=O)N1CCN(c2nnc(-c3ccccc3C(C)C)c3cc(-c4c(O)cccc4F)c(Cl)cc23)CC1	6.17
BDBM516079	Cc1ccc(C)c(NC(=O)CCNC(=O)c2cn(CC(=O)NC3CN(C3)C(=O)C=C)c3ccc(Br)cc23)c1	6.17
BDBM516059	COc1cccc2CN(CCc12)C(=O)c1cn(CC(=O)NC2CN(C2)C(=O)\C=C\CN(C)C)c2ccc(Br)cc12	6.17
BDBM50517176	Brc1ccc2n(CC(=O)NC3CN(C3)C(=O)C=C)cc(C(=O)NCCc3ccccc3)c2c1	6.17
BDBM516085	Brc1ccc2n(CC(=O)NC3CN(C3)C(=O)C=C)cc(C(=O)On3nnc4cccnc34)c2c1	6.16
BDBM544122	CC(C)c1nccc(C)c1-n1c2nc(c(F)cc2c(nc1=O)N1C[C@@H](C)N(C[C@@H]1C)C(=O)C=C)-c1cc(F)ccc1F	6.16
BDBM548344	CC(C)C[C@@H](Cc1nc(C)no1)Nc1nc(nc2CC(C)(C)CCc12)N1CCC2(CN(C2)C(=O)C=C)C1	6.16
BDBM516013	Brc1ccc2n(CC(=O)NC3CN(C3)C(=O)C=C)cc(C(=O)N3CCO[C@@H](C3)c3ccccc3)c2c1	6.15

BDBM548365	CNC(=O)C[C@H](CC(C)C)Nc1nc(nc2CCCCc12)N1CCC2(CN(C2)C(=O)C=C)C1	6.15
BDBM544429	CC(C)c1nccc(C)c1-n1c2nc(c(Cl)cc2c(nc1=O)N1CCN(C2CC12)C(=O)C=C)-c1ccccc1F	6.15
BDBM544124	CC(C)c1nccc(C)c1-n1c2nc(c(F)cc2c(nc1=O)N1C[C@@H](C)N(C[C@@H]1C)C(=O)C=C)-c1cc(Cl)ccc1F	6.15
BDBM516022	Clc1ccc2n(CC(=O)NC3CN(C3)C(=O)C=C)cc(-c3ccccc3-c3ccccc3)c2c1	6.15
BDBM515959	Cc1c(-c2ccccc2N2CCOCC2)c2cc(Cl)ccc2n1CC(=O)NC1CN(C1)C(=O)C=C	6.14
BDBM515897	Cc1cc(Cl)cc2c(C(=O)N3CCOC4(CCC4)C3)c(C3CC3)n(CC(=O)NC3CN(C3)C(=O)C=C)c12	6.14
BDBM515998	CN(C)Cc1ccccc1CNC(=O)c1cc2cc(Br)ccc2n1CC(=O)NC1CN(C1)C(=O)C=C	6.14
BDBM548342	CNC(=O)C[C@H](CC(C)C)Nc1nc(nc2cccnc12)N1CCC2(CN(C2)C(=O)C=C)C1	6.13
BDBM544137	CNC(=O)c1ccccc1-c1nc2n(-c3c(C)ccnc3C(C)C)c(=O)nc(N3C[C@@H](C)N(C[C@@H]3C)C(=O)C=C)c2cc1F	6.13
BDBM516056	Clc1ccccc1-c1ccc2c(cn(CC(=O)NC3CN(C3)C(=O)C=C)c2c1)C1CC1	6.13
BDBM535203	Nc1ccc(c(n1)-c1cc2ncnc(N3CCN([C@@H](C3)C#N)C(=O)C(F)=C)c2cc1Cl)C(F)(F)F	6.12
BDBM544156	CC(C)c1ncnc(C(C)C)c1-n1c2nc(c(Cl)cc2c(nc1=O)N1C[C@@H](C)N(C[C@@H]1C)C(=O)C=C)-c1ccccc1C#N	6.12
BDBM544080	CC(C)c1nccc(C)c1-n1c2nc(c(Cl)cc2c(nc1=O)N1C[C@@H](C)N(C[C@@H]1C)C(=O)C=C)-c1ccccc1NC(=O)C(C)(C)C	6.12
BDBM516154	CCc1ccccc1NC(=O)CCNC(=O)c1cn(CC(=O)NC2CN(C2)C(=O)C=C)c2ccc(cc12)[N+]([O-])=O	6.12
BDBM516054	Oc1cccc(F)c1-c1cn(CC(=O)NC2CN(C2)C(=O)C=C)c2ccc(Cl)cc12	6.12
BDBM535209	COc1cc2c(ncnc2cc1-c1nc(N)ccc1C1CC1)N1CCN(CC1)C(=O)C=C	6.12
BDBM50517177	CN(Cc1ccccc1)C(=O)c1cn(CC(=O)NC2CN(C2)C(=O)C=C)c2ccc(Br)cc12	6.11
BDBM548346	CNC(=O)C[C@H](CC(C)C)Nc1nc(nc2ccc(C)cc12)N1CCC2(CN(C2)C(=O)C=C)C1	6.11
BDBM516221	COc1cccc2CN(CCc12)C(=O)c1c(C)n(C(C)C(=O)N(C)C2CN(C2)C(=O)C=C)c2ccc(Cl)cc12	6.11
BDBM544439	CC(C)c1nccc(C)c1-n1c2nc(c(Cl)cc2c(nc1=O)N1CCN(C[C@@H]1C)C(=O)C=C)-c1cccc2NC(=O)Cc12	6.11
BDBM515912	CC(C)c1ccncc1-c1c(C)n(CC(=O)NC2CN(C2)C(=O)C=C)c2ccc(Cl)cc12	6.11
BDBM516245	Cc1c(-c2cnccc2C2=CCCCC2)c2cc(Cl)cc(C)c2n1CC(=O)NC1CN(C1)C(=O)C=C	6.1
BDBM548352	CC(C)C[C@@H](Cn1ccnn1)Nc1nc(nc2CC(C)(C)CCc12)N1CCC2(CN(C2)C(=O)C=C)C1	6.09
BDBM515920	CN(C1CN(C1)C(=O)C=C)C(=O)Cn1c(C)c(C(=O)N2CCN(CC2)c2ccc(cn2)C(F)(F)F)c2cc(Br)ccc12	6.09
BDBM516215	Cc1c(-c2cccnc2Br)c2cc(Cl)ccc2n1CC(=O)NC1CN(C1)C(=O)C=C	6.09
BDBM515969	CCC1N(CCc2ccccc12)C(=O)c1c(C)n(CC(=O)NC2CN(C2)C(=O)C=C)c2c(C)cc(Br)cc12	6.09
BDBM535172	Cc1cc(N)nc(-c2cc3ncnc(N4CCN(C(C4)C#N)C(=O)C4CO4)c3cc2Cl)c1C(F)(F)F	6.09
BDBM548326	CC(C)C[C@@H](CC1=NN=[c](C)[o]1)Nc1nc(nc2CCCCc12)N1CCC2(CN(C2)C(=O)C=C)C1	6.08
BDBM516052	Cc1ccc2[nH]ncc2c1-c1cn(CC(=O)NC2CN(C2)C(=O)C=C)c2ccc(Cl)cc12	6.08
BDBM544146	CCCc1nc2n(-c3c(C)ccnc3C(C)C)c(=O)nc(N3C[C@@H](C)N(C[C@@H]3C)C(=O)C=C)c2cc1Cl	6.08
BDBM516064	Clc1cn(CC(=O)NC2CN(C2)C(=O)C=C)c2ccc(Br)cc12	6.07
BDBM516053	[O-][N+](=O)c1ccc2n(CC(=O)NC3CN(C3)C(=O)C=C)c3CCCCc3c2c1	6.07
BDBM544130	CC(C)c1nccc(C)c1-n1c2nc(c(F)cc2c(nc1=O)N1C[C@@H](C)N(C[C@@H]1C)C(=O)C=C)-c1c(F)cccc1F	6.07
BDBM535212	Nc1ccc(c(n1)-c1cc2ncnc(C3CN(C3)C(=O)C=C)c2cc1Cl)C(F)(F)F	6.06
CHEMBL4788500	O=C1N[C@H](c2c(CNCc3ccc4ccn(Cc5ccccc5)c4c3)[nH]c3ccccc23)c2cc(O)ccc21	6.06
BDBM535219	CNc1ccc(c(n1)-c1cc2ncnc(N3CCN(CC3)C(=O)C=C)c2cc1Cl)C(F)(F)F	6.06
BDBM515966	Cc1c(-c2ccccc2Cc2ccccc2)c2cc(Cl)ccc2n1CC(=O)NC1CN(C1)C(=O)C=C	6.06
BDBM516254	CC1CCN(CC1)c1ncccc1-c1c(C)n(CC(=O)NC2CN(C2)C(=O)C=C)c2c(C)cc(Cl)cc12	6.06
BDBM50549224	Oc1ccc2C(=O)N[C@@H](c2c1)c1c(CNCc2ccc3ccn(Cc4ccccc4)c3c2)[nH]c2ccccc12	6.06
BDBM516048	Brc1ccc2n(CC(=O)NC3CN(C3)C(=O)C=C)cc(C(=O)N3CCOC(C3)c3ccccc3)c2c1	6.05
BDBM516023	CC(C)Oc1ccccc1-c1c(C)n(CC(=O)NC2CN(C2)C(=O)C=C)c2ccc(Cl)cc12	6.05

BDBM516250	Cc1c(-c2cnccc2C2=CCCCCC2)c2cc(Cl)cc(C)c2n1CC(=O)NC1CN(C1)C(=O)C=C	6.05
BDBM535125	Nc1cc2c(Cl)cccc2c(n1)-c1cc2ncnc(N3CCN(CC3)C(=O)C=C)c2cc1Cl	6.05
BDBM516012	Brc1ccc2n(CC(=O)NC3CN(C3)C(=O)C=C)c3CCCCCc3c2c1	6.05
BDBM516061	COc1ccccc1-c1cn(CC(=O)NC2CN(C2)C(=O)C=C)c2ccc(Br)cc12	6.04
BDBM515997	CCc1cc(Br)cc2c(I)cn(CC(=O)NC3CN(C3)C(=O)C=C)c12	6.04
BDBM548313	CNC(=O)C[C@H](CC(C)C)Nc1nc(nc2cn(C)nc12)N1CCC2(CN(C2)C(=O)C=C)C1	6.04
BDBM548319	CNC(=O)C[C@H](CC(C)C)Nc1nc(nc2n(C)ccc12)N1CCC2(CN(C2)C(=O)C=C)C1	6.04
BDBM50514375	CCc1ccccc1-c1nnc(N2CCN(CC2)C(=O)C=C)c2cc(Cl)c(cc12)-c1c(O)cccc1F	6.04
CHEMBL4515333	C=CC(=O)N1CCN(c2nnc(-c3ccccc3CC)c3cc(-c4c(O)cccc4F)c(Cl)cc23)CC1	6.04
BDBM516082	Cc1csc(CCCNC(=O)c2cn(CC(=O)NC3CN(C3)C(=O)C=C)c3ccc(Br)cc23)n1	6.03
BDBM544396	C[C@@H]1CN([C@@H](C)CN1C(=O)C=C)C1=NC(=O)CN(CC(C)(C)C)c2nc(c(Cl)cc12)-c1ccccc1F	6.03
BDBM515954	COc1cccc2CN(CCc12)C(=O)c1c(C)n(CC(=O)NC2CN(C2C)C(=O)C=C)c2ccc(Br)cc12	6.02
BDBM515970	Brc1ccc2n(CC(=O)NC3CN(C3)C(=O)C=C)cc(C(=O)N3CCN(CC3)c3ccccn3)c2c1	6.02
BDBM544134	CC(C)c1nccc(C)c1-n1c2nc(c(F)cc2c(nc1=O)N1C[C@@H](C)N(C[C@@H]1C)C(=O)C=C)-c1ccccc1C(O)=O	6.02
BDBM515933	Cc1cc(Cl)cc2c(C(=O)N3CCOC(C)(C)C3)c(C3CC3)n(CC(=O)NC3CN(C3)C(=O)C=C)c12	6.02
BDBM544427	CC(C)c1nccc(C)c1-n1c2nc(c(Cl)cc2c(nc1=O)N1CCN([C@H]2C[C@@H]12)C(=O)C=C)-c1ccccc1F	6.02
BDBM516167	FC(F)(F)c1ccc2n(CC(=O)NC3CN(C3)C(=O)C=C)cc(I)c2c1	6.01
BDBM544053	CC(C)c1nccc(C)c1-n1c2nc(C=C(C)C)c(Cl)cc2c(nc1=O)N1C[C@@H](C)N(C[C@@H]1C)C(=O)C=C	6.01
BDBM515928	Cc1c(-c2cnccc2C2CC2)c2cc(Cl)ccc2n1CC(=O)NC1CN(C1)C(=O)C=C	6.01
BDBM544126	CC(C)c1nccc(C)c1-n1c2nc(c(F)cc2c(nc1=O)N1C[C@@H](C)N(C[C@@H]1C)C(=O)C=C)-c1cc(ccc1F)C1CC1	6.01
BDBM544241	CC(C)c1nc(N)cc(C)c1-n1c2nc(c(F)cc2c(nc1=O)N1C[C@H](C)N([C@H](C)C1)C(=O)C=C)-c1ccccc1F	6
BDBM544194	C[C@H]1CN(CCN1c1nc(=O)n(CC(C)(C)C)c2nc(c(Cl)cc12)-c1ccccc1F)C(=O)C=C	6
BDBM535185	Nc1cc(C2CC2)c(c(n1)-c1cc2ncnc(N3CCN(CC3)C(=O)C=C)c2cc1Cl)C(F)(F)F	6
BDBM50539755	CN1CCC[C@H]1COc1nc2CN(CCc2c(n1)N1CCN([C@H](CC#N)C1)C(=O)C=C)c1cccc2ccccc12	5.99
BDBM516040	Cc1ccc2cn[nH]c2c1-c1cn(CC(=O)NC2CN(C2)C(=O)C=C)c2ccc(Br)cc12	5.99
BDBM515991	C[C@H]1CN(CCN1C(=O)c1c(C)n(CC(=O)NC2CN(C2)C(=O)C=C)c2ccc(Br)cc12)c1ccc(cn1)C#N	5.99
CHEMBL4649703	C=CC(=O)N1CCN(c2nc(OC[C@@H]3CCCN3C)nc3c2CCN(c2cccc4ccccc24)C3)C[C@H]1CC#N	5.99
BDBM544337	CCc1cc(C)c(c(n1)C(C)C)-n1c2nc(c(Cl)cc2c(nc1=O)N1C[C@H](C)N(C[C@@H]1C)C(C)=O)-c1ccccc1F	5.99
BDBM516209	Cc1c(C(=O)NCCc2ccccc2)c2cc(Br)ccc2n1CC(=O)NC1CN(C1)C(=O)C=C	5.98
BDBM544142	CC(C)c1nccc(C)c1-n1c2nc(CC(C)(C)C)c(Cl)cc2c(nc1=O)N1C[C@@H](C)N(C[C@@H]1C)C(=O)C=C	5.98
BDBM515913	CC(C)c1ncncc1-c1c(C)n(CC(=O)NC2CN(C2)C(=O)C=C)c2ccc(Cl)cc12	5.98
BDBM515958	Cc1cccc(n1)N1CCN(CC1)C(=O)c1cn(CC(=O)NC2CN(C2)C(=O)C=C)c2ccc(Br)cc12	5.98
BDBM516081	Brc1ccc2n(CC(=O)NC3CN(C3)C(=O)C=C)cc(C(=O)NCCc3nc4CCCCc4s3)c2c1	5.98
BDBM516041	COc1cccc2CN(CCc12)C(=O)c1c(C)n(c2ccc(Cl)cc12)C(C)(C)C(=O)NC1CN(C1)C(=O)C=C	5.98
CHEMBL4637314	C=CC(=O)N1CCN(c2nc(OC[C@@H]3CCCN3C)nc3c2CCN(c2c(CC)ccc4[nH]ncc24)C3)CC1	5.97
BDBM516070	Brc1ccc2n(CC(=O)NC3CN(C3)C(=O)C=C)c3CCCCc3c2c1	5.97
BDBM50539744	CCc1ccc2[nH]ncc2c1N1CCc2c(C1)nc(OC[C@@H]1CCCN1C)nc2N1CCN(CC1)C(=O)C=C	5.97
BDBM515955	Cc1ccc(nc1)N1CCN(CC1)C(=O)c1cn(CC(=O)NC2CN(C2)C(=O)C=C)c2ccc(Br)cc12	5.97
BDBM548311	CNC(=O)C[C@H](CC(C)C)Nc1nc(nc2[C@H](C)CCCc12)N1CCC2(CN(C2)C(=O)C=C)C1	5.96
BDBM544095	CC(C)c1nccc(C)c1-n1c2nc(-c3ccsc3)c(Cl)cc2c(nc1=O)N1C[C@@H](C)N(C[C@@H]1C)C(=O)C=C	5.96
BDBM516005	Cc1cc2n(CC(=O)NC3CN(C3)C(=O)C=C)cc(I)c2cc1Br	5.96

BDBM548308	CNC(=O)C[C@H](CC(C)C)Nc1nc(nc2ccccc12)N1CCC2(CN(C2)C(=O)C=C)C1	5.96
BDBM516011	Cc1c(-c2cccc3nc(C)ccc23)c2cc(Cl)ccc2n1CC(=O)NC1CN(C1)C(=O)C=C	5.95
BDBM516089	CCc1ccccc1NC(=O)CCNC(=O)c1c(C)n(CC(=O)NC2CN(C2)C(=O)C=C)c2ccc(Br)cc12	5.95
BDBM516074	Brc1ccc2n(CC(=O)NC3CN(C3)C(=O)C=C)c(cc2c1)C(=O)NCc1ccccc1	5.95
BDBM516008	CN(C)c1ccccc1-c1c(C)n(CC(=O)NC2CN(C2)C(=O)C=C)c2ccc(Cl)cc12	5.95
BDBM544261	CC(C)c1nccc(C)c1-n1c2nc(c(F)cc2c(nc1=O)N1CCN(C(C)C1C)C(=O)C=C)-c1c(O)cccc1F	5.95
BDBM516055	Brc1ccc2n(CC(=O)NC3CN(C3)C(=O)C=C)cc(CN3CCc4ccccc4C3)c2c1	5.95
BDBM516047	Fc1ccc(-c2cn(CC(=O)NC3CN(C3)C(=O)C=C)c3ccc(Br)cc23)c(F)c1	5.95
BDBM544019	CC(C)c1nccc(C)c1-n1c2nc(N3CCCCC3)c(Cl)cc2c(nc1=O)N1C[C@@H](C)N(C[C@@H]1C)C(=O)C=C	5.94
BDBM544298	CC(C)(C)Cn1c2nc(c(Cl)cc2c(nc1=O)N1CCN(C(CC#N)C1)C(=O)C=C)-c1ccccc1F	5.94
BDBM544347	CC(C)c1nc(cc(C)c1-n1c2nc(c(Cl)cc2c(nc1=O)N1C[C@@H](C)N(C[C@@H]1C)C(=O)C=C)-c1ccccc1F)N1CCC1	5.94
BDBM548327	CC(C)C[C@@H](Cc1ncc[nH]1)Nc1nc(nc2CCCCc12)N1CCC2(CN(C2)C(=O)C=C)C1	5.94
CHEMBL4642347	C=CC(=O)N1CCN(c2nc(OC[C@@H]3CCCN3C)nc3c2CCN(c2c(C(C)C)ccc4[nH]ncc24)C3)CC1	5.93
BDBM515941	Cc1cc(Cl)cc2c(C(=O)N3CCOC(C)(C3)c3ccccc3)c(C3CC3)n(CC(=O)NC3CN(C3)C(=O)C=C)c12	5.93
BDBM516038	[O-][N+](=O)c1ccc2n(CC(=O)NC3CN(C3)C(=O)C=C)cc(I)c2c1	5.93
BDBM50539745	CC(C)c1ccc2[nH]ncc2c1N1CCc2c(C1)nc(OC[C@@H]1CCCN1C)nc2N1CCN(CC1)C(=O)C=C	5.93
BDBM515988	Cc1ccccc1-c1cn(CC(=O)NC2CN(C2)C(=O)C=C)c2ccc(Cl)cc12	5.92
BDBM535144	Cc1cc(N)nc(c1F)-c1cc2ncnc(N3CCN(CC3)C(=O)C=C)c2cc1Cl	5.92
BDBM516020	Cc1cc(Br)cc2c(cn(CC(=O)NC3CN(C3)C(=O)C=C)c12)-c1cccc2ccccc12	5.92
BDBM544167	CC(C)c1ncnc(C(C)C)c1-n1c2nc(c(Cl)cc2c(nc1=O)N1CCN(C[C@@H]1C)C(=O)\C=C\CN(C)C)-c1ccccc1F	5.91
BDBM516214	Brc1ccc2n(CC(=O)NC3CN(C3)C(=O)C=C)c(C#N)c(I)c2c1	5.91
BDBM544149	COc1cccnc1-c1nc2n(-c3c(C)ccnc3C(C)C)c(=O)nc(N3C[C@@H](C)N(C[C@@H]3C)C(=O)C=C)c2cc1Cl	5.91
BDBM516258	CN(C1CN(C1)C(=O)C=C)C(=O)Cn1c(C)cc2cc(Br)ccc12	5.91
BDBM544426	CC(C)c1nccc(C)c1-n1c2nc(c(Cl)cc2c(N2CCN(CC2)C(=O)C=C)c(C(N)=O)c1=O)-c1ccccc1F	5.9
BDBM515930	Cc1cc(Cl)cc2c(C(=O)N3CCC(C)(C)C3)c(C3CC3)n(CC(=O)NC3CN(C3)C(=O)C=C)c12	5.9
CHEMBL4476533	C=CC(=O)N1CC(NC(=O)Cn2cc(C(=O)N3CCc4ccccc4C3)c3cc(Br)ccc32)C1	5.9
BDBM544352	CC(C)c1nc(C=C)cc(C)c1-n1c2nc(c(Cl)cc2c(nc1=O)N1C[C@H](C)N(C[C@@H]1C)C(=O)C=C)-c1ccccc1F	5.9
BDBM544431	CC(C)c1nccc(C)c1-n1c2nc(c(Cl)nc2c(nc1=O)N1C[C@H](C)N([C@H](C)C1)C(=O)C=C)-c1ccccc1F	5.89
CHEMBL4570206	C=CC(=O)N1CC(NC(=O)Cn2cc(C(=O)N3CCc4c(Cl)cccc4C3)c3cc(Br)ccc32)C1	5.89
BDBM516043	Brc1ccc2n(CC(=O)NC3CN(C3)C(=O)C=C)c3CCC(Cc3c2c1)c1ccccc1	5.88
BDBM548355	CNC(=O)C[C@H](Cc1ccccc1)Nc1nc(nc2CC(C)(C)CCc12)N1CCC2(CN(C2)C(=O)C=C)C1	5.88
BDBM516150	[O-][N+](=O)c1cn(CC(=O)NC2CN(C2)C(=O)C=C)c2ccc(Cl)cc12	5.88
BDBM544116	CC(C)c1nccc(C)c1-n1c2nc(-c3nc(C)sc3C)c(Cl)cc2c(nc1=O)N1C[C@@H](C)N(C[C@@H]1C)C(=O)C=C	5.87
BDBM544114	CC(C)c1nccc(C)c1-n1c2nc(c(Cl)cc2c(nc1=O)N1C[C@@H](C)N(C[C@@H]1C)C(=O)C=C)-c1cccnc1C1CC1	5.87
BDBM516210	Cc1c(C(=O)N2CCc3cccnc3C2)c2cc(Br)ccc2n1CC(=O)NC1CN(C1)C(=O)C=C	5.87
BDBM544401	CC(C)c1cc(ccc1-n1c2nc(c(Cl)cc2c(nc1=O)N1C[C@H](C)N(C[C@@H]1C)C(=O)C=C)-c1ccccc1F)S(C)=O	5.87
BDBM515989	Cc1ccnc(c1)N1CCN(CC1)C(=O)c1cn(CC(=O)NC2CN(C2)C(=O)C=C)c2ccc(Br)cc12	5.86
CHEMBL4456127	C=CC(=O)N1CC(NC(=O)Cn2cc(C(=O)N3CCc4cc(F)ccc4C3)c3cc(Br)ccc32)C1	5.86
BDBM535147	Cc1cc(N)nc(c1F)-c1cc2nc(NC[C@@H](F)C(C)(C)O)nc(N3CCN(CC3)C(=O)C=C)c2cc1Cl	5.85
BDBM516257	Cc1cc2cc(Br)ccc2n1CC(=O)NC1CN(C1)C(=O)C=C	5.83
BDBM548350	CC(C)C[C@@H](Cn1cncn1)Nc1nc(nc2CC(C)(C)CCc12)N1CCC2(CN(C2)C(=O)C=C)C1	5.82

BDBM516051	Brc1ccc2n(CC(=O)NC3CN(C3)C(=O)C=C)c3CCN(Cc3c2c1)C(=O)OCc1ccccc1	5.82
CHEMBL4525173	C=CC(=O)N1CCN(c2nc(OCCCN(C)C)nc3c2CCN(c2cc(O)cc4ccccc24)C3)CC1	5.82
BDBM50507452	CN(C)CCCOc1nc2CN(CCc2c(n1)N1CCN(CC1)C(=O)C=C)c1cc(O)cc2ccccc12	5.82
CHEMBL4637434	C=CC(=O)N1CCN(c2nc(OC[C@@H]3CCCN3C)nc3c2CCN(c2c(C)ccc4[nH]ncc24)C3)CC1	5.82
BDBM50539743	CN1CCC[C@H]1COc1nc2CN(CCc2c(n1)N1CCN(CC1)C(=O)C=C)c1c(C)ccc2[nH]ncc12	5.82
BDBM516260	CN(C1CN(C1)C(=O)C=C)C(=O)Cn1ccc2c(C)c(Br)ccc12	5.82
BDBM50514398	Oc1cccc(F)c1-c1cc2c(nnc(N3CCN(CC3)C(=O)C=C)c2cc1Cl)-c1ccccc1C1CC1	5.81
BDBM516235	CC(C)c1ccncc1-c1c(C)n(CC(=O)NC2CN(C2)C(=O)C=C)c2c(C)cc(Cl)cc12	5.81
CHEMBL4553629	C=CC(=O)N1CCN(c2nnc(-c3ccccc3C3CC3)c3cc(-c4c(O)cccc4F)c(Cl)cc23)CC1	5.81
BDBM515936	CC1COCC(C)N1C(=O)c1c(C)n(CC(=O)NC2CN(C2)C(=O)C=C)c2c(C)cc(Br)cc12	5.8
BDBM515971	Cc1cccnc1N1CCN(CC1)C(=O)c1cn(CC(=O)NC2CN(C2)C(=O)C=C)c2ccc(Br)cc12	5.8
CHEMBL4476113	C=CC(=O)N1CC(N2CCN(C(=O)CNc3cc(C4(C)CC4)c(Cl)cc3O)CC2)C1	5.8
BDBM50503649	CC1(CC1)c1cc(NCC(=O)N2CCN(CC2)C2CN(C2)C(=O)C=C)c(O)cc1Cl	5.8
BDBM516143	[O-][N+](=O)c1ccc2n(CC(=O)NC3CN(C3)C(=O)C=C)cc(Br)c2c1	5.8
BDBM516213	C[C@@H]1CN(CCO1)C(=O)c1c(C)n(CC(=O)NC2CN(C2)C(=O)C=C)c2ccc(Br)cc12	5.8
BDBM516216	Cc1c(-c2ncccc2Cl)c2cc(Cl)ccc2n1CC(=O)NC1CN(C1)C(=O)C=C	5.79
BDBM516099	Brc1ccc2n(CC(=O)NC3CN(C3)C(=O)C=C)c(cc2c1)C(=O)NCCc1ccccc1	5.79
BDBM516178	CC(C)c1cccc(c1)-c1cn(CC(=O)NC2CN(C2)C(=O)C=C)c2c(C)cc(Br)cc12	5.79
BDBM544335	CC(C)c1nc(C)cc(C)c1-n1c2nc(c(Cl)cc2c(nc1=O)N1C[C@H](C)N(C[C@@H]1C)C(=O)C=C)-c1ccccc1F	5.79
BDBM516219	C[C@H]1CN(CCO1)C(=O)c1c(C)n(CC(=O)NC2CN(C2)C(=O)C=C)c2ccc(Br)cc12	5.79
BDBM516136	Brc1ccc2n(CC(=O)NC3CN(C3)C(=O)C=C)cc(CC(=O)NCc3ccccc3)c2c1	5.78
BDBM516084	Cn1c(CCNC(=O)c2cn(CC(=O)NC3CN(C3)C(=O)C=C)c3ccc(Br)cc23)nc2ccccc12	5.78
BDBM516042	Brc1ccc2n(CC(=O)NC3CN(C3)C(=O)C=C)cc(C3CC3)c2c1	5.77
BDBM516033	Brc1ccc2n(CC(=O)NC3CN(C3)C(=O)C=C)cc(C(=O)NCCc3nccs3)c2c1	5.77
BDBM515980	Brc1ccc2c(I)cn(CC(=O)NC3CN(C3)C(=O)C=C)c2c1	5.77
BDBM516265	CN(C1CN(C1)C(=O)C=C)C(=O)Cn1cc(C)c2cc(Br)ccc12	5.77
BDBM516100	Cc1cc(Br)cc2ccn(CC(=O)NC3CN(C3)C(=O)C=C)c12	5.75
BDBM516208	Cc1ncc2CN(CCn12)C(=O)c1c(C)n(CC(=O)NC2CN(C2)C(=O)C=C)c2ccc(Br)cc12	5.75
BDBM516151	Brc1ccc2n(CC(=O)NC3CN(C3)C(=O)C=C)cc(C(=O)C(=O)NCc3ccccc3)c2c1	5.75
BDBM516195	OC[C@H](Cc1ccccc1)NC(=O)c1cn(CC(=O)NC2CN(C2)C(=O)C=C)c2ccc(Br)cc12	5.74
BDBM516199	CC(C)C(CNC(=O)c1cn(CC(=O)NC2CN(C2)C(=O)C=C)c2ccc(Br)cc12)c1ccccc1	5.73
BDBM516046	Brc1ccc2n(CC(=O)NC3CN(C3)C(=O)C=C)cc(CNCc3ccccc3)c2c1	5.72
BDBM516130	COC(=O)c1cc2c(Cl)c(Cl)ccc2n1CC(=O)NC1CN(C1)C(=O)C=C	5.72
BDBM50517175	Brc1ccc2n(CC(=O)NC3CN(C3)C(=O)C=C)cc(C(=O)NCc3ccccc3)c2c1	5.72
BDBM50507451	CN(C)CCOc1nc2CN(CCc2c(n1)N1CCN(CC1)C(=O)C=C)c1cc(O)cc2ccccc12	5.72
CHEMBL4550990	C=CC(=O)N1CCN(c2nc(OCCN(C)C)nc3c2CCN(c2cc(O)cc4ccccc24)C3)CC1	5.72
BDBM516193	COC(=O)[C@H](Cc1ccccc1)NC(=O)c1cn(CC(=O)NC2CN(C2)C(=O)C=C)c2ccc(Br)cc12	5.71
BDBM544421	CC(C)c1cc(cc(C)c1-n1c2nc(c(Cl)cc2c(nc1=O)N1C[C@H](C)N(C[C@@H]1C)C(=O)C=C)-c1ccccc1F)S(C)=O	5.7
CHEMBL4435858	C=CC(=O)N1CC(NC(=O)Cn2cc(C(=O)N3CCc4cc(Cl)ccc4C3)c3cc(Br)ccc32)C1	5.7
BDBM516247	CN(C1CN(C1)C(=O)C=C)C(=O)Cn1c(C)c(-c2cnccc2C2=CCCCC2)c2cc(Cl)cc(C)c12	5.69
BDBM516141	Brc1ccc2n(CC(=O)NC3CN(C3)C(=O)C=C)cc(Cc3ccccc3)c2c1	5.69

BDBM516232	CN1CCCC2(CCCN(C2)C(=O)Cc2cn(CC(=O)NC3CN(C3)C(=O)C=C)c3ccc(Br)cc23)C1	5.68
BDBM548362	CC(C)C[C@@H](Cc1ncco1)Nc1nc(nc2CCCCc12)N1CCC2(CN(C2)C(=O)C=C)C1	5.68
BDBM516246	Cc1c(-c2ccccc2Cc2ccccc2)c2cc(Cl)cc(C)c2n1CC(=O)NC1CN(C1)C(=O)C=C	5.68
BDBM535191	Clc1cc2c(ncnc2cc1-c1nc(NCC#N)cc2ccccc12)N1CCN(CC1)C(=O)C=C	5.68
BDBM515950	Cc1c(C(=O)N2CCN(CC2)c2ccc(cn2)C(F)(F)F)c2cc(Br)ccc2n1CC(=O)NC1CN(C1)C(=O)C=C	5.68
BDBM516201	CCC(Cc1ccccc1)NC(=O)c1cn(CC(=O)NC2CN(C2)C(=O)C=C)c2ccc(Br)cc12	5.67
CHEMBL4466759	C=CC(=O)N1CC(NC(=O)Cn2cc(C(=O)N3CCc4c(F)cccc4C3)c3cc(Br)ccc32)C1	5.67
BDBM516218	CC(C)[C@H]1CN(CCO1)C(=O)c1c(C)n(CC(=O)NC2CN(C2)C(=O)C=C)c2ccc(Br)cc12	5.67
BDBM516171	CN(C1CN(C1)C(=O)C=C)C(=O)Cn1c2ccccc2c2cc(Br)ccc12	5.66
BDBM516180	Brc1ccc2n(CC(=O)NC3CN(C3)C(=O)C=C)cc(C(=O)NCCn3cccc3)c2c1	5.66
BDBM516177	Fc1cc(Br)cc2ccn(CC(=O)NC3CN(C3)C(=O)C=C)c12	5.65
BDBM516104	Cc1cn(CC(=O)NC2CN(C2)C(=O)C=C)c2ccc(Br)cc12	5.65
BDBM516273	C[C@H]1[C@H](CN1C(=O)C=C)NC(=O)Cn1ccc2cc(Br)ccc12	5.65
BDBM516163	CC(O)(C(=O)NCc1ccccc1)c1cn(CC(=O)NC2CN(C2)C(=O)C=C)c2ccc(Br)cc12	5.64
BDBM515940	Cc1c(-c2cncnc2C2CC2)c2cc(Cl)ccc2n1CC(=O)NC1CN(C1)C(=O)C=C	5.63
BDBM516191	Cc1c(-c2nccnc2Cl)c2cc(Cl)ccc2n1CC(=O)NC1CN(C1)C(=O)C=C	5.63
BDBM548307	CNC(=O)C[C@H](CC(C)C)Nc1nc(nc2CCCc12)N1CCC2(CN(C2)C(=O)C=C)C1	5.63
BDBM544355	CC(C)c1cnc(C)c(C)c1-n1c2nc(c(Cl)cc2c(nc1=O)N1C[C@H](C)N(C[C@H]1C)C(=O)C=C)-c1ccccc1F	5.62
BDBM516234	CC(C)c1ccncc1-c1c(C2CC2)n(CC(=O)NC2CN(C2)C(=O)C=C)c2c(C)cc(Cl)cc12	5.62
BDBM544350	CC(C)c1cc(C)c(c(n1)C(C)C)-n1c2nc(c(Cl)cc2c(nc1=O)N1C[C@H](C)N(C[C@@H]1C)C(=O)C=C)-c1ccccc1F	5.61
BDBM516251	Cc1c(-c2cnccc2C2=CCOCC2)c2cc(Cl)cc(C)c2n1CC(=O)NC1CN(C1)C(=O)C=C	5.61
BDBM516162	CC(=O)N1CCc2c(C1)n(CC(=O)NC1CN(C1)C(=O)C=C)c1ccc(Br)cc21	5.6
BDBM516137	Brc1ccc2n(CC(=O)NC3CN(C3)C(=O)C=C)c3CN(CCc3c2c1)C(=O)OCc1ccccc1	5.58
BDBM516075	Clc1cc(Cl)c2n(CC(=O)NC3CN(C3)C(=O)C=C)ccc2c1	5.58
BDBM516133	Brc1nnc2CN(CCn12)C(=O)c1cn(CC(=O)NC2CN(C2)C(=O)C=C)c2ccc(Br)cc12	5.58
BDBM50517183	Brc1ccc(OCC(=O)NC2CN(C2)C(=O)C=C)c(c1)C(=O)N1CC=C(C1)c1ccccc1	5.56
CHEMBL4577812	C=CC(=O)N1CC(NC(=O)COc2ccc(Br)cc2C(=O)N2CC=C(c3ccccc3)C2)C1	5.56
BDBM516025	CC(C)Oc1ncccc1-c1c(C)n(CC(=O)NC2CN(C2)C(=O)C=C)c2ccc(Br)cc12	5.56
BDBM516256	CN(C1CN(C1)C(=O)C=C)C(=O)Cn1ccc2cc(Br)ccc12	5.56
BDBM516174	Cc1cc(Br)cc2c(cn(CC(=O)NC3CN(C3)C(=O)C=C)c12)-c1ccc2ccccc2c1	5.56
BDBM516035	COc1ccccc1-c1c(C)n(CC(=O)NC2CN(C2)C(=O)C=C)c2ccc(Cl)cc12	5.56
BDBM516153	Brc1ccc2n(CC(=O)NC3CN(C3)C(=O)C=C)c(cc2c1)-c1ccccc1	5.55
BDBM516134	Brc1ccc2n(CC(=O)NC3CN(C3)C(=O)C=C)cc(C(=O)N3CCN4CCCC[C@@H]4C3)c2c1	5.55
BDBM544364	CC(C)c1cc(CN(C)C)cc(C)c1-n1c2nc(c(F)cc2c(nc1=O)N1C[C@@H](C)N(C[C@@H]1C)C(=O)C=C)-c1c(N)cccc1F	5.55
BDBM516116	Brc1ccc2n(CC(=O)NC3CN(C3)C(=O)C=C)cc(-c3cccnc3)c2c1	5.55
BDBM515975	Cc1c(C(=O)C2CCc3ccccc3C2)c2cc(Br)ccc2n1CC(=O)NC1CN(C1)C(=O)C=C	5.54
BDBM516248	CC(C)c1ncncc1-c1c(C)n(CC(=O)NC2CN(C2)C(=O)C=C)c2c(C)cc(Cl)cc12	5.54
BDBM516129	Clc1ccccc1-c1ccc2c(cn(CC(=O)NC3CN(C3)C(=O)C=C)c2c1)-c1cocn1	5.54
BDBM516249	CC(C)c1ncncc1-c1c(C)n(CC(=O)N(C)C2CN(C2)C(=O)C=C)c2c(C)cc(Cl)cc12	5.54
BDBM544020	CC(C)c1nccc(C)c1-n1c2nc(N3CCCCC3C)c(Cl)cc2c(nc1=O)N1C[C@@H](C)N(C[C@@H]1C)C(=O)C=C	5.54
BDBM516169	Brc1ccc2n(CC(=O)NC3CN(C3)C(=O)C=C)c3cnccc3c2c1	5.54

BDBM544067	CC(C)c1nccc(C)c1-n1c2[nH]c(=O)c(Cl)cc2c(nc1=O)N1C[C@@H](C)N(C[C@@H]1C)C(=O)C=C	5.53
BDBM516189	Brc1ccc2n(CC(=O)NC3CN(C3)C(=O)C=C)cc(C(=O)N3CCO[C@H](C3)c3ccccc3)c2c1	5.53
BDBM516166	CC(C)(C)OC(=O)N1CCN(CC1)C(=O)c1cn(CC(=O)NC2CN(C2)C(=O)C=C)c2ccc(Br)cc12	5.52
BDBM516119	COc1ccccc1-c1cn(CC(=O)NC2CN(C2)C(=O)C=C)c2ccc(cc12)-c1ccccc1OC	5.52
BDBM516067	COc1ccc(Br)c2CN(CCc12)C(=O)c1cn(CC(=O)NC2CN(C2)C(=O)C=C)c2ccc(Br)cc12	5.52
BDBM516271	CC1C(CN1C(=O)C=C)NC(=O)Cn1ccc2cc(Br)ccc12	5.52
BDBM516126	Brc1ccc2n(CC(=O)NC3CN(C3)C(=O)C=C)cc(-c3cccc(c3)C#N)c2c1	5.52
BDBM535151	CC(=O)Nc1ccc(C)c(n1)-c1cc2ncnc(N3CCN(CC3)C(=O)C=C)c2cc1Cl	5.52
BDBM516192	CC(CNC(=O)c1cn(CC(=O)NC2CN(C2)C(=O)C=C)c2ccc(Br)cc12)c1ccccc1	5.51
BDBM543973	C[C@@H]1CN([C@@H](C)CN1C(=O)C=C)c1nc(=O)n(-c2ccccc2P(C)(C)=O)c2nc(c(Cl)cc12)-c1ccccc1F	5.5
BDBM516268	CN(C1CN(C1)C(=O)C=C)C(=O)Cn1ccc2cc(Cl)ccc12	5.49
BDBM516259	Cc1c(Br)ccc2n(CC(=O)NC3CN(C3)C(=O)C=C)ccc12	5.49
BDBM516158	CC(C)(C)OC(=O)N1CCc2c(C1)c1cc(Br)ccc1n2CC(=O)NC1CN(C1)C(=O)C=C	5.49
BDBM516212	Cc1c(C(=O)N2CCc3cnccc3C2)c2cc(Br)ccc2n1CC(=O)NC1CN(C1)C(=O)C=C	5.49
BDBM516175	Cc1cc(Br)cc2c(cn(CC(=O)NC3CN(C3)C(=O)C=C)c12)-c1cccc(c1)-c1ccccc1	5.49
BDBM515982	CCN(C1CN(C1)C(=O)C=C)C(=O)Cn1c(C)c(C(=O)N2CCc3ccccc3C2C)c2cc(Br)cc(C)c12	5.49
BDBM544113	CC(C)c1ncncc1-c1nc2n(-c3c(C)ccnc3C(C)C)c(=O)nc(N3C[C@@H](C)N(C[C@@H]3C)C(=O)C=C)c2cc1Cl	5.48
BDBM516172	Brc1ccc2n(CC(=O)NC3CN(C3)C(=O)C=C)c3c(CCCC3=O)c2c1	5.48
CHEMBL4476789	C=CC(=O)N1CCN(c2nnc(-c3ccccc3C)c3cc(-c4c(O)cccc4F)c(Cl)cc23)CC1	5.48
BDBM50539756	CN1CCC[C@H]1COc1nc2CN(CCc2c(n1)N1CCN(CC1CC#N)C(=O)C=C)c1cccc2ccccc12	5.48
BDBM50514384	Cc1ccccc1-c1nnc(N2CCN(CC2)C(=O)C=C)c2cc(Cl)c(cc12)-c1c(O)cccc1F	5.48
CHEMBL4639210	C=CC(=O)N1CCN(c2nc(OC[C@@H]3CCCN3C)nc3c2CCN(c2cccc4ccccc24)C3)C(CC#N)C1	5.48
BDBM516261	CN(C1CN(C1)C(=O)C=C)C(=O)Cn1ccc2cc(Br)cc(C)c12	5.48
BDBM544204	C[C@@H]1CN([C@@H](C)CN1C(=O)C=C)c1nc(=O)n(CC(C)(C)C#N)c2nc(c(Cl)cc12)-c1ccccc1F	5.47
BDBM516207	Brc1ccc2n(CC(=O)NC3CN(C3)C(=O)C=C)cc(C(=O)NCCc3ccco3)c2c1	5.47
BDBM544333	CC(C)c1nc(nc(C)c1-n1c2nc(c(Cl)cc2c(nc1=O)N1C[C@H](C)N(C[C@@H]1C)C(=O)C=C)-c1ccccc1F)C1CC1	5.46
BDBM516205	Brc1ccc2n(CC(=O)NC3CN(C3)C(=O)C=C)cc(C(=O)NCCc3cccnc3)c2c1	5.46
BDBM548332	CNC(=O)C[C@H](CC(C)C)Nc1nc(nc2ccccc12)N1CC(F)C2(CN(C2)C(=O)C=C)C1	5.46
CHEMBL4514914	C=CC(=O)N1CCN(c2nnc(-c3ccccc3Cl)c3cc(-c4c(O)cccc4F)c(Cl)cc23)CC1	5.45
BDBM516181	Brc1ccc2n(CC(=O)NC3CN(C3)C(=O)C=C)cc(C(=O)NCCn3ccccc3=O)c2c1	5.45
BDBM544438	CC(C)c1nccc(C)c1-n1c2nc(N3CCCCC3(C)C)c(Cl)cc2c(nc1=O)N1C[C@@H](C)N(C[C@@H]1C)C(=O)C=C	5.45
BDBM516018	COC(=O)c1cn(CC(=O)NC2CN(C2)C(=O)C=C)c2ccc(Br)cc12	5.45
BDBM50514371	Oc1cccc(F)c1-c1cc2c(nnc(N3CCN(CC3)C(=O)C=C)c2cc1Cl)-c1ccccc1Cl	5.45
BDBM544205	C[C@H]1CN(CCN1c1nc(=O)n(CC(C)(C)C#N)c2nc(c(Cl)cc12)-c1ccccc1F)C(=O)C=C	5.43
BDBM516098	Ic1ccc2n(CC(=O)NC3CN(C3)C(=O)C=C)ccc2c1	5.43
BDBM516217	Cc1c(C(=O)N2CCOCC2)c2cc(Br)ccc2n1CC(=O)NC1CN(C1)C(=O)C=C	5.42
BDBM544304	CC(C)(C)Cn1c2nc(c(Cl)cc2c(nc1=O)N1CCN(CC1CC#N)C(=O)C=C)-c1ccccc1F	5.42
BDBM544353	CC(C)c1nccc(C)c1-n1c2nc(c(F)cc2c(nc1=O)N1C[C@@H](C)N(C[C@@H]1C)C(=O)\C=C/CN(C)C)-c1ccccc1F	5.42
BDBM548315	CC(C)C[C@@H](CC1=NC(C)(C)C(=O)N1)Nc1nc(nc2CCCCc12)N1CCC2(CN(C2)C(=O)C=C)C1	5.41
BDBM535187	Clc1cc2c(ncnc2cc1-c1nc(NC(=O)C2CC2)cc2ccccc12)N1CCN(CC1)C(=O)C=C	5.4
BDBM516194	OC[C@@H](Cc1ccccc1)NC(=O)c1cn(CC(=O)NC2CN(C2)C(=O)C=C)c2ccc(Br)cc12	5.4

BDBM535114	Cc1cccnc1-c1cc2ncnc(N3CCN(CC3)C(=O)C=C)c2cc1Cl	5.39
BDBM516211	Cc1c(C(=O)N2CCc3ccncc3C2)c2cc(Br)ccc2n1CC(=O)NC1CN(C1)C(=O)C=C	5.39
BDBM516198	Brc1ccc2n(CC(=O)NC3CN(C3)C(=O)C=C)cc(C(=O)N[C@@H]3CCC[C@H]3c3ccccc3)c2c1	5.39
BDBM548322	CC(C)C[C@@H](Cc1nnc[nH]1)Nc1nc(nc2CCCCc12)N1CCC2(CN(C2)C(=O)C=C)C1	5.38
CHEMBL4569400	C=CC(=O)N1CC(NC(=O)Cn2cc(C(=O)NCCc3ccccc3)c3cc(Br)ccc32)C1	5.37
BDBM516140	Brc1ccc2n(CC(=O)NC3CN(C3)C(=O)C=C)cc(C(=O)N3CCN4CCCC[C@H]4C3)c2c1	5.37
CHEMBL4647242	C=CC(=O)N1CCN(c2nc(OC[C@@H]3CCCN3C)nc3c2CCN(c2cccc4ccccc24)C3)CC1	5.36
BDBM544311	C[C@@H]1CN([C@@H](C)CN1C(=O)C=C)c1nc(=O)n2-c3ccccc3C\C=C\Cc3ccccc3-c3nc2c1cc3Cl	5.36
BDBM516179	Brc1ccc2n(CC(=O)NC3CN(C3)C(=O)C=C)cc(C(=O)NCCn3cccn3)c2c1	5.36
CHEMBL4456612	C=CC(=O)N1CCN2c3c(cnc4cc(-c5c(C)ccc6[nH]ncc56)c(Cl)cc34)OCC[C@@H]2C1	5.36
BDBM516094	Brc1ccc2n(CC(=O)NC3CN(C3)C(=O)C=C)cc(C=O)c2c1	5.36
BDBM50539748	CN1CCC[C@H]1COc1nc2CN(CCc2c(n1)N1CCN(CC1)C(=O)C=C)c1cccc2ccccc12	5.36
BDBM50527062	Cc1ccc2[nH]ncc2c1-c1cc2ncc3OCC[C@@H]4CN(CCN4c3c2cc1Cl)C(=O)C=C	5.36
BDBM516222	CC(C)[C@@H]1CN(CCO1)C(=O)c1c(C)n(CC(=O)NC2CN(C2)C(=O)C=C)c2ccc(Br)cc12	5.35
BDBM516255	CC(C)COc1ncccc1-c1c(C)n(CC(=O)NC2CN(C2)C(=O)C=C)c2c(C)cc(Cl)cc12	5.35
BDBM516096	Cc1cc2n(CC(=O)NC3CN(C3)C(=O)C=C)ccc2cc1Br	5.35
BDBM516124	Brc1ccc2n(CC(=O)NC3CN(C3)C(=O)C=C)cc(CC#N)c2c1	5.35
BDBM548318	CNC(=O)C[C@H](CC(C)C)Nc1nc(nc2[C@@H](C)CCCc12)N1CCC2(CN(C2)C(=O)C=C)C1	5.35
BDBM50539750	CN1CCC[C@H]1COc1nc2CN(CCc2c(n1)N1CCN(CC1CO)C(=O)C=C)c1cccc2ccccc12	5.34
CHEMBL4649174	C=CC(=O)N1CCN(c2nc(OC[C@@H]3CCCN3C)nc3c2CCN(c2cccc4ccccc24)C3)C(CO)C1	5.34
BDBM544358	CC(C)c1ccccc1-c1c(CN2CCOCC2)nc(N2CCN(CC2)C(=O)C=C)c2cc(Cl)c(nc12)-c1ccccc1F	5.34
BDBM548328	CNC(=O)C[C@H](CC(C)C)Nc1nc(nc2CCCCc12)N1CC2(CN(C2)C(=O)C=C)C[C@@H]1CO	5.34
BDBM516138	Brc1ccc2n(CC(=O)NC3CN(C3)C(=O)C=C)cc(C(=O)N3CCC4(CCc5ccccc45)CC3)c2c1	5.33
CHEMBL4643600	C=CC(=O)N1CCN(c2nc(OC[C@@H]3CCCN3C)nc3c2CCN(c2cccc4ccccc24)C3)CC1CO	5.33
BDBM50539749	CN1CCC[C@H]1COc1nc2CN(CCc2c(n1)N1CCN(C(CO)C1)C(=O)C=C)c1cccc2ccccc12	5.33
BDBM516182	Brc1ccc2n(CC(=O)NC3CN(C3)C(=O)C=C)cc(C(=O)NCCN3CCNC3=O)c2c1	5.32
CHEMBL4638484	C=CC(=O)N1CCN(c2nc(OC[C@@H]3CCCN3C)nc3c2CCN(c2cccc4ccccc24)C3)C(CCO)C1	5.32
BDBM50539752	CN1CCC[C@H]1COc1nc2CN(CCc2c(n1)N1CCN(CC1CCO)C(=O)C=C)c1cccc2ccccc12	5.32
BDBM548361	CNC(=O)C[C@H](CC(C)C)Nc1nc(nc2cnccc12)N1CCC2(CN(C2)C(=O)C=C)C1	5.32
BDBM516164	CC(C)(C)OC(=O)N1Cc2c(C1)c1cc(Br)ccc1n2CC(=O)NC1CN(C1)C(=O)C=C	5.31
BDBM50517239	C[C@H]1CN(CCN1c1ncnc2CC(CCc12)c1c(C)ccc2[nH]ncc12)C(=O)C=C	5.31
BDBM516165	Fc1c(Br)ccc2n(CC(=O)NC3CN(C3)C(=O)C=C)ccc12	5.31
CHEMBL4441401	C=CC(=O)N1CCN(c2ncnc3c2CCC(c2c(C)ccc4[nH]ncc24)C3)[C@@H](C)C1	5.31
CHEMBL4649393	C=CC(=O)N1CCN(c2nc(OC[C@@H]3CCCN3C)nc3c2CCN(c2cccc4ccccc24)C3)CC1CCO	5.3
BDBM544196	COC(C)(C)Cn1c2nc(c(Cl)cc2c(nc1=O)N1C[C@@H](C)N(C[C@@H]1C)C(=O)C=C)-c1ccccc1F	5.3
BDBM50539764	COC(=C)C(=O)N1CCN(C[C@@H]1CC#N)c1nc(OC[C@@H]2CCCN2C)nc2CN(CCc12)c1cccc2cccc(Cl)c12	5.3
BDBM50539751	CN1CCC[C@H]1COc1nc2CN(CCc2c(n1)N1CCN(C(CCO)C1)C(=O)C=C)c1cccc2ccccc12	5.3
CHEMBL4635454	C=C(OC)C(=O)N1CCN(c2nc(OC[C@@H]3CCCN3C)nc3c2CCN(c2cccc4cccc(Cl)c24)C3)C[C@@H]1CC#N	5.3
BDBM535127	COc1ccc2c(nc(N)cc2c1)-c1cc2ncnc(N3CCN(CC3)C(=O)C=C)c2cc1Cl	5.29
BDBM516123	C=CC(=O)N1CC(C1)NC(=O)Cn1cc(Cc2ccccc2)c2ccccc12	5.29
BDBM544160	CC(C)c1ncnc(C(C)C)c1-n1c2nc(c(Cl)cc2c(nc1=O)N1C[C@@H](C)N(C[C@@H]1C)C(=O)C=C)-c1ccccc1S(C)(=O)=O	5.29

BDBM516121	Brc1ccc2n(CC(=O)NC3CN(C3)C(=O)C=C)cc(C#N)c2c1	5.28
BDBM516168	Clc1cc2ccn(CC(=O)NC3CN(C3)C(=O)C=C)c2cc1Cl	5.28
BDBM544345	CC(C)c1nc(cc(C)c1-n1c2nc(c(Cl)cc2c(nc1=O)N1C[C@H](C)N(C[C@@H]1C)C(=O)C=C)-c1ccccc1F)C(C)=C	5.28
BDBM516200	FC(F)(F)C(Cc1ccccc1)NC(=O)c1cn(CC(=O)NC2CN(C2)C(=O)C=C)c2ccc(Br)cc12	5.28
BDBM516139	Clc1ccccc1-c1ccc2c(cn(CC(=O)NC3CN(C3)C(=O)C=C)c2c1)-c1cc[nH]n1	5.27
BDBM516107	Cn1cc(cn1)-c1cn(CC(=O)NC2CN(C2)C(=O)C=C)c2ccc(Cl)cc12	5.26
BDBM544027	CC(C)c1nccc(C)c1-n1c2nc(c(Cl)cc2c(nc1=O)N1[C@H]2CCC[C@@H]1CN(C2)C(=O)C=C)-c1ccccc1F	5.26
BDBM50517190	Brc1ccc2n(CC(=O)NC3CN(C3)C(=O)C=C)ccc2c1	5.26
BDBM516145	FC(F)(F)c1ccc2n(CC(=O)NC3CN(C3)C(=O)C=C)ccc2c1	5.26
BDBM516160	Brc1ccc2n(CC(=O)NC3CN(C3)C(=O)C=C)c3CNCCc3c2c1	5.24
CHEMBL2028663	CC(C)(C)c1nc(-c2cccc(NS(=O)(=O)c3c(F)cccc3F)c2F)c(-c2ccnc(N)n2)s1	5.23
BDBM548316	CNC(=O)C[C@H](CC(C)C)Nc1nc(nc2COCc12)N1CCC2(CN(C2)C(=O)C=C)C1	5.23
BDBM516114	CCOC(=O)C(=O)c1cn(CC(=O)NC2CN(C2)C(=O)C=C)c2ccc(Br)cc12	5.23
BDBM516184	Brc1ccc2n(CC(=O)NC3CN(C3)C(=O)C=C)cc(C(=O)NCCc3ccccn3)c2c1	5.23
BDBM516106	Clc1ccc2n(CC(=O)NC3CN(C3)C(=O)C=C)c(cc2c1)-c1ncccn1	5.22
BDBM516196	NC(=O)[C@@H](Cc1ccccc1)NC(=O)c1cn(CC(=O)NC2CN(C2)C(=O)C=C)c2ccc(Br)cc12	5.22
BDBM515942	CN(C)Cc1ccccc1-c1c(C)n(CC(=O)NC2CN(C2)C(=O)C=C)c2ccc(Cl)cc12	5.22
BDBM544197	COC(C)(C)Cn1c2nc(c(Cl)cc2c(nc1=O)N1CCN(CC1)C(=O)C=C)-c1ccccc1F	5.21
BDBM516225	Cc1c(CN2CCN(CC2)c2ccc(cn2)C#N)c2cc(Br)ccc2n1CC(=O)NC1CN(C1)C(=O)C=C	5.21
BDBM516230	CC1N(CCc2ccccc12)C(=O)c1c(C)n(CC(=O)NC2CN(C2)C(=O)C=C)c2c(C)cc(cc12)C#N	5.19
BDBM516132	Brc1ccc2n(CC(=O)NC3CN(C3)C(=O)C=C)cc(C(=O)N3CCN4CCOC[C@@H]4C3)c2c1	5.19
BDBM516131	CC(C)(C)OC(=O)c1cn(CC(=O)NC2CN(C2)C(=O)C=C)c2ccc(Br)cc12	5.19
BDBM516091	COC(=O)c1cc2cc(Br)ccc2n1CC(=O)NC1CN(C1)C(=O)C=C	5.19
BDBM516015	CC(C)Oc1ncccc1-c1cn(CC(=O)NC2CN(C2)C(=O)C=C)c2ccc(Br)cc12	5.19
CHEMBL4522790	C=CC(=O)N1CC(NC(=O)Cn2cc(C(=O)N(C)Cc3ccccc3)c3cc(Br)ccc32)C1	5.18
BDBM516185	Brc1ccc2n(CC(=O)NC3CN(C3)C(=O)C=C)cc(C(=O)NCCc3ncc[nH]3)c2c1	5.18
BDBM516206	Brc1ccc2n(CC(=O)NC3CN(C3)C(=O)C=C)cc(C(=O)NCCN3CCSCC3)c2c1	5.18
BDBM516267	CN(C1CN(C1)C(=O)C=C)C(=O)Cn1ccc2cc(Br)c(C)cc12	5.17
BDBM516122	Ic1cn(CC(=O)NC2CN(C2)C(=O)C=C)c2ccccc12	5.17
BDBM535124	Nc1cc2cccnc2c(n1)-c1cc2ncnc(N3CCN(CC3)C(=O)C=C)c2cc1Cl	5.16
CHEMBL598038	COc1cc(/C=C/C(=O)c2ccc([N+](=O)[O-])cc2)cc(OC)c1OC	5.15
BDBM50542430	COc1cc(\C=C\C(=O)c2ccc(cc2)[N+]([O-])=O)cc(OC)c1OC	5.15
BDBM516097	Clc1ccc2n(CC(=O)NC3CN(C3)C(=O)C=C)ccc2c1	5.14
BDBM50542434	COc1cc(cc(OC)c1OC)C(=O)\C=C\c1c(F)cccc1F	5.13
BDBM50542432	COc1cc(\C=C\C(=O)c2ccc(cc2)N(C)C)cc(OC)c1OC	5.13
CHEMBL4646677	COc1cc(C(=O)/C=C/c2c(F)cccc2F)cc(OC)c1OC	5.13
CHEMBL62070	COc1cc(/C=C/C(=O)c2ccc(N(C)C)cc2)cc(OC)c1OC	5.13
BDBM516220	COc1cccc2CN(CCc12)C(=O)c1c(C)n([C@H]2CCN(C3CN(C3)C(=O)C=C)C2=O)c2ccc(Br)cc12	5.13
BDBM516202	CN(C)C(=O)C(Cc1ccccc1)NC(=O)c1cn(CC(=O)NC2CN(C2)C(=O)C=C)c2ccc(Br)cc12	5.13
BDBM50527060	C[C@H]1CN2[C@@H](COc3cnc4cc(c(Cl)cc4c23)-c2c(O)cccc2F)CN1C(=O)C=C	5.12
BDBM516152	CC(=O)n1c2CN(CC(=O)NC3CN(C3)C(=O)C=C)Cc2c2cc(Br)ccc12	5.12

BDBM516269	Brc1ccc2n(Cc3cnnn3C3CN(C3)C(=O)C=C)ccc2c1	5.12
CHEMBL4461958	C=CC(=O)N1C[C@@H]2COc3cnc4cc(-c5c(O)cccc5F)c(Cl)cc4c3N2C[C@@H]1C	5.12
BDBM50507446	Oc1cc(N2CCc3c(C2)ncnc3N2CCN(CC2)C(=O)C=C)c2ccccc2c1	5.12
CHEMBL4457300	C=CC(=O)N1CCN(c2ncnc3c2CCN(c2cc(O)cc4ccccc24)C3)CC1	5.12
BDBM516226	COCC1CCN(CC1)C(=O)c1c(C2CC2)n(CC(=O)NC2CN(C2)C(=O)C=C)c2c(C)cc(Cl)cc12	5.11
BDBM544017	CC(C)n1c2nc(c(Cl)cc2c(nc1=O)N1C[C@@H](C)N(C[C@@H]1C)C(=O)C=C)-c1ccccc1F	5.11
BDBM516186	Brc1ccc2n(CC(=O)NC3CN(C3)C(=O)C=C)cc(C(=O)NCCn3ccnc3)c2c1	5.11
BDBM516161	CC(=O)N1CCc2c(C1)c1cc(Br)ccc1n2CC(=O)NC1CN(C1)C(=O)C=C	5.1
BDBM544314	C[C@@H]1CN([C@@H](C)CN1C(=O)C=C)c1nc(=O)n2-c3ccccc3C\C=C/Cc3ccccc3-c3nc2c1cc3Cl	5.1
CHEMBL59351	COc1cc(C(=O)/C=C/c2ccc([N+](=O)[O-])cc2)cc(OC)c1OC	5.09
CHEMBL4643559	COc1cc(/C=C/C(=O)c2c(F)cccc2F)cc(OC)c1OC	5.09
BDBM50514385	COc1ccccc1-c1nnc(N2CCN(CC2)C(=O)C=C)c2cc(Cl)c(cc12)-c1c(O)cccc1F	5.09
BDBM516274	C[C@H]1[C@H](CN1C(=O)C=C)N(C)C(=O)Cn1ccc2cc(Br)ccc12	5.09
BDBM544316	C[C@@H]1CN([C@@H](C)CN1C(=O)C=C)c1nc(=O)n2-c3ccccc3C\C=C\COc3ccccc3-c3nc2c1cc3Cl	5.09
BDBM516204	CN1CCN(CCNC(=O)c2cn(CC(=O)NC3CN(C3)C(=O)C=C)c3ccc(Br)cc23)CC1	5.09
CHEMBL4466063	C=CC(=O)N1CCN(c2nnc(-c3ccccc3OC)c3cc(-c4c(O)cccc4F)c(Cl)cc23)CC1	5.09
BDBM50542429	COc1cc(\C=C\C(=O)c2c(F)cccc2F)cc(OC)c1OC	5.09
BDBM50542433	COc1cc(cc(OC)c1OC)C(=O)\C=C\c1ccc(cc1)[N+]([O-])=O	5.09
BDBM544195	CC(C)(C)Cn1c2nc(c(Cl)cc2c(nc1=O)N1CCN([C@@H](CO)C1)C(=O)C=C)-c1ccccc1F	5.08
CHEMBL187437	COc1ccc(C(=O)/C=C/c2cc(OC)c(OC)c(OC)c2)cc1	5.08
BDBM50542431	COc1ccc(cc1)C(=O)\C=C\c1cc(OC)c(OC)c(OC)c1	5.08
BDBM544054	CC(C)c1nccc(C)c1-n1c2nc(C3=CCOCC3)c(Cl)cc2c(nc1=O)N1C[C@@H](C)N(C[C@@H]1C)C(=O)C=C	5.07
BDBM516183	Brc1ccc2n(CC(=O)NC3CN(C3)C(=O)C=C)cc(C(=O)NCCc3c[nH]cn3)c2c1	5.07
BDBM548320	CNC(=O)C[C@H](CC(C)C)Nc1nc(nc2n(C)cnc12)N1CCC2(CN(C2)C(=O)C=C)C1	5.06
BDBM544117	CC(C)c1nccc(C)c1-n1c2nc(-c3scnc3C)c(Cl)cc2c(nc1=O)N1C[C@@H](C)N(C[C@@H]1C)C(=O)C=C	5.05
CHEMBL471655	COc1cc(/C=C/C(=O)c2ccc(N)cc2)cc(OC)c1OC	5.04
BDBM50542426	COc1cc(\C=C\C(=O)c2ccc(N)cc2)cc(OC)c1OC	5.04
BDBM544295	CC(C)c1nccc(C)c1-n1c2nc(Cl)c(Cl)cc2c(nc1=O)N1CCN(C(CC#N)C1)C(=O)C=C	5.04
BDBM548324	CNC(=O)C[C@H](Nc1nc(nc2CCCCc12)N1CCC2(CN(C2)C(=O)C=C)C1)c1cccc(c1)C#N	5.04
BDBM535189	CC(C)Nc1cc2cccc(-c3cc4ncnc(N5CCN(CC5)C(=O)C=C)c4cc3Cl)c2cn1	5.04
CHEMBL4746420	C=CC(=O)N1CCN(c2nc(=O)n(-c3c(C(C)C)ncnc3C(C)C)c3c(F)c(-c4c(O)cccc4F)ncc23)[C@@H](C)C1	5
CHEMBL4781219	C=CC(=O)N1CCN(c2nc(=O)n(-c3c(C(C)C)cccc3C(C)C)c3c(F)c(-c4c(O)cccc4F)ncc23)[C@@H](C)C1	5
BDBM50562644	CC(C)c1ncnc(C(C)C)c1-n1c2c(F)c(ncc2c(nc1=O)N1CCN(C[C@@H]1C)C(=O)C=C)-c1c(O)cccc1F	5
CHEMBL4754887	C=CC(=O)N1CCN(c2nc(=O)n(-c3c(C)ccnc3C(C)C)c3c(F)c(-c4c(O)cccc4F)ncc23)[C@@H](C)C1	5
BDBM50562642	CC(C)c1nccc(C)c1-n1c2c(Cl)c(ncc2c(nc1=O)N1CCN(C[C@@H]1C)C(=O)C=C)-c1c(O)cccc1F	5
CHEMBL4784376	C=CC(=O)N1CCN(c2nc(=O)n(-c3c(C)ccnc3C(C)C)c3c(Cl)c(-c4c(O)cccc4F)ncc23)[C@@H](C)C1	5
BDBM50562641	CC(C)c1nccc(C)c1-n1c2c(F)c(ncc2c(nc1=O)N1CCN(C[C@@H]1C)C(=O)C=C)-c1c(O)cccc1F	5
BDBM50562645	CC(C)c1cccc(C(C)C)c1-n1c2c(F)c(ncc2c(nc1=O)N1CCN(C[C@@H]1C)C(=O)C=C)-c1c(O)cccc1F	5
BDBM50562643	CC(C)c1ncnc(C(C)C)c1-n1c2c(Cl)c(ncc2c(nc1=O)N1CCN(C[C@@H]1C)C(=O)C=C)-c1c(O)cccc1F	5
CHEMBL4741576	C=CC(=O)N1CCN(c2nc(=O)n(-c3c(C(C)C)ncnc3C(C)C)c3c(Cl)c(-c4c(O)cccc4F)ncc23)[C@@H](C)C1	5
BDBM516148	Brc1ccc2n(CC(=O)NC3CN(C3)C(=O)C=C)c3CCN=Cc3c2c1	5

BDBM543965	COc1nc2n(-c3c(Cl)ccnc3C(C)C)c(=O)nc(N3C[C@@H](C)N(C[C@@H]3C)C(=O)C=C)c2cc1-c1ccccc1F	4.99
BDBM544018	CC(C)c1nccc(C)c1-n1c2nc(N3CCCC3C(F)F)c(Cl)cc2c(nc1=O)N1C[C@@H](C)N(C[C@@H]1C)C(=O)C=C	4.96
BDBM516120	Fc1ccc(-c2cn(CC(=O)NC3CN(C3)C(=O)C=C)c3ccc(cc23)-c2ccc(F)cc2F)c(F)c1	4.96
BDBM516115	Cc1ccc2cn[nH]c2c1-c1cn(CC(=O)NC2CN(C2)C(=O)C=C)c2ccc(cc12)-c1c(C)ccc2cn[nH]c12	4.94
BDBM516224	Cc1c(-c2cnccc2C#N)c2cc(Cl)ccc2n1CC(=O)NC1CN(C1)C(=O)C=C	4.93
BDBM544206	CC(C)(Cn1c2nc(c(Cl)cc2c(nc1=O)N1CCN(CC1)C(=O)C=C)-c1ccccc1F)C#N	4.92
BDBM516118	Fc1ccc2cn[nH]c2c1-c1cn(CC(=O)NC2CN(C2)C(=O)C=C)c2ccc(cc12)-c1c(F)ccc2cn[nH]c12	4.89
BDBM544225	C[C@H]1CN([C@@H](C)CN1C(=O)C=C)c1nc(=O)n(CC(C)(C)C)c2nc(c(Cl)cc12)-c1ccccc1F	4.87
BDBM516253	COc1cccc(OC)c1-c1c(C)n(CC(=O)NC2CN(C2)C(=O)C=C)c2c(C)cc(Cl)cc12	4.87
CHEMBL4455193	C=CC(=O)N1CC(NC(=O)Cn2cc(C(=O)NCc3ccccc3)c3cc(Br)ccc32)C1	4.85
BDBM516203	Brc1ccc2n(CC(=O)NC3CN(C3)C(=O)C=C)cc(C(=O)NCCNC(=O)C3CC3)c2c1	4.85
BDBM516227	Clc1ccc2n(CC(=O)NC3CN(C3)C(=O)C=C)ccc2c1Cl	4.81
CHEMBL4540074	C=CC(=O)N1CCN(c2ncnc3c2CCN(c2cccc4ccc(O)cc24)C3)CC1	4.8
BDBM516095	Brc1ccc2n(CC(=O)NC3CN(C3)C(=O)C=C)c(C=O)cc2c1	4.8
CHEMBL4549643	C=CC(=O)N1CCN(c2ncnc3c2CCN(c2c(C)ccc4[nH]ncc24)C3)CC1	4.8
CHEMBL59026	COc1cc(/C=C/C(=O)c2ccc(F)cc2)cc(OC)c1OC	4.8
BDBM50507453	C=CC(=O)N1CCN(CC1)c1ncnc2CN(CCc12)c1cccc2ccccc12	4.8
BDBM50115777	COc1cc(\C=C\C(=O)c2ccc(F)cc2)cc(OC)c1OC	4.8
BDBM50507449	Oc1cccc(F)c1N1CCc2c(C1)ncnc2N1CCN(CC1)C(=O)C=C	4.8
BDBM50507454	Oc1ccc(F)c(c1)N1CCc2c(C1)ncnc2N1CCN(CC1)C(=O)C=C	4.8
BDBM50507448	Cc1ccc2[nH]ncc2c1N1CCc2c(C1)ncnc2N1CCN(CC1)C(=O)C=C	4.8
BDBM50507450	Oc1ccc2cccc(N3CCc4c(C3)ncnc4N3CCN(CC3)C(=O)C=C)c2c1	4.8
CHEMBL4453012	C=CC(=O)N1CCN(c2ncnc3c2CCN(c2cc(O)ccc2F)C3)CC1	4.8
CHEMBL4466927	C=CC(=O)N1CCN(c2ncnc3c2CCN(c2cccc4ccccc24)C3)CC1	4.8
CHEMBL4453071	C=CC(=O)N1CCN(c2ncnc3c2CCN(c2c(O)cccc2F)C3)CC1	4.8
BDBM516173	Cc1cc(cc2c(cn(CC(=O)NC3CN(C3)C(=O)C=C)c12)-c1ccc2ccccc2c1)-c1ccc2ccccc2c1	4.79
BDBM516228	Cc1c(-c2ccccc2C(C)(C)C)c2cc(Cl)ccc2n1CC(=O)NC1CN(C1)C(=O)C=C	4.79
CHEMBL1229517	CCCS(=O)(=O)Nc1ccc(F)c(C(=O)c2c[nH]c3ncc(-c4ccc(Cl)cc4)cc23)c1F	4.78
BDBM516117	C=CC(=O)N1CC(C1)NC(=O)Cn1cc(-c2cccnc2)c2cc(ccc12)-c1cccnc1	4.74
BDBM535188	OCC(=O)Nc1cc2ccccc2c(n1)-c1cc2ncnc(N3CCN(CC3)C(=O)C=C)c2cc1Cl	4.72
CHEMBL4460786	C=CC(=O)N1CCN(c2nnc(-c3ccccc3)c3cc(-c4c(O)cccc4F)c(Cl)cc23)CC1	4.7
BDBM516135	OC(=O)c1ccc(CNC(=O)c2cc3cc(Br)ccc3n2CC(=O)NC2CN(C2)C(=O)C=C)s1	4.7
BDBM50514373	Oc1cccc(F)c1-c1cc2c(nnc(N3CCN(CC3)C(=O)C=C)c2cc1Cl)-c1ccccc1	4.7
BDBM516109	Clc1cccc2n(CC(=O)NC3CN(C3)C(=O)C=C)ccc12	4.67
BDBM516159	Brc1ccc2n(CC(=O)NC3CN(C3)C(=O)C=C)c3CCNCc3c2c1	4.66
BDBM516241	CN1Cc2ccccc2CC1CNC(=O)Cc1cn(CC(=O)NC2CN(C2)C(=O)C=C)c2ccc(Br)cc12	4.65
BDBM544226	CC#CC(=O)N1C[C@H](C)N(C[C@H]1C)c1nc(=O)n(-c2c(C)ccnc2C(C)C)c2nc(c(F)cc12)-c1ccccc1F	4.62
CHEMBL589993	COc1cc(/C=C/C(=O)c2ccc(Br)cc2)cc(OC)c1OC	4.61
BDBM50542427	COc1cc(\C=C\C(=O)c2ccc(Br)cc2)cc(OC)c1OC	4.61
BDBM516262	CC1(CN(C1)C(=O)C=C)NC(=O)Cn1ccc2cc(Br)ccc12	4.6
BDBM516170	Brc1ccc2n(CC(=O)NC3CN(C3)C(=O)C=C)cc(C(=O)N3CCNCC3)c2c1	4.58

BDBM516236	Brc1ccc2n(CC(=O)NC3CN(C3)C(=O)C=C)cc(CC(=O)N3CCCCC3CN3CCCCC3)c2c1	4.58
BDBM544026	CC(C)c1nccc(C)c1-n1c2nc(c(Cl)cc2c(nc1=O)N1C[C@@H]2CC[C@H]1CN2C(=O)C=C)-c1ccccc1F	4.57
BDBM516238	Brc1ccc2n(CC(=O)NC3CN(C3)C(=O)C=C)cc(CC(=O)N3CCC4(C3)CCOCC4)c2c1	4.51
BDBM516266	CN(C(=O)Cn1ccc2cc(Br)ccc12)C1(C)CN(C1)C(=O)C=C	4.5
BDBM544140	COc1cc(-c2nc3n(-c4c(C)ccnc4C(C)C)c(=O)nc(N4C[C@@H](C)N(C[C@@H]4C)C(=O)C=C)c3cc2F)c2ccccc2c1	4.49
BDBM50549223	CN(C)Cc1[nH]c2ccccc2c1[C@H]1NC(=O)c2ccc(O)cc12	4.48
CHEMBL4796065	CN(C)Cc1[nH]c2ccccc2c1[C@H]1NC(=O)c2ccc(O)cc21	4.48
BDBM516105	C=CC(=O)N1CC(C1)NC(=O)Cn1c2ccccc2c2ccccc12	4.47
BDBM544147	CC(C)c1nccc(C)c1-n1c2nc(C#CC(C)(C)C)c(Cl)cc2c(nc1=O)N1C[C@@H](C)N(C[C@@H]1C)C(=O)C=C	4.47
BDBM516229	Cc1cc(Cl)cc2cc(C3CC3)n(C3CCN(C(=O)NC4CN(C4)C(=O)C=C)C3=O)c12	4.46
BDBM516240	OC(=O)c1ccc2CCN(Cc2c1)C(=O)Cc1cn(CC(=O)NC2CN(C2)C(=O)C=C)c2ccc(Br)cc12	4.44
BDBM516231	Cc1c(C(=O)N2CCN(CC2)c2ccc(cn2)C#N)c2cc(Br)ccc2n1[C@@H]1CCN(C1)C1CN(C1)C(=O)C=C	4.44
BDBM544133	CC(C)c1nccc(C)c1-n1c2nc(c(F)cc2c(nc1=O)N1C[C@@H](C)N(C[C@@H]1C)C(=O)C=C)-c1cccc2[nH]cnc12	4.42
BDBM516149	Brc1ccc2n(CC(=O)NC3CN(C3)C(=O)C=C)c3CN(Cc4ccccc4)CCc3c2c1	4.36
BDBM516242	Brc1ccc2n(CC(=O)NC3CN(C3)C(=O)C=C)cc(CC(=O)N3CCc4cn[nH]c4C3)c2c1	4.36
BDBM516237	Brc1ccc2n(CC(=O)NC3CN(C3)C(=O)C=C)cc(CC(=O)N3CCC(CC3)c3ncc[nH]3)c2c1	4.34
CHEMBL3219765	COc1cc(/C=C/C(=O)c2ccccn2)cc(OC)c1OC	4.28
BDBM50542428	COc1cc(\C=C\C(=O)c2ccccn2)cc(OC)c1OC	4.28
BDBM548329	CNC(=O)C[C@H](CC(C)C)Nc1nc(nc2CCCCc12)N1CC2(CN(C2)C(=O)C=C)C[C@H]1CO	4.23
BDBM544158	CC(C)(C)Cn1c2nc(Cl)c(Cl)cc2c(nc1=O)N1CCN(CC1)C(=O)C=C	4.23
BDBM516102	Brc1ccc2n(CC(=O)NC3CN(C3)C(=O)C=C)c(C(=O)NCc3ccccc3)c(I)c2c1	4.21
BDBM516264	CN(C(=O)Cn1ccc2cc(Br)ccc12)C1(CO)CN(C1)C(=O)C=C	4.2
BDBM516176	CSc1ccc2n(CC(=O)NC3CN(C3)C(=O)C=C)ccc2c1	4.2
BDBM516190	Clc1ccc2c3ccccc3n(CC(=O)NC3CN(C3)C(=O)C=C)c2n1	4.2
BDBM544135	CC(C)c1nccc(C)c1-n1c2nc(ccc2c(nc1=O)N1C[C@@H](C)N(C[C@@H]1C)C(=O)C=C)-c1ccccc1C(O)=O	4.13
BDBM516243	Brc1ccc2n(CC(=O)NC3CN(C3)C(=O)C=C)cc(CC(=O)N3CC4CCC3C4)c2c1	4.1
BDBM516239	CN(C)C1CCC2C(C1)OCCN2C(=O)Cc1cn(CC(=O)NC2CN(C2)C(=O)C=C)c2ccc(Br)cc12	4.07
BDBM516263	OCC1(CN(C1)C(=O)C=C)NC(=O)Cn1ccc2cc(Br)ccc12	4.03
BDBM516233	COc1cccc2CN(CCc12)C(=O)c1c(C2CC2)n([C@@H]2CCN(C2)C2CN(C2)C(=O)C=C)c2c(C)cc(Cl)cc12	4.03
BDBM516142	[O-][N+](=O)c1ccc2n(CC(=O)NC3CN(C3)C(=O)C=C)ccc2c1	4.03
BDBM516188	Cc1c(C(=O)OC(C)(C)C)c2cc(ccc2n1CC(=O)NC1CN(C1)C(=O)C=C)[N+]([O-])=O	3.98
BDBM516197	Brc1ccc2n(CC(=O)NC3CN(C3)C(=O)C=C)cc(C(=O)N[C@@H]3CCCC[C@H]3c3ccccc3)c2c1	3.92
BDBM516127	C=CC(=O)N1CC(C1)NC(=O)Cn1ccc2cc(ccc12)C1CC1	3.9
BDBM548325	CNC(=O)C[C@@H](Nc1nc(nc2CCCCc12)N1CCC2(CN(C2)C(=O)C=C)C1)c1cccc(c1)C#N	3.89
BDBM544319	CC(C)c1ccccc1-n1c2CN(CCc2c(nc1=O)N1C[C@@H](C)N(C[C@@H]1C)C(=O)C=C)c1ccccc1	3.88
BDBM535198	CS(=O)(=O)c1ccc(N)nc1-c1cc2ncnc(N3CCN(CC3)C(=O)C=C)c2cc1Cl	3.82
CHEMBL2396992	Cc1[nH]c2cc(Cl)cc(Cl)c2c1CCN	3.81
BDBM544007	C[C@@H]1CN([C@@H](C)CN1C(=O)C=C)c1nc(=O)n(C)c2nc(c(Cl)cc12)-c1ccccc1F	3.76
BDBM544179	CC(C)c1ccnc(C(=O)N(C)C)c1-n1c2nc(c(Cl)cc2c(nc1=O)N1C[C@@H](C)N(C[C@@H]1C)C(=O)C=C)-c1ccccc1F	3.74
BDBM548317	CNC(=O)C[C@H](CC(C)C)Nc1nc(nc2ncn(C)c12)N1CCC2(CN(C2)C(=O)C=C)C1	3.71
BDBM516128	Clc1ccc2c(n1)n(CC(=O)NC1CN(C1)C(=O)C=C)c1ccc(Br)cc21	3.6

BDBM516146	COc1ccc2n(CC(=O)NC3CN(C3)C(=O)C=C)ccc2c1	3.6
BDBM516125	C=CC(=O)N1CC(C1)NC(=O)Cn1c(cc2ccccc12)-c1ccncc1	3.6
BDBM516147	C=CC(=O)N1CC(C1)NC(=O)Cn1ccc2cc(ccc12)-c1ccccc1	3.6
CHEMBL4525691	C=CC(=O)N1CC(NC(=O)Cn2ccc3cc(Br)ccc32)C1	3.6
BDBM516093	C=CC(=O)N1CC(C1)NC(=O)Cn1ccc2ccccc12	3.6
BDBM516092	Brc1ccc2ccn(CC(=O)NC3CN(C3)C(=O)C=C)c2c1	3.6
BDBM516223	COc1cccc2CN(CCc12)C(=O)c1c(C)n(CC(=O)NC2CN(C2)C(=O)C(=C)CCO)c2ccc(Br)cc12	3.6
BDBM516110	CCOC(=O)C(=O)c1cn(CC(=O)NC2CN(C2)C(=O)C=C)c2ccccc12	3.6
BDBM516113	Clc1cccc2ccn(CC(=O)NC3CN(C3)C(=O)C=C)c12	3.6
BDBM516112	Clc1ccc2ccn(CC(=O)NC3CN(C3)C(=O)C=C)c2c1	3.6
BDBM516111	OC(=O)C(=O)c1cn(CC(=O)NC2CN(C2)C(=O)C=C)c2ccccc12	3.6
BDBM516108	Fc1ccc2n(CC(=O)NC3CN(C3)C(=O)C=C)ccc2c1	3.6
BDBM516101	C=CC(=O)N1CC(C1)NC(=O)Cn1ccc2cc(ccc12)C#N	3.6
BDBM516187	CCc1ccc2n(CC(=O)NC3CN(C3)C(=O)C=C)ccc2c1	3.6
BDBM544425	C[C@@H]1CN([C@@H](C)CN1C(=O)C=C)C1=NCC(=O)N(CC(C)(C)C)c2nc(c(Cl)cc12)-c1ccccc1F	3.6
BDBM544424	CC(C)c1nccc(C)c1N1c2nc(c(Cl)cc2C(=NCC1=O)N1C[C@@H](C)N(C[C@@H]1C)C(=O)C=C)-c1ccccc1F	3.6
BDBM544159	CC(C)(C)Cn1c2ncccc2c(nc1=O)N1CCN(CC1)C(=O)C=C	3.6
BDBM516272	C[C@@H]1[C@@H](CN1C(=O)C=C)NC(=O)Cn1ccc2cc(Br)ccc12	3.6
BDBM516270	C=CC(=O)N1CC(C1)NC(=O)Cn1ccc2cccc(CC#N)c12	3.6

Supplementary Table S3: Covalent FDA Drugs

Name	Smiles
Afatinib	CN(C)C/C=C/C(=O)Nc1cc2c(Nc3ccc(F)c(Cl)c3)ncnc2cc1O[C@H]1CCOC1
Abiraterone	C[C@]12CC[C@H]3[C@@H](CC=C4C[C@@H](O)CC[C@@]43C)[C@@H]1CC=C2c1cccnc1
Acalabrutinib	CC#CC(=O)N1CCC[C@H]1c1nc(-c2ccc(C(=O)Nc3ccccn3)cc2)c2c(N)nccn12
Acetylsalicylic acid	CC(=O)Oc1ccccc1C(=O)O
Amoxicillin	CC1(C)S[C@@H]2[C@H](NC(=O)[C@H](N)c3ccc(O)cc3)C(=O)N2[C@H]1C(=O)O
Avibactam	NC(=O)[C@@H]1CC[C@@H]2CN1C(=O)N2OS(=O)(=O)O
Azacitidine	Nc1ncn([C@@H]2O[C@H](CO)[C@@H](O)[C@H]2O)c(=O)n1
Boceprevir	CC(C)(C)NC(=O)N[C@H](C(=O)N1C[C@H]2[C@@H]([C@H]1C(=O)NC(CC1CCC1)C(=O)C(N)=O)C2(C)C)C(C)(C)C
Bortezomib	CC(C)C[C@H](NC(=O)[C@H](Cc1ccccc1)NC(=O)c1cnccn1)B(O)O
Carbidopa	C[C@@](Cc1ccc(O)c(O)c1)(NN)C(=O)O
Carfilzomib	CC(C)C[C@H](NC(=O)[C@H](CCc1ccccc1)NC(=O)CN1CCOCC1)C(=O)N[C@@H](Cc1ccccc1)C(=O)N[C@@H](CC(C)C)C(=O)[C@@]1(C)CO1
Cefaclor	N[C@@H](C(=O)N[C@@H]1C(=O)N2C(C(=O)O)=C(Cl)CS[C@H]12)c1ccccc1
Cefdinir	C=CC1=C(C(=O)O)N2C(=O)[C@@H](NC(=O)/C(=N\O)c3csc(N)n3)[C@H]2SC1
Cefprozil	CC=CC1=C(C(=O)O)N2C(=O)[C@@H](NC(=O)[C@H](N)c3ccc(O)cc3)[C@H]2SC1
Ceftriaxone	CO/N=C(\C(=O)N[C@@H]1C(=O)N2C(C(=O)O)=C(CSc3nc(=O)c(O)nn3C)CS[C@H]12)c1csc(N)n1
Cefuroxime	CO/N=C(\C(=O)N[C@@H]1C(=O)N2C(C(=O)O)=C(COC(N)=O)CS[C@H]12)c1ccco1
Cephalexin	CC1=C(C(=O)O)N2C(=O)[C@@H](NC(=O)[C@H](N)c3ccccc3)[C@H]2SC1
Clopidogrel	COC(=O)[C@H](c1ccccc1Cl)N1CCc2sccc2C1

Dacomitinib	COc1cc2ncnc(Nc3ccc(F)c(Cl)c3)c2cc1NC(=O)/C=C/CN1CCCCC1
Decitabine	Nc1ncn([C@H]2C[C@H](O)[C@@H](CO)O2)c(=O)n1
Dexlansoprazole	Cc1c(OCC(F)(F)F)ccnc1C[S@@+]([O-])c1nc2ccccc2[nH]1
Dimethyl fumarate	COC(=O)/C=C/C(=O)OC
Disulfiram	CCN(CC)C(=S)SSC(=S)N(CC)CC
Dutasteride	C[C@]12CC[C@H]3[C@@H](CC[C@H]4NC(=O)C=C[C@]34C)[C@@H]1CC[C@@H]2C(=O)Nc1cc(C(F)(F)F)ccc1C(F)(F)F
Echothiophate	CCOP(=O)(OCC)SCC[N+](C)(C)C
Eflornithine	NCCCC(N)(C(=O)O)C(F)F
Esomeprazole	COc1ccc2[nH]c([S@@+]([O-])Cc3ncc(C)c(OC)c3C)nc2c1
Exemestane	C=C1C[C@@H]2[C@H](CC[C@]3(C)C(=O)CC[C@@H]23)[C@@]2(C)C=CC(=O)C=C12
Finasteride	CC(C)(C)NC(=O)[C@H]1CC[C@H]2[C@@H]3CC[C@H]4NC(=O)C=C[C@]4(C)[C@H]3CC[C@]12C
Floxuridine	O=c1[nH]c(=O)n([C@H]2C[C@H](O)[C@@H](CO)O2)cc1F
Fosfomycin	C[C@@H]1O[C@@H]1P(=O)(O)O
Gemcitabine	Nc1ccn([C@@H]2O[C@H](CO)[C@@H](O)C2(F)F)c(=O)n1
Ibrutinib	C=CC(=O)N1CCC[C@@H](n2nc(-c3ccc(Oc4ccccc4)cc3)c3c(N)ncnc32)C1
Isoniazid	NNC(=O)c1ccncc1
Lansoprazole	Cc1c(OCC(F)(F)F)ccnc1C[S+]([O-])c1nc2ccccc2[nH]1
Malathion	CCOC(=O)CC(SP(=S)(OC)OC)C(=O)OCC
Mercaptopurine	Sc1ncnc2nc[nH]c12
Meropenem	C[C@@H](O)[C@H]1C(=O)N2C(C(=O)O)=C(S[C@@H]3CN[C@H](C(=O)N(C)C)C3)[C@H](C)[C@H]12
Nafcillin	CCOc1ccc2ccccc2c1C(=O)N[C@@H]1C(=O)N2[C@@H](C(=O)O)C(C)(C)S[C@H]12
Neratinib	CCOc1cc2ncc(C#N)c(Nc3ccc(OCc4ccccn4)c(Cl)c3)c2cc1NC(=O)/C=C/CN(C)C
Olmutinib	C=CC(=O)Nc1cccc(Oc2nc(Nc3ccc(N4CCN(C)CC4)cc3)nc3ccsc23)c1
Omeprazole	COc1ccc2[nH]c([S+]([O-])Cc3ncc(C)c(OC)c3C)nc2c1
Orlistat	CCCCCCCCCCC[C@@H](C[C@@H]1OC(=O)[C@H]1CCCCCC)OC(=O)[C@H](CC(C)C)NC=O
Osimertinib	C=CC(=O)Nc1cc(Nc2nccc(-c3cn(C)c4ccccc34)n2)c(OC)cc1N(C)CCN(C)C
Pantoprazole	COc1ccnc(C[S+]([O-])c2nc3cc(OC(F)F)ccc3[nH]2)c1OC
Phenoxybenzamine	CC(COc1ccccc1)N(CCCl)Cc1ccccc1
Phenoxymethylpenicillin	CC1(C)S[C@@H]2[C@H](NC(=O)COc3ccccc3)C(=O)N2[C@H]1C(=O)O
Propylthiouracil	CCCc1cc(=O)[nH]c(=S)[nH]1
Rabeprazole	COCCCOc1ccnc(C[S+]([O-])c2nc3ccccc3[nH]2)c1C
Rasagiline	C#CCN[C@@H]1CCc2ccccc21
Relebactam	O=C(NC1CCNCC1)[C@@H]1CC[C@@H]2CN1C(=O)N2OS(=O)(=O)O
Rivastigmine	CCN(C)C(=O)Oc1cccc([C@H](C)N(C)C)c1
Saxagliptin	N#C[C@@H]1C[C@@H]2C[C@@H]2N1C(=O)[C@@H](N)C12CC3CC(CC(O)(C3)C1)C2
Selegiline	C#CCN(C)[C@H](C)Cc1ccccc1
Selinexor	O=C(/C=C\n1cnc(-c2cc(C(F)(F)F)cc(C(F)(F)F)c2)n1)NNc1cnccn1
Telaprevir	CCC[C@H](NC(=O)[C@@H]1[C@H]2CCC[C@H]2CN1C(=O)[C@@H](NC(=O)[C@@H](NC(=O)c1cnccn1)C1CCCCC1)C(C)(C)C)C(=O)C(=O)NC1CC1
Ticlopidine	Clc1ccccc1CN1CCc2sccc2C1
Tranylcypromine	N[C@@H]1C[C@H]1c1ccccc1.N[C@H]1C[C@@H]1c1ccccc1
Vigabatrin	C=CC(N)CCC(=O)O

Vildagliptin	N#C[C@@H]1CCCN1C(=O)CNC12CC3CC(CC(O)(C3)C1)C2
Warfarin	CC(=O)CC(c1ccccc1)c1c(O)c2ccccc2oc1=O
Zanubrutinib	C=CC(=O)N1CCC([C@@H]2CCNc3c(C(N)=O)c(-c4ccc(Oc5ccccc5)cc4)nn32)CC1
Fluorouracil	O=c1[nH]cc(F)c(=O)[nH]1
Narlaprevir	CCCC[C@H](NC(=O)[C@@H]1[C@@H]2[C@H](CN1C(=O)[C@@H](NC(=O)NC1(CS(=O)(=O)C(C)(C)C)CCCCC1)C(C)(C)C)C2(C)C)C(=O)C(=O)NC1CC1
Poziotinib	C=CC(=O)N1CCC(Oc2cc3c(Nc4ccc(Cl)c(Cl)c4F)ncnc3cc2OC)CC1
Sotorasib	C=CC(=O)N1CCN(c2nc(=O)n(-c3c(C)ccnc3C(C)C)c3nc(-c4c(O)cccc4F)c(F)cc23)[C@@H](C)C1
Adagrasib	C=C(F)C(=O)N1CCN(c2nc(OC[C@@H]3CCCN3C)nc3c2CCN(c2cccc4cccc(Cl)c24)C3)C[C@@H]1CC#N
